# Phospho-dependent Accumulation of GABA_B_Rs at Presynaptic Terminals after NMDAR Activation

**DOI:** 10.1016/j.celrep.2016.07.021

**Published:** 2016-08-04

**Authors:** Saad Hannan, Kim Gerrow, Antoine Triller, Trevor G. Smart

**Affiliations:** 1 Department of Neuroscience, Physiology and Pharmacology, University College London, Gower Street, London WC1E 6BT, UK; 2 Biologie Cellulaire de la Synapse, Inserm U1024, Institute of Biology, École Normale Supérieure (ENS), 46 rue d’Ulm, Paris 75005, France

**Keywords:** AMPK, bungarotoxin binding site, Ca^2+^ signaling, excitotoxicity, GABA receptors, GABA_B_ receptors, glutamate receptors, hippocampus, homeostatic signaling, lateral diffusion, NMDA receptors, phosphorylation, presynaptic terminal, quantum dots, receptor mobility, single-particle tracking, sushi domains

## Abstract

Here, we uncover a mechanism for regulating the number of active presynaptic GABA_B_ receptors (GABA_B_Rs) at nerve terminals, an important determinant of neurotransmitter release. We find that GABA_B_Rs gain access to axon terminals by lateral diffusion in the membrane. Their relative accumulation is dependent upon agonist activation and the presence of the two distinct sushi domains that are found only in alternatively spliced GABA_B_R1a subunits. Following brief activation of NMDA receptors (NMDARs) using glutamate, GABA_B_R diffusion is reduced, causing accumulation at presynaptic terminals in a Ca^2+^-dependent manner that involves phosphorylation of GABA_B_R2 subunits at Ser783. This signaling cascade indicates how synaptically released glutamate can initiate, via a feedback mechanism, increased levels of presynaptic GABA_B_Rs that limit further glutamate release and excitotoxicity.

## Highlights

• Presynaptic GABA_B_Rs are recruited to axon terminals by lateral diffusion
• Activated GABA_B_R1aR2 complexes become less mobile due to their sushi domains
• NMDAR activity recruits GABA_B_Rs to axon terminals by slowing their mobility
• GABA_B_R accumulation depends on intracellular Ca^2+^ and phosphorylation of GABA_B_R2^S783^

## Graphical Abstract

**Figure:**
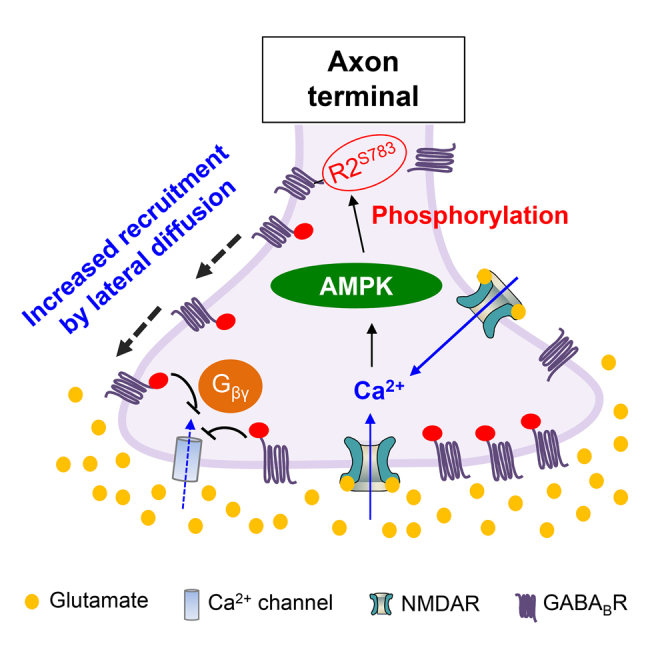

## Teaser

Hannan et al. report a GABA-mediated inhibitory homeostatic mechanism by which lateral diffusion of presynaptic GABA_B_ receptors and their accumulation on presynaptic excitatory terminals causes a reduction of presynaptic activity initiated by NMDA receptor activation. The underlying mechanism requires elevated intracellular Ca^2+^ and AMPK-dependent phosphorylation of presynaptic GABA_B_ receptor subunits on serine 783.

## Introduction

Maintaining spatio-temporal stability over neural network activity is important for brain function (Davis, 2006, Marder and Goaillard, 2006, Turrigiano, 1999). In particular, exercising tight control over excitatory transmission is important to avoid the consequences of excessive glutamate-mediated signaling, characterized by ischemic insults, traumatic brain injury, and epilepsy (Choi, 1987, Meldrum, 1994, Werner and Engelhard, 2007).

Several mechanisms have evolved to limit excessive neurotransmission, involving rapid changes to glutamate receptor conformation (e.g., desensitization) and reductions in synaptic glutamate concentration (e.g., uptake), to longer latency silencing of excitatory synapses, withdrawal of dendritic spines, and ablation of neurons (Paoletti et al., 2013, Traynelis et al., 2010). In addition, GABA, the principal inhibitory transmitter in the brain, activates signaling pathways that regulate cell excitability via ionotropic (GABA_A_ receptors [GABA_A_Rs]; Fritschy and Panzanelli, 2014, Moss and Smart, 2001) and metabotropic GABA receptors (GABA_B_Rs; Benke et al., 2015, Bettler and Tiao, 2006). Importantly, altering GABA receptor numbers at inhibitory synapses can indirectly regulate excitation, exemplified by the dispersal of GABA_A_Rs following NMDA receptor (NMDAR) activation (Muir et al., 2010).

Although increasing postsynaptic inhibition is effective at reducing excitability, targeting presynaptic terminals enables a precise input-selective approach by regulating neurotransmitter release. The close proximity of GABA_B_Rs to excitatory synapses makes them ideal candidates in this regard. Presynaptic GABA_B_Rs can inhibit voltage-gated Ca^2+^ channels (Bettler et al., 2004), and when activated by GABA spillover from nearby inhibitory synapses, these high-affinity receptors can inhibit glutamate release.

Prolonged NMDAR activation can also regulate GABA_B_R trafficking by increasing their internalization and degradation (Benke et al., 2012, Maier et al., 2010). This reduction in surface GABA_B_R numbers was unexpected and raised the question as to what homeostatic mechanisms exist to ensure the long-term stability of excitatory transmission, without ensuing excitotoxicity. For example, could inhibition be differentially affected by physiological activation of NMDARs at excitatory synapses? Furthermore, can mobile cell surface inhibitory receptors regulate synaptic transmission (Choquet and Triller, 2013)? In this context, diffusing presynaptic GABA_B_Rs could limit the release of glutamate, especially during ischemia (Cimarosti et al., 2009) and excitotoxicity (Benke, 2013). Although the lateral mobility of several receptors in the postsynaptic density has been characterized (Bürli et al., 2010, Choquet and Triller, 2013, Fernandes et al., 2010, Jaskolski and Henley, 2009, Ladépêche et al., 2014), little is known about the presynaptic lateral mobility of GABA_B_Rs around axonal membrane compartments (Ladepeche et al., 2013, Mikasova et al., 2008).

Here, we describe how activated GABA_B_Rs rapidly accumulate at presynaptic terminals by lateral diffusion, a feature that relies on their sushi domains (SDs). Brief NMDAR activation promotes GABA_B_R accumulation at presynaptic terminals after phosphorylation of the GABA_B_R2 subunits. Controlling presynaptic GABA_B_R mobility can thus form a basis for homeostatic regulation of excitatory transmission.

## Results

### Lateral Diffusion of GABA_B_Rs on Hippocampal Neurons

**Figure 1:**
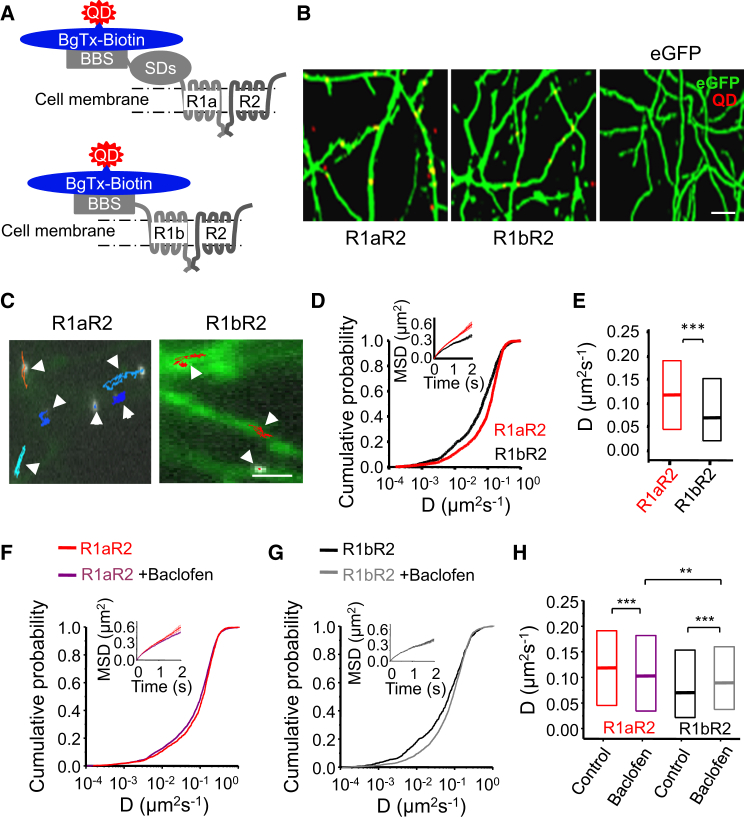
Single GABA_B_Rs Are Mobile on Hippocampal Cell Surface Membranes (A) GABA_B_R subunits with BBS, bound BgTx-biotin (BgTx-B), and QD655 coupled to streptavidin. Note sushi domains (SDs) in R1a. (B) Specific QD labeling occurs for cells expressing R1a^BBS^ (R1a) or R1b^BBS^ (R1b) with R2 and not for eGFP controls incubated in BgTx-B (4 μg/ ml; 2 min) and then QD (10 pM for 1 min at 37°C). The scale bar represents 5 μm. (C) QD trajectories (arrowheads) for single R1aR2 and R1bR2. The scale bar represents 2 μm. (D) Cumulative probabilities for diffusion coefficients, *D*, and mean square displacements (MSDs) (inset) for R1aR2 and R1bR2. (E) Box plot of the 25%–75% inter-quartile range (IQR) and median *D* values. (F) Cumulative probability distributions of R1aR2 *D* in control and +100 μM baclofen. Inset shows the MSD plots. (G) Cumulative probabilities for R1bR2 *D* in control and +baclofen. (H) Median *D* and IQR in control and +baclofen for the data in (F) and (G). ^∗∗^p < 0.01; ^∗∗∗^p < 0.001, KS test (see also Figures S1 and S2 and Movie S1).

To determine whether single GABA_B_Rs can diffuse to discrete synaptic membrane domains, we labeled them with quantum dots (QDs) by adding a “functionally silent” mimotope of the α-bungarotoxin (α-BgTx) binding site (BBS) to the start of the N-terminal domain of individual GABA_B_R subunits (Figure 1A). This allowed their visualization on the surface of hippocampal neurons.

The two isoforms of GABA_B_R1 (R1a and R1b) differ by two SDs in the N terminus of R1a, which are absent in R1b (Bettler and Tiao, 2006). Neurons expressing receptors with the BBS (R^BBS^) were labeled using biotinylated α-BgTx (α-BgTx-B), to which QD655-streptavidin (QD) can subsequently bind (Figure 1A). This reaction labeled both R1 subunits to equal extent and was highly specific as neurons transfected with cDNAs encoding for either eGFP or wild-type GABA_B_Rs (lacking a BBS) failed to bind QDs; moreover, incubating neurons with biotin-free α-Bgtx also failed to bind QD655 (<1% of control; Figures 1B and S1), thereby validating the use of the BBS for real-time labeling of GABA_B_Rs with QDs.

The extent of recombinant GABA_B_R expression in transfected neurons was assessed from K^+^ currents evoked by 10 or 100 μM baclofen. No difference in current density was observed between neurons expressing GABA_B_R1a^BBS^R2 with untransfected or eGFP-only controls at 2, 5, and 7 days post-transfection (p > 0.05; one-way ANOVA; Figures S2A and S2B), indicating there is no functional overexpression of cell surface GABA_B_Rs coupled to inwardly rectifying K^+^ (Kir) channels, even though levels of intracellular receptor were higher in GABA_B_R1a^BBS^R2-expressing neurons (p < 0.05; Figure S2C). This lack of functional overexpression could also reflect a limited supply of G proteins and/or Kir channels. However, beyond 7 days post-transfection, a trend toward increased baclofen-activated K^+^ currents is observed in transfected cells compared to controls. As our studies are conducted before this time point, any receptor overexpression would not confound the results. Furthermore, a similar time profile for GABA_B_R expression in neurons was obtained with R1b^BBS^R2 (Figures S2A and S2B).

Labeling of cell surface GABA_B_R1a^BBS^R2 and R1b^BBS^R2 (termed R1aR2 and R1bR2) expressed in cultured hippocampal neurons revealed lateral mobilities with a range of diffusion coefficients and confinement properties. R1aR2 were more mobile, traversing longer and less-confined tracks compared to the compact trajectories of R1bR2s (Figure 1C; Movie S1). Consistent with these profiles, R1aR2 display higher median diffusion coefficients (*D*; Table S1; p < 0.001, Kolmogorov-Smirnov [KS] test; Figures 1D and 1E) and are less confined (increased mean square displacement [MSD] plots; Figure 1D, inset) compared to R1bR2. Thus, under basal conditions, these receptors exhibit distinct diffusion profiles, highlighting an important role for the SDs present only in R1a.

### Activation Moderates the Lateral Mobility of GABA_B_Rs

Activated receptors can be rapidly removed from synapses and their entry restricted (Borgdorff and Choquet, 2002, Groc et al., 2004, Mikasova et al., 2008) to prevent excessive signaling. Chronic activation of recombinant R1bR2s with baclofen (100 μM for 1 hr) increased their lateral mobility (Pooler and McIlhinney, 2007), but to examine whether near-maximal GABA_B_R activation (Hannan et al., 2012) affects lateral mobility under physiological conditions, we studied hippocampal neurons exposed to 100 μM baclofen (chosen to maximally activate GABA_B_Rs) for <5 min. Activated R1aR2 traversed the cell surface more slowly than non-activated controls (Table S1; p < 0.001, KS test; Figures 1F and 1H). By contrast, activated R1bR2 receptors exhibited higher *D* values compared to non-activated counterparts (p < 0.001, KS test; Figures 1G and 1H). Despite affecting diffusion, baclofen did not alter the confinement of either R1aR2 (p > 0.05; Figure 1F, inset) or R1bR2 (Figure 1G, inset) compared to controls, with R1aR2 remaining less confined. Even though baclofen slowed R1aR2 and increased mobility of R1bR2, the median *D* in baclofen for R1aR2 was still higher than that for R1bR2 (p < 0.01, KS; Figure 1H). Thus, the mobility of these receptors is differentially regulated by activation.

### GABA_B_Rs Are Recruited to Presynaptic Terminals by Lateral Diffusion

Studying the lateral mobility of receptors over the entire cell surface, without discrimination, obscures membrane-domain-specific effects. R1aR2 and R1bR2 are known to play different roles in synaptic transmission (Gassmann et al., 2004, Guetg et al., 2009, Pérez-Garci et al., 2006), with R1aR2 the predominant presynaptic isoform and R1bR2 found mostly postsynaptically near excitatory synapses.

**Figure 2:**
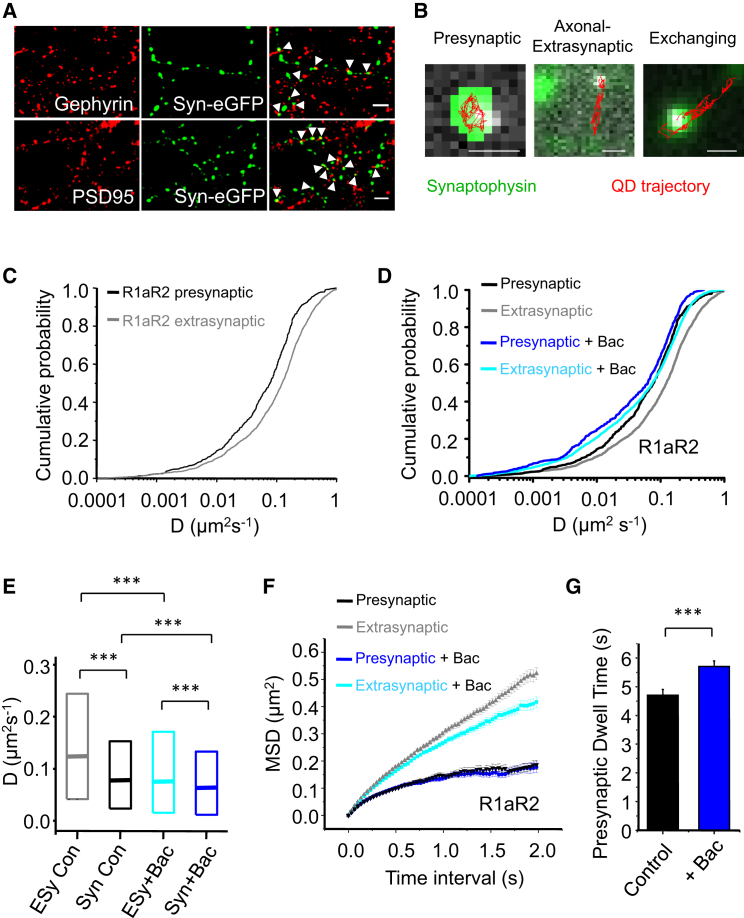
Recruiting GABA_B_Rs into Presynaptic Terminals by Diffusion (A) Cells showing close apposition of expressed synaptophysin (Syn)-eGFP puncta with endogenous gephyrin and PSD-95 (arrowheads). The scale bar represents 5 μm. (B) Trajectories (red) of presynaptic, axonal-extrasynaptic, and exchanging QD-labeled R1aR2s. The scale bar represents 1 μm. (C) Cumulative probability distributions for presynaptic and axonal-extrasynaptic R1aR2 diffusion coefficients. (D) Cumulative probabilities for presynaptic and axonal-extrasynaptic *D* in control and +100 μM baclofen (Bac). (E) Median *D* and IQR for presynaptic (Syn) and axonal-extrasynaptic (ESy) R1aR2s in control and +baclofen; ^∗∗∗^p < 0.001, KS test. (F) MSD plots for presynaptic and axonal-extrasynaptic R1aR2s in control and +baclofen. (G) Presynaptic dwell times for R1aR2s in control and +baclofen (n = 245 receptors; ^∗∗∗^p < 0.001, two-tailed unpaired t test). Data in all bar charts are means ± SEMs (see also Figure S2 and Movies S2 and S3).

To resolve membrane-domain-specific differences in receptor mobility, we studied GABA_B_Rs in presynaptic compartments with synaptophysin-eGFP (Syn-eGFP) (Tarsa and Goda, 2002). Syn-eGFP clusters formed predominantly in axons close to markers for excitatory (PSD-95) and inhibitory postsynaptic (gephyrin) structures (Figures 2A and S2D). GABA_B_Rs do not constitutively internalize into axons (Vargas et al., 2008), and therefore, lateral mobility would provide an important means of regulating their numbers at presynaptic terminals. For Syn-eGFP-positive axons and presynaptic terminals, QD-labeled R1aR2 clearly explored the surface by lateral diffusion (Movie S2). Receptors that frequented areas of axons that lacked Syn-eGFP are defined as *axonal-extrasynaptic*, a smaller fraction confined within Syn-eGFP clusters is classed as *presynaptic*, whereas the remainder transferred between these areas are defined as *exchanging* (Figure 2B; Movie S3). Axonal-extrasynaptic R1aR2s were more mobile (higher *D*) and less confined compared to presynaptic R1aR2s (p < 0.001, KS test; Figures 2C, 2E, and 2F). The large variations in the confinement of R1aR2s, evident from MSD plots (Figure 2F), may reflect transient interactions with anchoring proteins at presynaptic terminals. These results imply that R1aR2s are recruited to presynaptic terminals by diffusion (Movies S2 and S3).

### Activation Slows the Mobility of GABA_B_Rs at Presynaptic Terminals

As the overall mobility of R1aR2 was reduced by activation, we examined whether this applied to GABA_B_Rs in axonal-extrasynaptic and presynaptic domains of Syn-eGFP-expressing neurons. Baclofen (100 μM; <5 min) did not alter the relative distribution of R1aR2 among the three membrane domains, but their mobilities were reduced compared to non-activated controls (p < 0.001, KS test; Figures 2D and 2E), with axonal-extrasynaptic receptors showing more-pronounced retardation and confinement (Figure 2F). Consequently, the mean dwell time of presynaptic R1aR2 receptors was increased by baclofen (p < 0.001; Figure 2G). In addition, the ratio of exchanging receptors (between presynaptic and axonal-extrasynaptic domains) to total presynaptic receptors (Renner et al., 2009) was higher for control R1aR2s (0.81; n = 441) compared to after baclofen (0.68; n = 515), reflecting an increased residence of receptors at presynaptic terminals post-baclofen. Overall, these results indicate that activation of R1aR2s reduces their mobility at presynaptic terminals.

### GABA_B_R Isoforms Have Distinct Lateral Mobility on Axonal Membranes

We next compared the lateral mobility of R1bR2s on axons with R1aR2s. Although R1bR2s are predominantly postsynaptic, they are found in axonal membranes (Biermann et al., 2010). R1bR2s were mostly in the axonal-extrasynaptic domain, with a smaller population in presynaptic compartments and the remainder in the exchanging pool.

**Figure 3:**
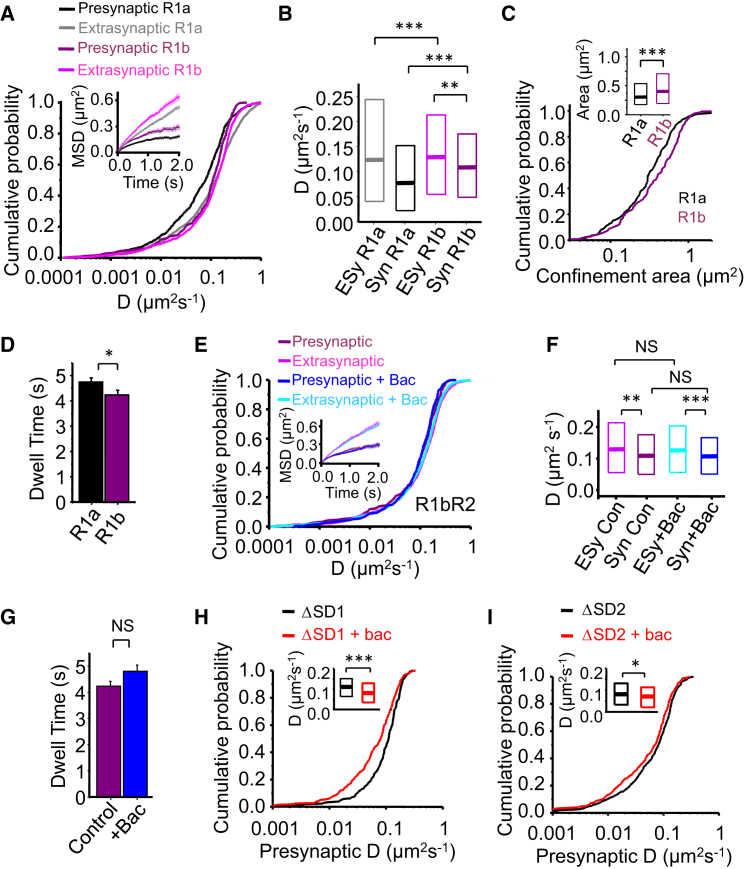
Diffusion Profiles for R1aR2s and R1bR2s at Presynaptic Terminals (A) Cumulative probabilities for *D* and MSD plots (inset) for presynaptic and axonal-extrasynaptic R1aR2s (R1a) and R1bR2s (R1b). (B) Median *D* and IQR for presynaptic (Syn) and axonal-extrasynaptic (ESy) receptors. (C) Cumulative probabilities (inset—median and IQR) for presynaptic confinement areas for R1aR2s (n = 185 receptors) and R1bR2s (n = 351 receptors). (D) Presynaptic dwell times for R1aR2s and R1bR2s. (E) Cumulative probabilities and MSDs (inset) for control presynaptic and axonal-extrasynaptic R1bR2s and +100 μM baclofen (Bac). (F) Median *D* and IQR for Syn and ESy R1bR2s in control and +baclofen. (G) Presynaptic dwell time of control R1bR2s and +baclofen (n = 229). (H) Cumulative probabilities for *D* (insets in H and I show median *D*) for presynaptic R1aR2s with the first sushi domain (ΔSD1) deleted in control and +100 μM baclofen. (I) Cumulative probabilities for *D* for presynaptic R1aR2 with the second sushi domain (ΔSD2) deleted in control and +100 μM baclofen. NS, not significant; ^∗^p < 0.05; ^∗∗^p < 0.01; ^∗∗∗^p < 0.001, KS test, two-tailed unpaired t test (see also Figure S3).

Axonal-extrasynaptic receptors were more mobile (higher *D*) than presynaptic R1bR2s (p < 0.01, KS; Figures 3A and 3B); both of these pools had higher *D* values compared to their R1aR2 equivalents (Figure 3B). The greater mobility of presynaptic R1bR2s was associated with reduced confinement compared to presynaptic R1aR2s (p < 0.001, KS test; Figures 3A and 3C), and consequently, the synaptic dwell time of presynaptic R1bR2s was also lower than that for R1aR2s (p < 0.05, unpaired two-tailed t test; Figure 3D). These results indicated that R1bR2s are less constrained than R1aR2s on axonal membranes.

The effect of agonist activation on R1bR2 lateral mobility was also assessed. Baclofen did not alter their relative localization, diffusion coefficients (p > 0.05; Figures 3E and 3F), or synaptic dwell times (p > 0.05; Figure 3G), in stark contrast to the slower mobility observed for R1aR2s (Figures 2D–2F).

These results indicate that single GABA_B_Rs are laterally mobile, with cell surface presynaptic receptors recruited from axonal-extrasynaptic areas. R1aR2s and R1bR2s have distinct mobility profiles at axon terminals, and only the trafficking of presynaptic R1aR2s is regulated by activation.

### SDs Regulate the Mobility of Presynaptic R1aR2s

We hypothesized that activation affected presynaptic R1aR2s mobility via the SDs, possibly interacting with extracellular partners. SDs are reported to also increase the residence of R1aR2s on the cell surface, compared to R1bR2s, by slowing their internalization (Hannan et al., 2012). We therefore studied whether the SDs affected R1aR2 mobility.

From the N terminus, deleting either the first (R1a^ΔSD1^R2) or second (downstream) SD (R1a^ΔSD2^R2), presynaptic R1a^ΔSD1^R2s or R1a^ΔSD2^R2s were still less mobile after baclofen compared to controls (p < 0.001; p < 0.05, KS test; Figures 3H and 3I), suggesting that only one SD need be present to slow R1aR2 mobility after activation.

Although the presynaptic and axonal-extrasynaptic diffusion coefficients for non-activated R1a^ΔSD1^R2 receptors were similar (p > 0.05, KS test; Figure S3), the *D* for presynaptic R1a^ΔSD1^R2s is higher than that for R1aR2s (p < 0.001, KS test; Figure S3), whereas the *D* for axonal-extrasynaptic R1a^ΔSD1^R2s is lower (Figure S3). These data further indicate the importance of SDs in determining the mobility of R1aR2s in axonal membranes and in accumulating receptors at presynaptic terminals. In comparison, R1a^ΔSD2^R2s behaved in a similar manner to R1a^ΔSD1^R2s (Figure S3), although the extent to which diffusion was reduced by baclofen was lower for the SD2 deletion, highlighting the crucial role SD1 plays in determining resting presynaptic GABA_B_R mobility. This suggests that, whereas the absence of the SDs in R1b renders GABA_B_Rs insensitive to changes in lateral diffusion upon activation, either one of the SDs can reduce diffusion of activated R1aR2s, and when combined, this effect is increased.

### Glutamate Receptors Modulate GABA_B_R Mobility

A primary role for R1aR2s at presynaptic glutamatergic terminals is to reduce glutamate release (Bettler et al., 2004). Presynaptic terminals and nearby GABA_B_Rs will be exposed to high transient levels of glutamate during excessive release. We therefore investigated whether such increases in glutamate concentration affected the mobility of single R1aR2s at presynaptic terminals, as a mechanism for mitigating excitotoxic events.

**Figure 4:**
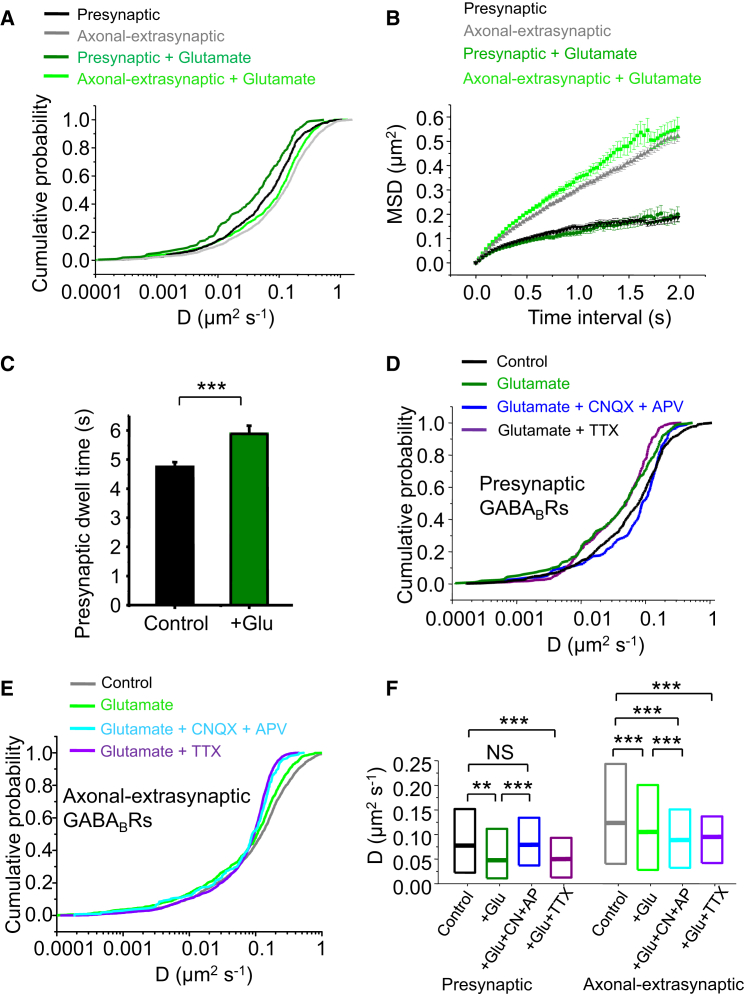
GluR Activation Reduces GABA_B_R Diffusion, Causing Recruitment to Presynaptic Terminals (A–C) Cumulative probabilities (A), MSDs (B), and mean presynaptic dwell times (C) for presynaptic and axonal-extrasynaptic R1aR2s in control Krebs and in 30 μM glutamate. (D and E) Cumulative probabilities for *D* of presynaptic (D) and axonal extrasynaptic (E) R1aR2s in control; +glutamate; +glutamate, CNQX (10 μM), and APV (100 μM); or +glutamate and tetrodotoxin (TTX) (0.5 μM). (F) Median *D* and IQR for presynaptic and axonal-extrasynaptic R1bR2s in control, +glutamate (Glu), or +glutamate with CNQX (CN) and APV (AP). ^∗∗^p < 0.01; ^∗∗∗^p < 0.001 KS test and two-tailed unpaired t test (see also Figures S4 and S5).

Applying 30-μM glutamate (chosen as it is ∼5-fold lower than measured extrasynaptic spillover levels but more than sufficient to activate perisynaptic NMDARs; Dzubay and Jahr, 1999, Rusakov and Kullmann, 1998) to neurons significantly reduced the mobility of axonal-extrasynaptic (p < 0.001, KS test) and presynaptic R1aR2s (p < 0.01, KS test; Figures 4A and 4F) compared to untreated cells. Glutamate did not alter the confinement patterns of either population (Figure 4B), and consistent with a reduced *D*, the presynaptic dwell time of GABA_B_Rs was increased (p < 0.01, unpaired two-tailed t test; Figure 4C). Moreover, the ratio of exchanging to total presynaptic receptors was also reduced by glutamate (0.63; n = 187), in accord with an accumulation of presynaptic GABA_B_Rs. Thus, like baclofen, but to a lesser extent (p < 0.001, KS; Figure S4), glutamate reduced the mobility of R1aR2s in axons.

To determine the glutamate receptor subtypes involved in accumulating terminal GABA_B_Rs, the AMPA receptor antagonist CNQX and NMDAR antagonist APV were used. Combining CNQX and APV prevented the reduction in mobility and accumulation of presynaptic GABA_B_Rs by glutamate. The *D* for presynaptic R1aR2s was not reduced by glutamate in CNQX and APV (p > 0.05, KS test) but increased compared to *D* for GABA_B_Rs exposed to glutamate alone (p < 0.001, KS test; Figures 4D and 4F). This indicated that presynaptic GABA_B_R mobility is modulated by activated ionotropic glutamate receptors.

By contrast, glutamate (in CNQX and APV) still reduced the mobility of axonal-extrasynaptic GABA_B_Rs compared to controls or glutamate alone (p < 0.001, KS test; Figures 4E and 4F), suggesting this effect is likely to be mediated by activated metabotropic glutamate receptors (data not shown). The reduction of GABA_B_R mobility was unlikely to be due to network-driven effects of glutamate because this was unaffected by tetrodotoxin (TTX) (p < 0.001, KS test; Figures 4D–4F).

### NMDAR Activation Recruits GABA_B_Rs to Presynaptic Terminals

We next studied which GluR isoform was involved in the accumulation of GABA_B_Rs at presynaptic terminals. In comparison with untreated neurons, NMDAR activation by glutamate in CNQX slowed the mobility of presynaptic GABA_B_Rs, reducing *D* (p < 0.001, KS; Figures S5A–S5D) to that for presynaptic GABA_B_Rs exposed to glutamate alone (p > 0.05). The dwell time was also increased (p < 0.001, one-way ANOVA; Figure S5D), equivalent to the dwell time in glutamate alone (p > 0.05, one-way ANOVA). These results strongly suggest that glutamate activation of NMDARs, and not AMPA receptors (AMPARs), retard the mobility of GABA_B_Rs.

**Figure 5:**
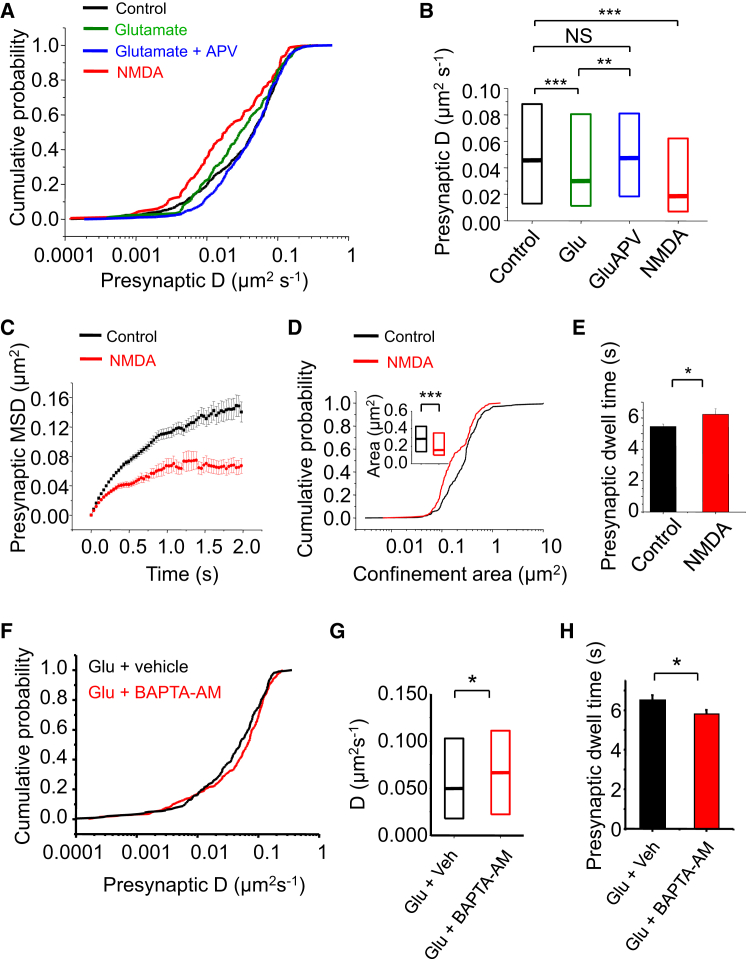
NMDAR Activation Increases Presynaptic Dwell Time of GABA_B_Rs (A) Cumulative probabilities of *D* for presynaptic R1aR2s in control, +30 μM glutamate (Glu), +30 μM glutamate and 100 μM APV, or +30 μM NMDA and 10 μM D-serine (NMDA). (B) Medians and IQR for presynaptic *D* of R1aR2 from (A). (C) MSDs for presynaptic GABA_B_Rs in control and in NMDA. (D) Cumulative probabilities of confinement areas for presynaptic GABA_B_Rs in control and NMDA (inset—median and IQR). (E) Presynaptic dwell times of GABA_B_Rs in control and NMDA. (F) Cumulative probabilities of presynaptic GABA_B_R *D* in glutamate for vehicle-treated controls or +20 μM BAPTA-AM. (G) Median and IQR of presynaptic *D* for GABA_B_Rs from (F). (H) Presynaptic dwell times of GABA_B_Rs in glutamate + vehicle (n = 192) or +BAPTA-AM (n = 336). ^∗^p < 0.05; ^∗∗∗^p < 0.001 (see also Figure S5).

These findings were corroborated by co-applying glutamate and APV, which prevented the reduction in *D* for presynaptic GABA_B_Rs compared to controls (Figures 5A and 5B; p > 0.05, KS test), whereas glutamate alone again reduced the lateral mobility of presynaptic GABA_B_Rs compared either to controls (p < 0.001, KS test) or to glutamate and APV (Figures 5A and 5B; p < 0.01, KS test).

To demonstrate unequivocally that NMDAR activation alone reduced presynaptic GABA_B_R diffusion, we co-applied NMDA and D-serine. This reduced GABA_B_R mobility with a corresponding decrease in *D* for presynaptic GABA_B_Rs (Figures 5A and 5B; p < 0.001, KS test), coupled with increased confinement (Figure 5C) and a reduced confinement area (Figure 5D; p < 0.001, KS test). As observed with glutamate alone, the presynaptic dwell time of GABA_B_Rs was significantly increased by NMDAR activation (Figure 5E; p < 0.05, two-tailed unpaired t test). These results together with the reduction in diffusion confirm that signaling via NMDARs accumulates GABA_B_Rs in presynaptic terminals by reducing their mobility.

### GABA_B_R Lateral Mobility Is Dependent on Elevating Internal Ca^2+^

Ca^2+^ permeation via NMDARs subsequently increasing internal Ca^2+^ is important for cellular signaling, including long-term potentiation (Lüscher and Malenka, 2012, Madison et al., 1991). To assess whether increasing internal Ca^2+^ is necessary for accumulating presynaptic GABA_B_Rs, glutamate was applied in the presence of the membrane permeant Ca^2+^ chelator, BAPTA-AM.

The diffusion of presynaptic R1aR2s was significantly faster with BAPTA-AM (Figures 5F and 5G; p < 0.05, KS). Values of *D* for R1aR2s in glutamate alone and glutamate plus vehicle were similar (Figure S5) but increased in glutamate and BAPTA-AM, approaching that for untreated R1aR2s (Figures S5E and S5F). Significantly, the presynaptic dwell time of GABA_B_Rs in glutamate was reduced by BAPTA-AM (Figure 5H; p < 0.05, unpaired two-tailed t test). Thus, elevated internal Ca^2+^ via NMDARs is required for accumulating GABA_B_Rs at presynaptic terminals via lateral diffusion.

### NMDAR Activation Causes the Accumulation of Native Presynaptic GABA_B_Rs

Having established that NMDAR activation reduces the mobility of presynaptic GABA_B_Rs via internal Ca^2+^, we studied the accumulation of *native* GABA_B_Rs at presynaptic terminals in permeabilized, cultured hippocampal neurons following NMDAR activation. Neurons were incubated in vehicle, glutamate, and NMDA and D-serine or glutamate and BAPTA-AM before fixation in paraformaldehyde (PFA), permeabilization, and labeling with antibodies for synaptophysin (Syn) and GABA_B_R2.

**Figure 6:**
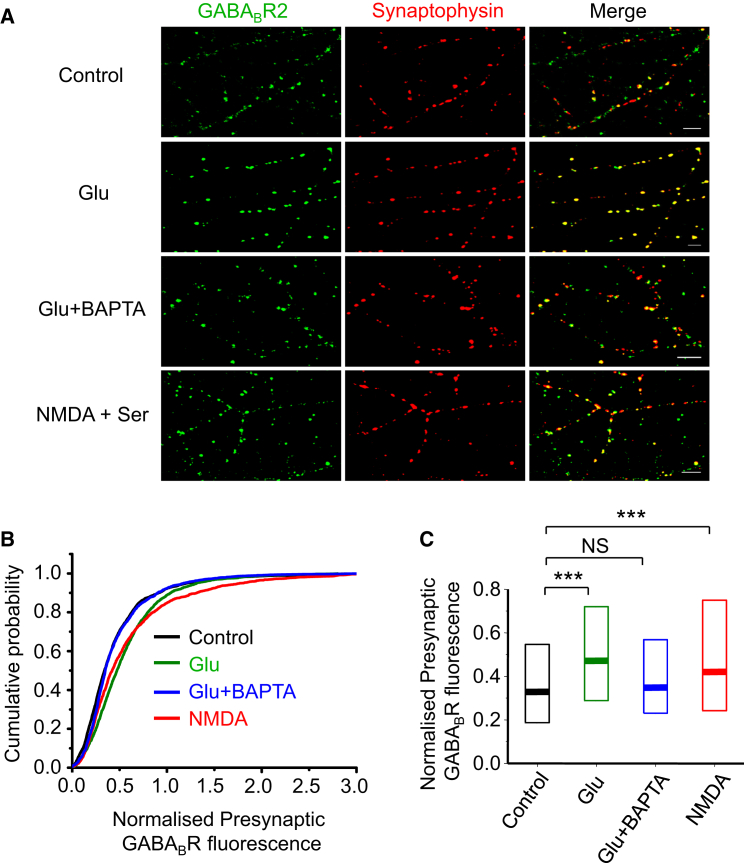
NMDAR Activation Increases GABA_B_Rs Accumulation at Presynaptic Terminals (A) Co-localization of native GABA_B_R2 and synaptophysin in permeabilized hippocampal neurons (14 DIV) in control, +30 μM glutamate (Glu), and +30 μM Glu and 20 μM BAPTA-AM, or +30 μM NMDA and 10 μM D-serine (NMDA). (B) Cumulative probabilities for presynaptic GABA_B_R2 fluorescence normalized to synaptophysin fluorescence in control, +glutamate, +glutamate and BAPTA-AM, and NMDA. (C) Median values for normalized GABA_B_R2 presynaptic fluorescence in control (n = 2,916 puncta), glutamate (n = 1,830), glutamate and BAPTA-AM (n = 1,832), and NMDA + serine (n = 1,535). ^∗∗∗^p < 0.001 (see also Figure S6).

Applying either glutamate or NMDA and D-serine (5 min) increased the fluorescence intensity for GABA_B_Rs (Figures 6A–6C; p < 0.001, Mann-Whitney [MW] test) at presynaptic, Syn-positive terminals. The co-application of BAPTA-AM with glutamate prevented this effect (p > 0.05; MW). In addition, glutamate also clearly increased cell surface presynaptic GABA_B_R staining assessed with BgTx-Alexa Fluor 555 labeling (Figures S6A–S6D). Together, these results from permeabilized and intact neurons are in accord with activated NMDARs increasing the accumulation of native GABA_B_Rs at presynaptic terminals.

### Presynaptic GABA_B_R Recruitment Requires Phosphorylation of Ser783 in R2

To examine how NMDAR activation recruits GABA_B_Rs to presynaptic terminals, we assessed the role of phosphorylation. GABA_B_Rs are substrates for protein kinases (Couve et al., 2002, Guetg et al., 2010) with 5′ AMP-activated protein kinase (AMPK) (Kuramoto et al., 2007) of particular interest because it phosphorylates GABA_B_R2 Ser783 following NMDAR activation (Terunuma et al., 2010). We investigated whether glutamate-induced accumulation of GABA_B_Rs is affected by R2 phosphorylation using the mutation S783A.

**Figure 7:**
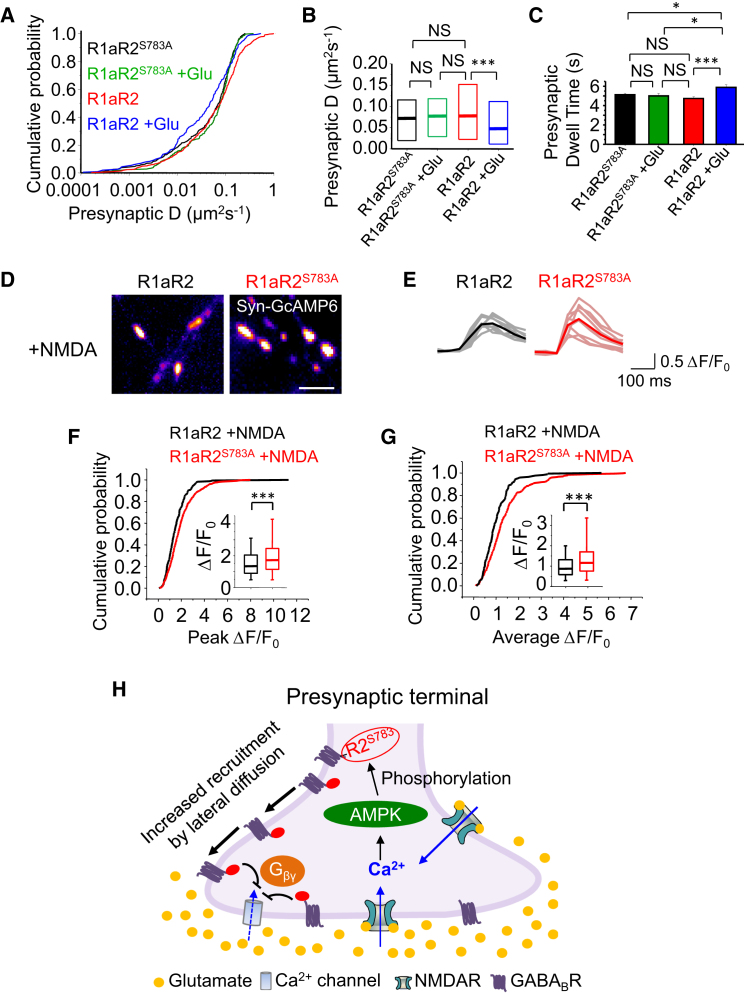
Glutamate Reduces GABA_B_R Mobility by Phosphorylating R2^S783^ (A) Cumulative probabilities for presynaptic *D* of R1aR2 and R1aR2^S783A^ in control and +30 μM glutamate (Glu). (B) Median *D* and IQR values from (A). (C) Presynaptic dwell times for R1aR2 and R1aR2^S783A^ with or without Glu. (D) Ca^2+^ signals evoked by 30 μM NMDA and 10 μM D-serine from Syn-GcAMP6 containing presynaptic terminals with either R1aR2 or R1aR2^S783A^. The scale bar represents 2 μm. (E) Ca^2+^ transients from single presynaptic terminals expressing R1aR2 or R1aR2^S783A^. Averaged transients are shown as dark lines. (F) Distribution for maximum peak Ca^2+^ transients from presynaptic terminals expressing R1aR2 or R1aR2^S783A^ (inset—5%–95% range, IQR, and median values). (G) Cumulative probabilities for average Ca^2+^ transients from presynaptic terminals expressing R1aR2 or R1aR2^S783A^ mutants (inset—5%–95% range, IQR, and median values; ^∗∗∗^p < 0.001). (H) Mechanism for homeostatic inhibitory control of transmitter release at excitatory terminals. NMDAR activation increases Ca^2+^ in presynaptic terminals and activates AMPK. Phosphorylation of R2^S783^ by AMPK increases lateral recruitment of GABA_B_Rs into the presynaptic terminal from axonal membranes. Activation of GABA_B_Rs by GABA spillover reduces Ca^2+^ entry into the terminals (via G_βγ_ signaling), thereby reducing neurotransmitter release from presynaptic boutons (see also Figures S6 and S7).

The diffusion of presynaptic R1aR2^S783A^ in control and in glutamate was similar to that for untreated R1aR2s (Figures 7A and 7B; p > 0.05, KS) but higher compared to R1aR2s in glutamate-treated neurons (p < 0.001; KS). This was reflected by shorter presynaptic dwell times for R1aR2^S783A^ in control and in glutamate, and untreated R1aR2s, compared to R1aR2s in glutamate (Figure 7C; p < 0.05, unpaired t test). These results suggest that phosphorylating S783 after NMDAR activation is critically important for accumulating GABA_B_Rs at presynaptic terminals.

The role of phosphorylation in the recruitment of native presynaptic GABA_B_Rs by glutamate was also studied in cultured hippocampal neurons using immunolabeling with a GABA_B_R2 phospho (p)-783-specific antibody. Glutamate increased fluorescence intensity labeling with p783 in Syn co-labeled presynaptic terminals (Figures S6E–S6G; p < 0.001, MW test). In addition, glutamate failed to increase the presynaptic accumulation of phospho-mutant GABA_B_Rs on the cell surface (Figures S6A, S6C, and S6D), and consistent with this, the S783A mutation reduced the localization of GABA_B_Rs at presynaptic terminals (Figure S7). These data corroborate the QD experiments, suggesting phosphorylation of S783 is key to glutamate-induced accumulation of GABA_B_Rs at presynaptic terminals.

### GABA_B_R2^S783A^ Potentiates Presynaptic NMDAR-Mediated Ca^2+^ Signaling

To understand how NMDAR activation and Ca^2+^ signaling combine to affect GABA_B_R mobility, we monitored Ca^2+^ signals in hippocampal neurons expressing the genetically encoded Ca^2+^ sensor GCaMP6 fused to Syn (Syn-GCaMP6Fast; Zhao et al., 2011) with either R1aR2 or R1aR2^S783A^. Basal Ca^2+^ levels were similar, but after applying NMDA and D-serine to R1aR2^S783A^-expressing neurons, the maximum peak Ca^2+^ transients in presynaptic terminals was elevated compared to neurons expressing R1aR2 (p < 0.001, MW test; Figures 7D–7F). In addition, the mean amplitude of Ca^2+^ transients was also greater for R1aR2^S783A^ neurons compared to R1aR2s (p < 0.001, MW test; Figure 7G).

This NMDAR-driven comparative increase in Ca^2+^ signaling was unexpected for neurons expressing R1aR2^S783A^. We had predicted that the increased Ca^2+^ signal would reduce *D* and increase the dwell time for GABA_B_Rs at presynaptic terminals, but neither change occurred (Figures 7B and 7C). Therefore, although increased Ca^2+^ influx recruits GABA_B_Rs to the presynaptic terminal, it is less effective in recruiting mutant (S783A) GABA_B_Rs, suggesting phosphorylation of S783A is the critical factor and must be “downstream” in the signaling pathway for accumulating presynaptic GABA_B_Rs. It is also conceivable that a reduction in GABA_B_R numbers at the presynaptic terminal, as a result of S783A (Figure S7), may be responsible for reduced presynaptic inhibition, leading to an increase in terminal Ca^2+^ flux via NMDARs and voltage-gated Ca^2+^ channels. Together, these results highlight the critical role of GABA_B_R phosphorylation for presynaptic receptor accumulation, an important facet in reducing neurotransmitter release after the activation of NMDARs.

## Discussion

Lateral diffusion is important for distributing receptors in postsynaptic membranes (Choquet and Triller, 2013). Fluorescence recovery after photobleaching suggests GABA_B_Rs are mobile on Cos7 cells and hippocampal neurons (Pooler and McIlhinney, 2007), but whether lateral mobility is important for accumulating and dispersing *presynaptic* receptors is poorly understood (Gomez-Varela and Berg, 2013). Although axonal and dendritic membranes have similar properties, there will be more physical constraints for diffusion in the presynaptic membrane, given the specialist role it plays in neurotransmitter release. Here, we demonstrate the importance of lateral diffusion as a mechanism for accumulating GABA_B_Rs at axon terminal membranes for eventual modulation of excitatory transmitter release. By studying the diffusion of single GABA_B_Rs with QDs, their recruitment into axonal membranes can be visualized and the underlying mechanisms examined.

Lateral diffusion of GABA_B_Rs was resolved by inserting a BBS into GABA_B_Rs, enabling single-particle tracking with reporter QDs. Such BgTx conjugates have been used for tracking nicotinic acetylcholine receptor α3 and α7 subunits (Bürli et al., 2010, Fernandes et al., 2010). The BBS mimotope (13 amino acids compared to eGFP, which is ∼240 amino acids) enables fast labeling and imaging that avoids complications with receptor internalization from the cell surface. The insertion of the BBS into R1a, R1b, or R2 subunits neither altered the trafficking nor function of GABA_B_Rs (reviewed in Hannan et al., 2013); its high affinity in R1a (Hannan et al., 2011) and R1b (Hannan et al., 2012) for BgTx allowed the specific labeling of GABA_B_Rs using titrated amounts of QDs. This avoids any confounds that might result from non-specific interactions between the polyethylglycol coating of the QDs with biological membranes. The smaller size of BgTx (∼24 nm^3^) compared to primary and secondary antibody complexes (∼500 nm^3^; Hannan et al., 2013) that are routinely used in QD imaging also makes this method suitable for monitoring diffusion where space constraints exist, such as at synapses.

The main subtypes of GABA_B_Rs, R1aR2s and R1bR2s, have distinct physiological and pathophysiological roles (Gassmann et al., 2004, Guetg et al., 2009, Pérez-Garci et al., 2006) and differ by the two SDs in R1a (Hawrot et al., 1998). Studies of GABA_B_R knockout models have identified a presynaptic role for R1a at glutamatergic terminals, whereas both R1a and R1b form postsynaptic receptors. Monitoring diffusion of these receptor subtypes revealed distinctive mobility profiles, with R1a being less mobile than R1b. The inability of R1b to become “trapped” at presynaptic terminals in response to GABA_B_R activation (unlike R1a) established the SDs as key modulators of lateral mobility in this study. This is in addition to their reported roles in transport (Biermann et al., 2010, Vigot et al., 2006) and cell surface stability (Hannan et al., 2012). For accumulating R1a at axon terminals, SD1 nearest the N-terminal is critical, whereas either SD (SD1/2) is capable of slowing R1a mobility after agonist activation. SD1 is most likely to reduce lateral diffusion by transiently interacting with protein partners in the terminal domain, and after agonist activation, presumed changes to receptor conformation may allow either SD to establish interactions, further reducing GABA_B_R diffusion at the presynaptic terminal.

Reducing GABA_B_R mobility and increasing presynaptic dwell time following activation could be significant in a physiological context. This would provide a mechanism for accumulating presynaptic GABA_B_Rs for controlling neurotransmitter release. In this regard, GABA_B_Rs that are perisynaptic to inhibitory synapses will be activated by GABA released directly from interneurons; and at glutamatergic terminals, the high-affinity GABA_B_Rs will be activated by GABA spillover from neighboring GABAergic synapses, leading to an increased clustering at presynaptic terminals (Dittman and Regehr, 1997, Dutar and Nicoll, 1988, Fritschy et al., 1999, Hirono et al., 2001, Isaacson et al., 1993, Kulik et al., 2002, Scanziani, 2000).

This role for SDs as modulators of lateral diffusion is unique. Their extracellular location contrasts with other interacting proteins that are typically intracellular and modulate lateral diffusion of receptors via interactions with scaffold proteins (Bannai et al., 2009, Hausrat et al., 2015, Renner et al., 2009). SDs are likely to slow down GABA_B_R mobility at synapses by transient interactions with extracellular partners within the presynaptic terminal or with recently identified postsynaptic partners (trans-synaptic; Schwenk et al., 2016) to achieve the same purpose. The latter might also explain the paucity of interacting partners for the SDs reported to date (Blein et al., 2004).

Accumulating even relatively few “membrane stable” GABA_B_Rs at synapses is likely to be a crucial determinant for the efficacy of excitatory transmission and associated cellular plasticity. Several studies have investigated GABA_B_R trafficking after sustained (>5 min) activation of ionotropic GluRs. These studies reported reduced cell surface GABA_B_R levels after prolonged NMDAR activation caused by increased rates of internalization (Guetg et al., 2010, Terunuma et al., 2010) and/or increased lysosomal degradation (Maier et al., 2010) of GABA_B_Rs. However, the residence of cell surface receptors at synaptic membrane microdomains has not been investigated under resting conditions or in agonist-activated neurons. Here, by briefly activating NMDARs (<5 min) to replicate physiologically relevant conditions, the subsequent elevation of intracellular Ca^2+^ (Hardie et al., 2012, Hawley et al., 2005, Weisová et al., 2009, Woods et al., 2005) will lead to increased phosphorylation of GABA_B_R2 at S783 by AMPK, causing an increase in the density of GABA_B_Rs at presynaptic terminals by reducing their lateral mobility (Figure 7H).

Even using prolonged periods of NMDAR activation, increased cell surface expression of GABA_B_Rs and R2 subunit phosphorylation are observed at early time points (∼5 min; Terunuma et al., 2010), a feature also noted in a recent study that assessed the cell surface stability of GABA_B_Rs in response to activity-dependent changes over a short time period (Kantamneni et al., 2014). These reports are consistent with the increased presynaptic accumulation of GABA_B_Rs observed in our study. During prolonged pathophysiological conditions, including traumatic brain injury and ischemia, prolonged and sustained activation of NMDARs will likely cause the activation of the phosphatase PP2A (Terunuma et al., 2010), leading to dephosphorylation of S783, decreasing the number of surface GABA_B_Rs by increasing internalization and degradation.

The importance of phosphorylation in the recruitment of presynaptic GABA_B_Rs is highlighted by the AMPK phosphorylation mutant, S783, on GABA_B_R2, which shows reduced localization to presynaptic terminals in response to glutamate. Interestingly, Ca^2+^ signaling was elevated in response to NMDAR activation in neurons expressing R2^S783A^. This most likely reflects a reduction in presynaptic GABA_B_Rs, as lateral diffusion is impaired by R2^S783A^ and is unlikely to be due to internalization with such a brief exposure to NMDA (Hannan et al., 2011).

We would propose the following model for GABA_B_R mobility at presynaptic excitatory synapses (Figure 7H). Pre- or postsynaptic NMDAR activation would initiate Ca^2+^ influx via NMDAR channels (Banerjee et al., 2016, Duguid and Smart, 2009) and, eventually, either directly or by retrograde transmitter release (Bouvier et al., 2015, Casado et al., 2000, Duguid and Smart, 2004), increase terminal Ca^2+^ levels following Ca^2+^ channel activation and/or by internal Ca^2+^ release. In terminal membranes, this would initially increase glutamate release (Bouvier et al., 2015) but also enable AMPK activation to phosphorylate S783 on R2 subunits (Kuramoto et al., 2007), slowing GABA_B_R mobility in the terminal membrane and promoting receptor accumulation to increase presynaptic inhibition once GABA_B_Rs are activated. Reducing the internal Ca^2+^ rise, or ablating phosphorylation at R2^S783^, is sufficient to reduce GABA_B_R accumulation. The rise in internal Ca^2+^, caused by NMDA, in neurons expressing phosphorylation mutant R2^S783A^ and the failure of this increase to affect receptor mobility strongly suggests that internal Ca^2+^ effects are mediated by phosphorylation of S783 and that this is the critical determinant of GABA_B_R mobility.

Thus, linking NMDAR activation with a signaling pathway involving internal Ca^2+^ and phosphorylation of GABA_B_Rs that reduces the lateral mobility and increases the recruitment of GABA_B_Rs to the presynaptic terminal membrane is a potentially powerful homeostatic mechanism for preventing excessive signaling and glutamate-mediated excitotoxicity.

## Experimental Procedures

For further details, see Supplemental Information. The GABA_B_R1 isoforms (R1a^BBS^ and R1b^BBS^) containing a BBS, a flag-tagged GABA_B_R2 (R2), R1a^BBS^ with SD deletion (R1a^ΔSD1^ or R1a^ΔSD2^), an R2^S783A^ mutant, and pEGFP-C1 have been described previously (Hannan et al., 2011, Hannan et al., 2012, Kuramoto et al., 2007). All drugs and chemicals were acquired from Sigma unless specified otherwise. All experiments were performed in accordance with the UK Animals (Scientific Procedures) Act 1986. Dissociated hippocampal cultures were prepared from embryonic day 18 (E18) Sprague-Dawley rat embryos and transfected at 7 days in vitro (DIV) as described previously (Hannan et al., 2011). For QD labeling, at 12–14 DIV, BBS-containing GABA_B_Rs were incubated in 4 μg/ml BgTx-B (Life Technologies) for 2 min at 37°C before incubation with QD655 conjugated to streptavidin (Life Technologies). For immunostaining, permeabilized neurons were incubated in primary antibodies (GABA_B_R2 [Neuromab], phospho-783 GABA_B_R2 [Santa Cruz Biotechnology], Syn [Abcam], PSD95 [Neuromab], and gephyrin [Synaptic Systems]) followed by secondary antibodies conjugated with Alexa 488, 555, or 594 (Life Technologies) prior to imaging. For cell surface labeling, BBS-containing GABA_B_Rs were incubated in 4 μg/ml BgTx Alexa Fluor 555 (Life Technologies) for 10 min at room temperature (RT). For Ca^2+^ imaging, Syn-GcAMP6Fast Ca^2+^ transients were captured in the presence of brief applications of NMDA and D-serine before analysis of signals using Matlab. Whole-cell electrophysiology was performed as described in Supplemental Information.

## Author Contributions

S.H. and T.G.S. designed the project and wrote the manuscript. S.H. undertook the electrophysiology, quantum dot, and imaging experiments and analyzed the data. K.G. processed the presynaptic markers for localization. A.T. contributed to the algorithms used for analyzing the quantum dot trajectories. All contributed to the writing of the manuscript.

## Supplemental Information

Document S1. Supplemental Experimental Procedures, Figures S1–S7, and Table S1

Movie S1. Real-Time Lateral Diffusion of Quantum-Dot-Tagged GABA_B_ Receptors on Neuronal Membranes, Related to Figure 1

Movie S2. Real-Time Lateral Diffusion of Quantum-Dot-Tagged GABA_B_ Receptors on Axonal Membranes Labeled with Synaptophysin-eGFP, Related to Figure 2

Movie S3. Real-Time Lateral Diffusion of Quantum-Dot-Tagged GABA_B_ Receptors on Presynaptic Terminals Labeled with Synaptophysin-eGFP along with Axonal Extrasynaptic and Exchanging Receptors, Related to Figure 2

Document S2. Article plus Supplemental Information
