# Supplementary material for: Phospho-dependent Accumulation of GABABRs at Presynaptic Terminals after NMDAR Activation
Source: Cell Rep. 2016 Aug 4;16(7):1962–73. doi: 10.1016/j.celrep.2016.07.021 (PMC4987283; doi:10.1016/j.celrep.2016.07.021)
Supplement: Document S1. Supplemental Experimental Procedures, Figures S1–S7, and Table S1 [file mmc1.pdf]

Cell Reports, Volume *16*

Supplemental Information

Phospho-dependent Accumulation of $GABA_B$Rs at Presynaptic Terminals after NMDAR Activation

Saad Hannan, Kim Gerrow, Antoine Triller, and Trevor G. Smart

Phospho-dependent accumulation of $GABA_B$Rs at presynaptic terminals after NMDAR activation

Saad Hannan[1], Kim Gerrow[2], Antoine Triller[2], and Trevor G Smart[1]*

[1]Department of Neuroscience, Physiology and Pharmacology, University College London, Gower Street, London WC1E 6BT, UK

[2]Biologie Cellulaire de la Synapse, Inserm U1024, Institute of Biology, École Normale Supérieure (ENS), 46 rue d'Ulm, Paris 75005, France

*Corresponding author – t.smart@ucl.ac.uk

Abbreviated title – Mobility of presynaptic $GABA_B$Rs during excitatory transmission

Supplemental Figures

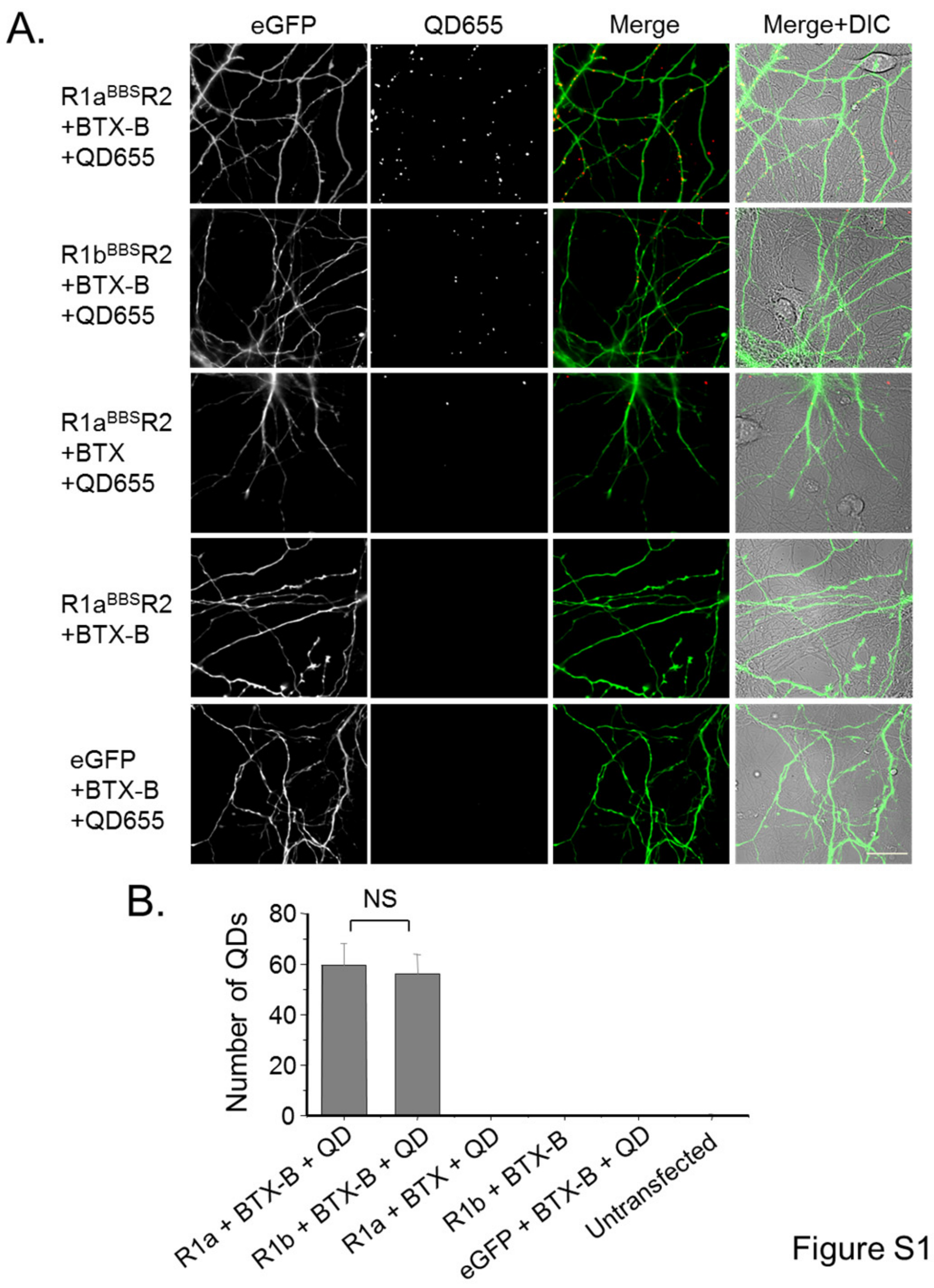

Figure S1

Figure S1. Specificity of QD labeling (related to Figure 1).

(A) Images of neurons expressing the bungarotoxin binding site (BBS) tag in $R1a^{BBS}$ or $R1b^{BBS}$ with R2 and eGFP showing labeling with quantum dots (QDs) only when incubated in 4 µg/ml bungarotoxin biotin (BgTx-B) for 2 min followed by 10 pM QD 655 coupled to streptavidin (QD) for 1 min at 37°C. Neurons were not labeled with QDs when they were incubated with unlabeled bungarotoxin (lacking the biotin linker) or with just BgTx-B. In addition, neurons expressing just eGFP showed no staining when incubated in BgTx-B and QDs, confirming the high specificity of the labeling reaction. Scale bar = 10 µm. (B) Bar-chart of bound QDs for the conditions in (A) plus untransfected cells.

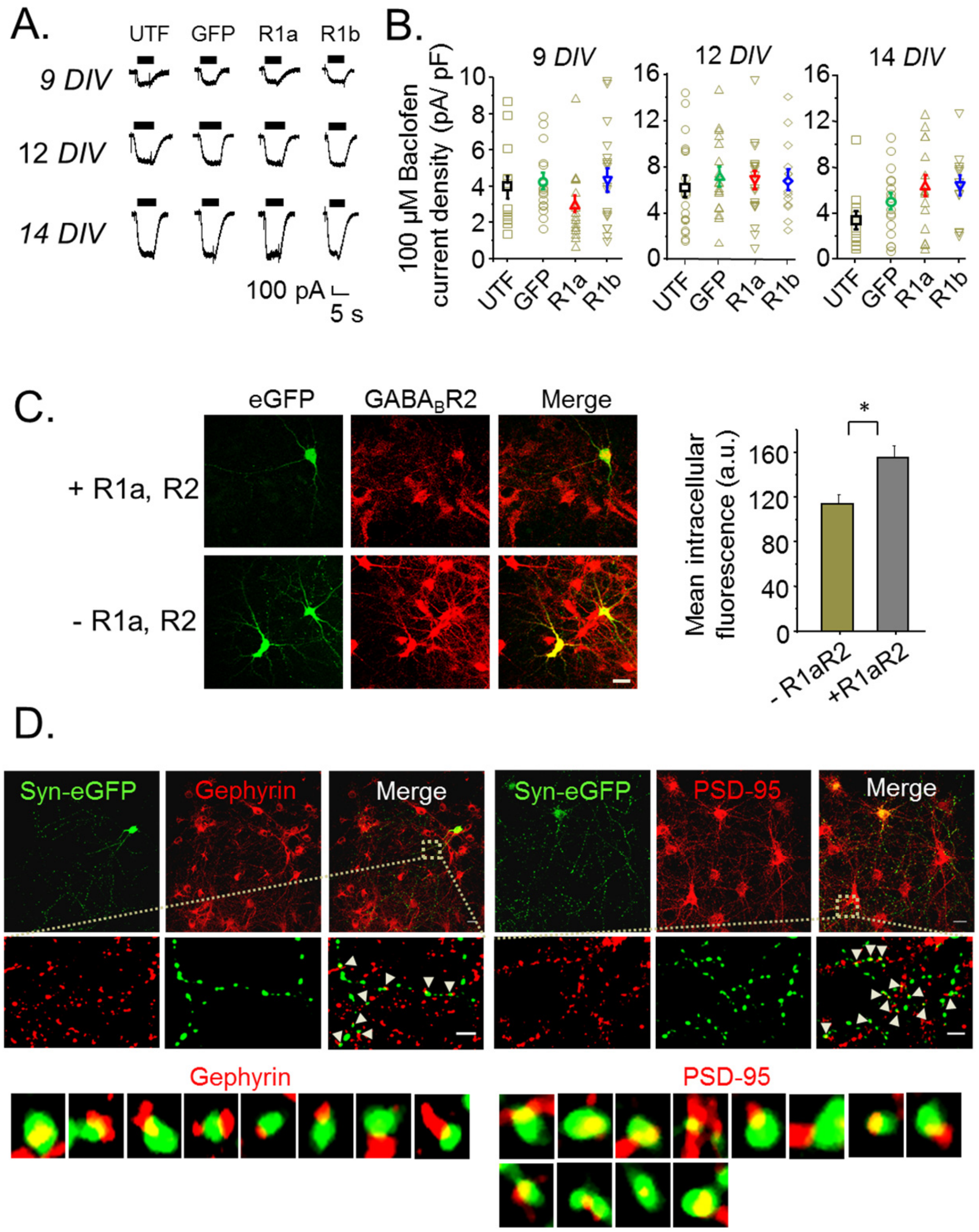
A.
UTF
GFP
R1a
R1b
9 DIV
12 DIV
14 DIV
100 pA
5 s
B.
9 DIV
12 DIV
14 DIV
100 μM Baclofen current density (pA/ pF)
UTF
GFP
R1a
R1b
C.
eGFP
GABABR2
Merge
+ R1a, R2
- R1a, R2
Mean intracellular fluorescence (a.u.)
*
-R1aR2
+R1aR2
D.
Syn-eGFP
Gephyrin
Merge
Syn-eGFP
PSD-95
Merge
Gephyrin
PSD-95

Figure S2

Figure S2. Expression levels of recombinant $GABA_BRs$ and Synaptophysin-eGFP (related to Figures 1 and 2)

(A) Representative 100 µM baclofen (bac)-activated K^+ currents recorded from untransfected (UTF), eGFP transfected (GFP), and eGFP, R1a (R1a) or R1b (R1b) and R2 transfected hippocampal neurons at 9, 12 and 14 days *in vitro* (*DIV).* Neurons were transfected at 7 *DIV* then left for 2, 5 and 7 days before whole-cell recording. (B) 100 µM baclofen (bac)-activated K^+ current densities from untransfected (UTF), eGFP transfected (GFP), and eGFP, R1a (R1a) or R1b (R1b) and R2 transfected hippocampal neurons at 9, 12 and 14 *DIV.* Note that the current densities evoked by baclofen are not different between untransfected, eGFP only transfected, and eGFP and R1a/R1bR2 neurons at all days tested using one-way ANOVA; however, there is a trend towards increased K^+ current in R1a/R1bR2 cDNA transfected cells at 14 *DIV*. (C) Images showing expression of $GABA_BRs$ in untransfected, neurons transfected with cDNAs for eGFP (without (-) R1a, R2) or eGFP with R1a and R2 (+ R1a, R2) at 14 *DIV*. Neurons were transfected at 7 *DIV* and fixed at 14 *DIV,* permeabilised and labeled using primary antibodies against $GABA_BR2$ and secondary antibodies conjugated to Alexa Fluor 594 before imaging. The bar-chart shows the mean fluorescence intensity of intracellular $GABA_BRs$ in GFP-positive dendrites. $P<0.05$, two-tailed unpaired t test, n = 7-8. Scale bar 10 µm (D) Images of hippocampal neurons expressing synaptophysin-GFP colocalized with the inhibitory postsynaptic marker, gephyrin, and the excitatory postsynaptic marker, PSD-95 from Figure 2A. Arrowheads show the close apposition of the synaptophysin-eGFP puncta with endogenous gephyrin and PSD-95. The bottom panel shows high resolution images of the synaptic contacts between synaptophysin-eGFP and postsynaptic markers for the arrowheads depicted above. Scale bars 20 µm and 5 µm.

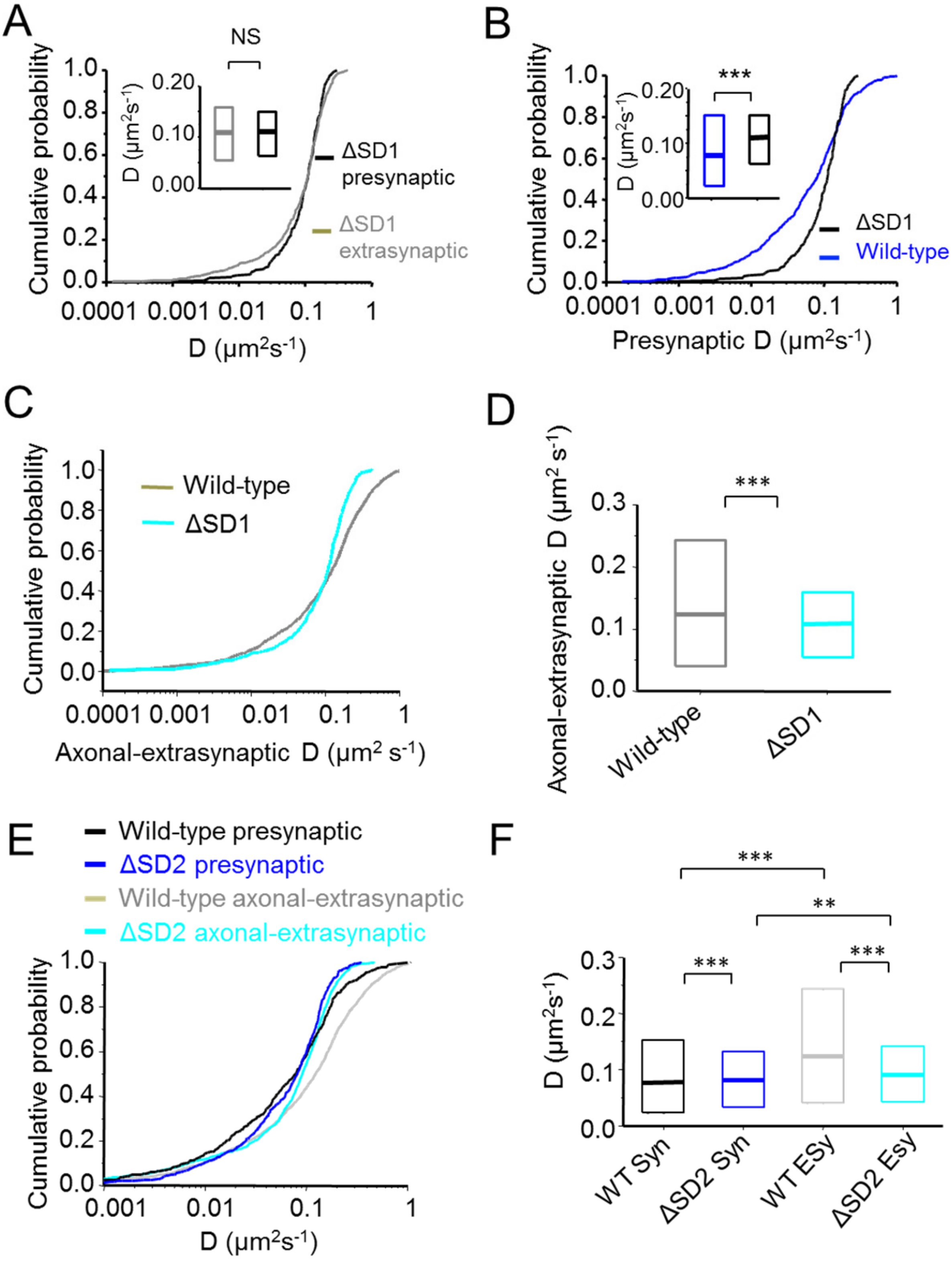
A
NS
Cumulative probability
D (μm²s⁻¹)
ΔSD1 presynaptic
ΔSD1 extrasynaptic
B
***
Presynaptic D (μm²s⁻¹)
ΔSD1
Wild-type
C
Wild-type
ΔSD1
Axonal-extrasynaptic D (μm² s⁻¹)
D
Axonal-extrasynaptic D (μm² s⁻¹)
Wild-type
ΔSD1
E
Wild-type presynaptic
ΔSD2 presynaptic
Wild-type axonal-extrasynaptic
ΔSD2 axonal-extrasynaptic
F
**
WT Syn
ΔSD2 Syn
WT ESy
ΔSD2 Esy

Figure S3

Figure S3. Deletion of the first Sushi Domain reduces lateral diffusion of axonal-extrasynaptic $GABA_BRs$ (related to Figure 3).

(A) Cumulative probability distributions of diffusion coefficients (*D*) for presynaptic and axonal-extrasynaptic N-terminal Sushi Domain deleted (ΔSD1) $R1a^{\Delta SD1}R2$ receptors. Inset shows median *D and IQR.* (B) Cumulative probabilities for *D* of presynaptic ΔSD1 receptors compared to R1aR2 (wild-type) controls. Inset shows median *D and IQR.* (C) Cumulative probabilities for *D* of axonal-extrasynaptic wild-type or ΔSD1. (D) Box-plot showing the IQR and median *D* for axonal-extrasynaptic wild-type and ΔSD1. (E) Cumulative probability distributions of *D* for presynaptic and axonal-extrasynaptic R1aR2 (wild-type) or C-terminal Sushi Domain deleted (ΔSD2) $R1a^{\Delta SD2}R2$. (F) Box-plot showing the IQR and median *D* for presynaptic (Syn) and axonal-extrasynaptic (Esy) wild-type (WT) and ΔSD2. NS – not significant, **P<0.01, ***P<0.001 KS test.

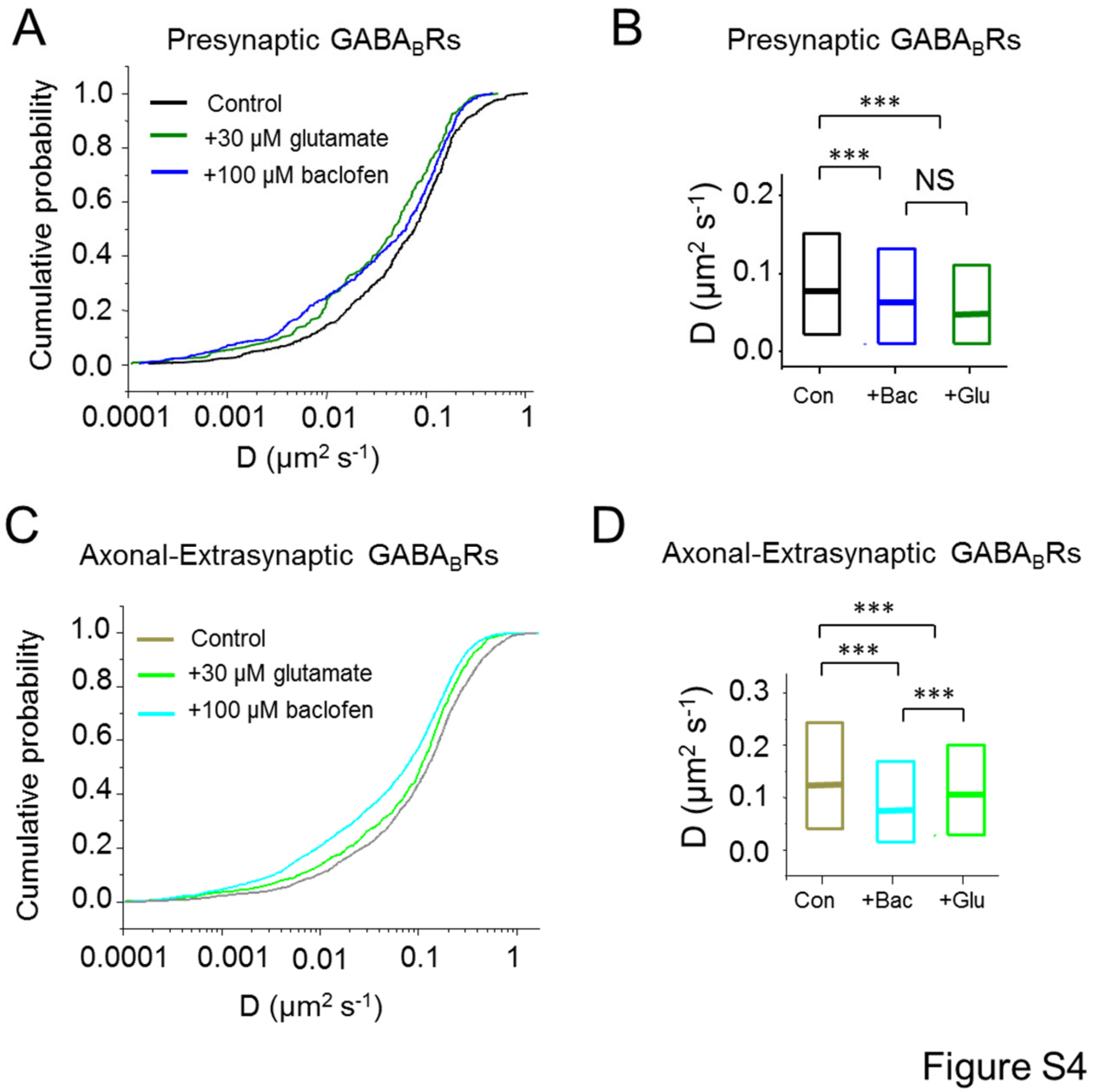

Figure S4. Baclofen causes a larger reduction in the mobility of axonal-extrasynaptic GABA$_B$Rs (related to Figures 2 and 4).

Cumulative probability distributions for presynaptic (A) and axonal-extrasynaptic (C) R1aR2 diffusion coefficients in the presence of 30 µM glutamate (Glu) or 100 µM baclofen (Bac). Box-plot showing the IQR and median *D* of presynaptic (B) and axonal-extrasynaptic (D) R1aR2 receptors. ***P<0.001, NS – not significant, KS test.

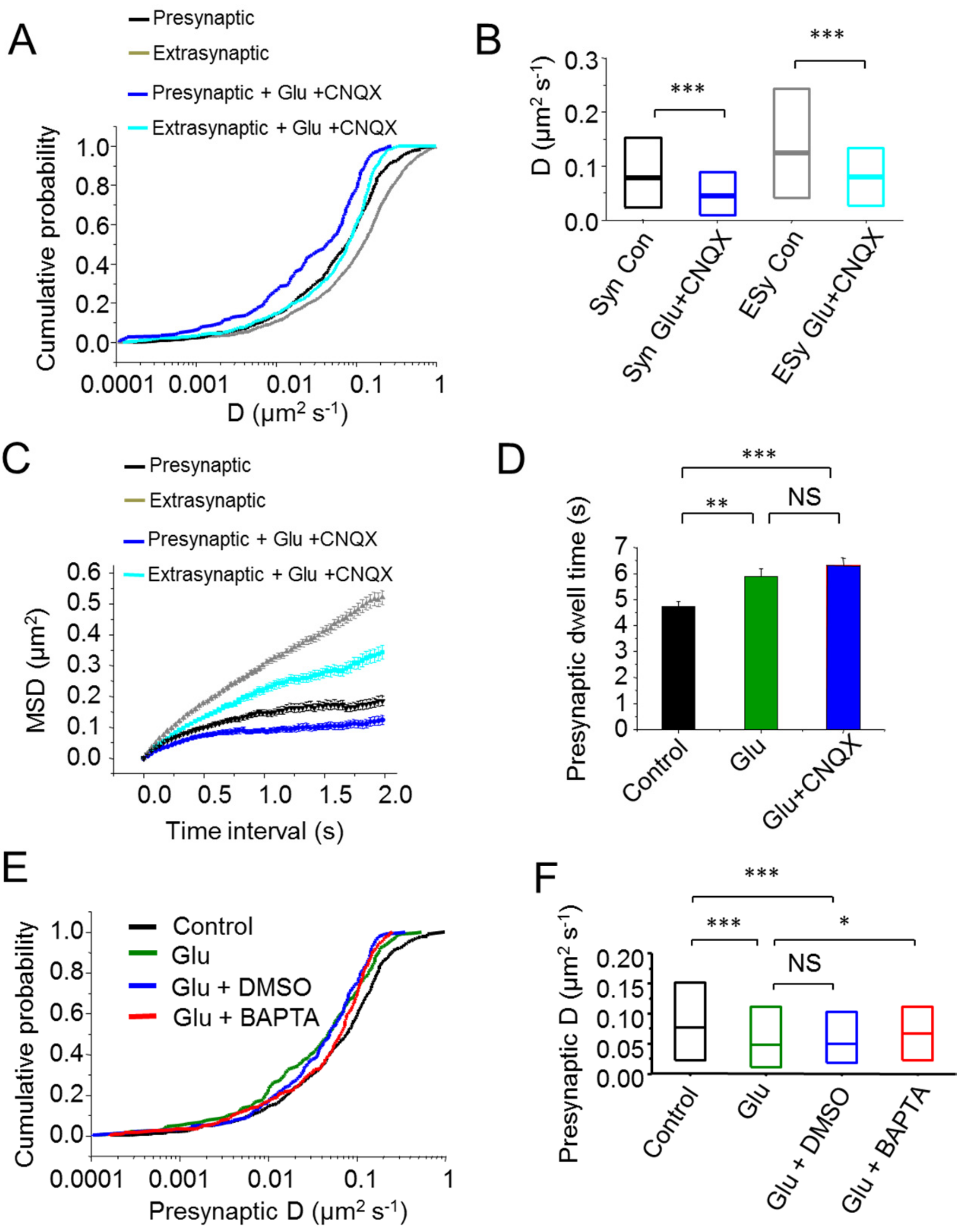
A
Presynaptic
Extrasynaptic
Presynaptic + Glu +CNQX
Extrasynaptic + Glu +CNQX
Cumulative probability
D (μm² s⁻¹)
B
D (μm² s⁻¹)
Syn Con
Syn Glu+CNQX
ESy Con
ESy Glu+CNQX
C
Presynaptic
Extrasynaptic
Presynaptic + Glu +CNQX
Extrasynaptic + Glu +CNQX
MSD (μm²)
Time interval (s)
D
Presynaptic dwell time (s)
NS
Control
Glu
Glu+CNQX
E
Control
Glu
Glu + DMSO
Glu + BAPTA
Cumulative probability
Presynaptic D (μm² s⁻¹)
F
Presynaptic D (μm² s⁻¹)
NS
Control
Glu
Glu + DMSO
Glu + BAPTA

Figure S5

Figure S5. Effects of CNQX and BAPTA on glutamate-induced reduction in $GABA_BR$ mobility (related to Figures 4 and 5).

(A) Cumulative probability distributions of presynaptic and axonal-extrasynaptic R1aR2 diffusion coefficients in control and +30 µM glutamate (Glu) and 10 µM CNQX. (B) Box-plot showing the IQR and median *D* for presynaptic (Syn) and axonal-extrasynaptic (ESy) R1aR2 in control and +glutamate and CNQX. (C) MSDs for synaptic and axonal-extrasynaptic R1aR2 in control and +glutamate and CNQX. (D) Presynaptic dwell times in control and in the presence of glutamate or glutamate + CNQX. (E) Cumulative probability distributions for presynaptic $GABA_BR$ diffusion coefficients in control and +30 µM glutamate with (+Glu and DMSO) or without (+Glu) pre-treatment with vehicle or BAPTA-AM (+Glu and BAPTA). Neurons were pre-incubated in either vehicle or BAPTA-AM for 20 min at 37°C and imaged in 30 µM glutamate in the presence of vehicle or BAPTA-AM. (F) Box-plot showing the IQR and median diffusion coefficients for the data in A. *P<0.05, **P<0.01, ***P<0.001, NS – not significant; one-way ANOVA, KS test.

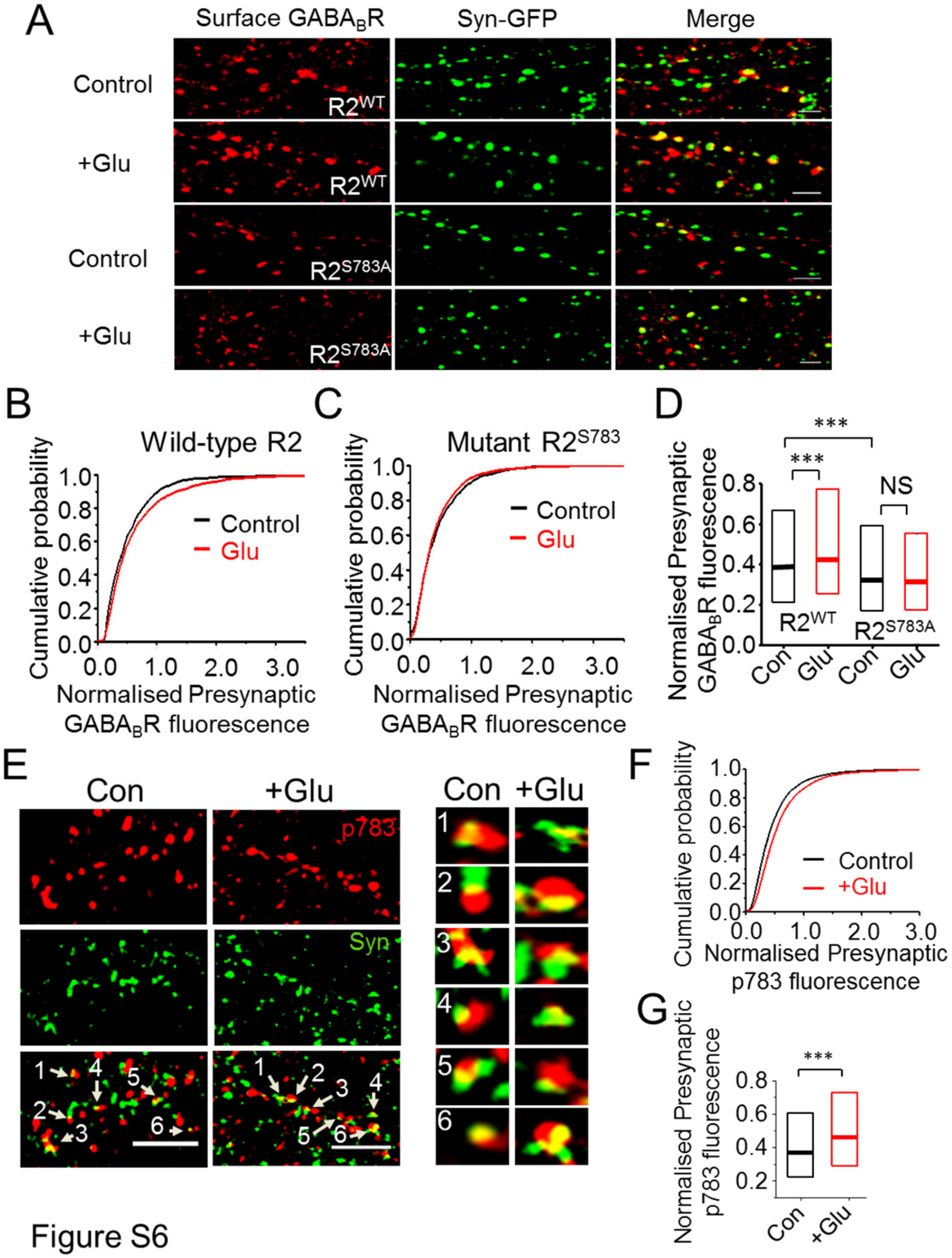
A
Surface GABABR
Syn-GFP
Merge
Control
+Glu
R2WT
R2S783A
B
Wild-type R2
Cumulative probability
Control
Glu
Normalised Presynaptic GABABR fluorescence
C
Mutant R2S783
D
***
NS
Normalised Presynaptic GABABR fluorescence
Con
Glu
E
Con
+Glu
p783
Syn
F
+Glu
Normalised Presynaptic p783 fluorescence
G
Normalised Presynaptic p783 fluorescence

Figure S6

Figure S6. Accumulation of cell surface $GABA_B$Rs at presynaptic terminals: effect of glutamate (related to Figure 6) and phosphorylation of $GABA_BR2^{S783}$ (related to Figure 7)

(A) Images showing co-localisation of cell-surface $GABA_B$R1a with synaptophysin-eGFP in hippocampal neurons at 14 *DIV*. Cells were transected at 7 *DIV* using cDNAs encoding for $R1a^{BBS}$ with either wild-type (WT) or mutant (S783A) R2, and synaptophysin-eGFP, and at 14 *DIV* incubated in control Krebs or 30 μM glutamate (+Glu) for 5 min at 37°C. Neurons were then fixed and incubated in 4 μg/ ml bungarotoxin Alexa Fluor 555 for 10 min at room temperature and imaged.. Scale bar = 2 μm. (B) Cumulative probability distribution for cell surface presynaptic $R1a^{BBS}$ fluorescence normalized to synaptophysin-eGFP for $GABA_B$Rs containing WT R2 in control and +Glu. (C) Cumulative probability distribution for cell surface presynaptic $R1a^{BBS}$ fluorescence normalized to synaptophysin-eGFP for mutant receptors containing $R2^{S783A}$ in control and +Glu. (D) IQR and median of normalized cell surface presynaptic fluorescence for WT (R1aR2) and mutant ($R1aR2^{S783A}$) receptors in control and +Glu. Normalised F_{median} WT control = 0.38 (n = 1094), F_{median} WT +Glu = 0.42 (n = 2174), F_{median} mutant control = 0.32 (n = 1444), F_{median} mutant +Glu = 0.31 (n = 1901). (E) Co-localization of $GABA_B$R2 p783 and synaptophysin (Syn) in hippocampal neurons in control Krebs or 30 μM glutamate (+Glu) for 5 min at 37°C. At 14 *DIV*, neurons were incubated in glutamate, fixed, permeabilised and labeled using primary antibodies against phospho-783 and synaptophysin. The right hand panel shows the zoomed up co-localization in the arrowheads. (F) Cumulative probabilities for presynaptic p783 $GABA_B$R2 fluorescence normalized to Syn fluorescence in control and +Glu. (G) Median values for normalized p783 $GABA_B$R2 presynaptic fluorescence in control and +Glu. ***P<0.001, KS test. Scale bar = 5 μm

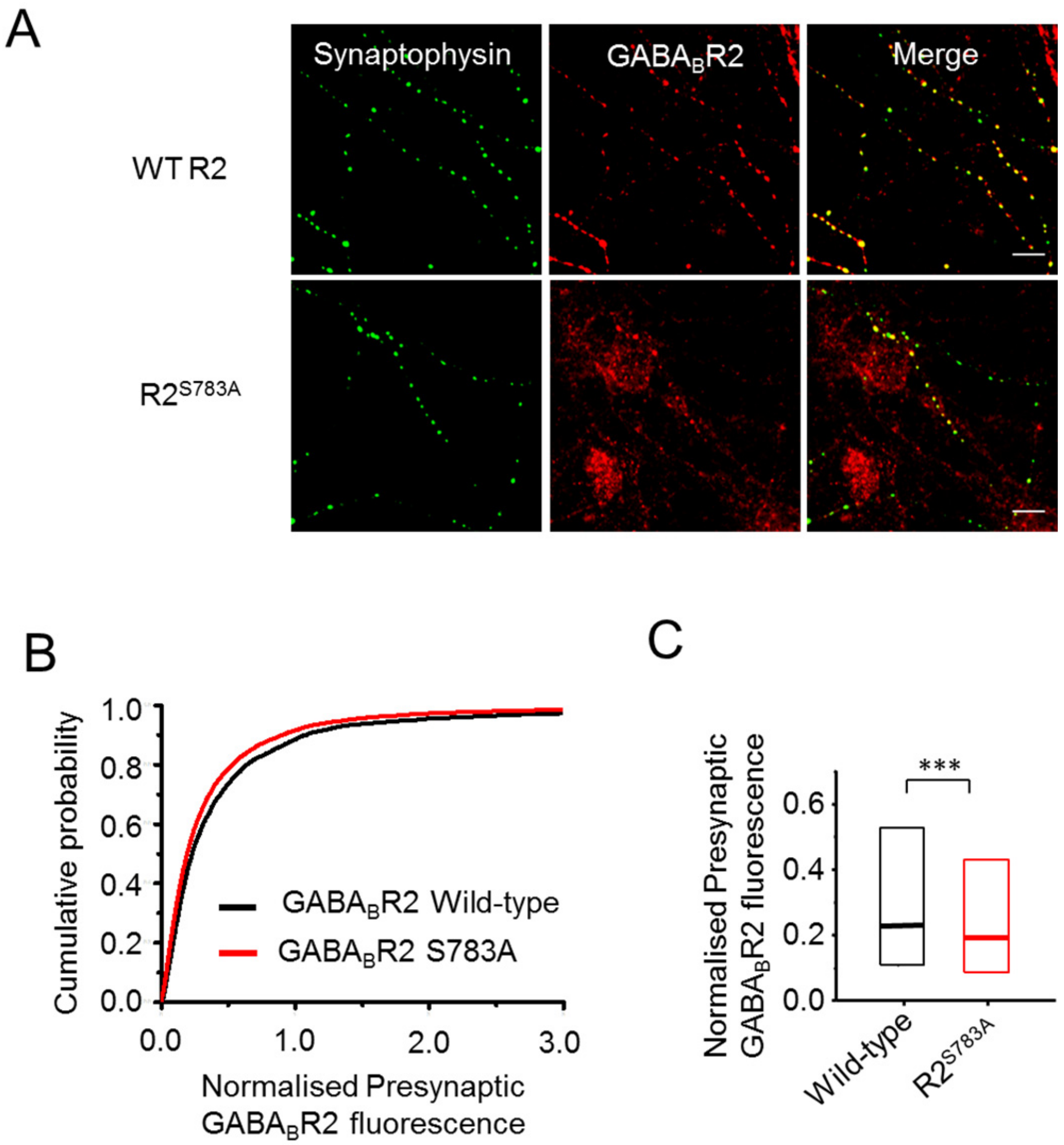

Figure S7. Reduced basal expression of GABA$_B$R2^{S783A} (related to Figure 7).

(A) Images showing co-localisation of WT GABA$_B$R2 and R2^{S783A} with synaptophysin in hippocampal neurons at 14 *DIV*. Cells were transected at 7 *DIV* using cDNAs encoding for R1a, with either WT or mutant R2, and with synaptophysin-eGFP, fixed at 14 *DIV,* permeabilised and labeled using primary antibodies against R2 and secondary antibodies conjugated to Alexa Fluor 594 before imaging. (B) Cumulative probability distribution for presynaptic GABA$_B$R2 fluorescence normalized to synaptophysin-eGFP for WT GABA$_B$R2 or GABA$_B$R2^{S783A}. (C) IQR and median of normalized GABA$_B$R2 presynaptic fluorescence for WT GABA$_B$R2 or GABA$_B$R2^{S783A}. F_{median} WT = 0.23 (n = 3127). F_{median} mutant = 0.19 (n = 6089). ***P<0.001 MW test. Scale bar = 5 μm.

Table S1. Median Diffusion Coefficients of $GABA_BRs$ (related to Figure 1-5, 7)

Receptor	Drug	Median *D* ($\mu m^2 s^{-1}$)	Number of Receptors	Number of Neurons
Cell surface receptors not localized to synapses				
R1aR2	Control	0.12	1529	32
R1aR2	Baclofen	0.10	3262	48
R1bR2	Control	0.07	804	22
R1bR2	Baclofen	0.09	3061	52
Cell surface receptors localized to presynaptic terminals				
R1aR2	Control	0.078	445	115
R1bR2	Control	0.108	276	86
$R1a^{\Delta SD1}R2$	Control	0.11	295	27
$R1a^{\Delta SD2}R2$	Control	0.081	574	47
$R1aR2^{S783A}$	Control	0.076	264	38
R1aR2	Baclofen	0.063	515	114
R1bR2	Baclofen	0.11	397	59
$R1a^{\Delta SD1}R2$	Baclofen	0.08	211	34
$R1a^{\Delta SD2}R2$	Baclofen	0.071	322	47
R1aR2	Glutamate	0.048	203	69
R1aR2	Glutamate + TTX	0.05	324	48
R1aR2	Glutamate + CNQX +APV	0.079	197	38
R1aR2	Glutamate + CNQX	0.043	205	23
R1aR2	NMDA Control	0.045	532	59
R1aR2	NMDA Glutamate Control	0.037	198	20
R1aR2	NMDA	0.019	234	40
R1aR2	NMDA Glutamate + APV	0.047	485	36
R1aR2	Glutamate+ BAPTA Vehicle	0.05	204	40
R1aR2	Glutamate + BAPTA	0.07	347	56
$R1aR2^{S783A}$	Glutamate	0.077	261	56
Cell surface receptors localized to axonal extrasynaptic membranes				
R1aR2	Control	0.124	1834	115
R1bR2	Control	0.129	1203	86
$R1a^{\Delta SD1}R2$	Control	0.11	532	27
$R1a^{\Delta SD2}R2$	Control	0.09	1202	47
R1aR2	Baclofen	0.075	2740	114
R1bR2	Baclofen	0.13	1814	59
R1aR2	Glutamate	0.105	865	69
R1aR2	Glutamate + TTX	0.095	2584	48
R1aR2	Glutamate + CNQX +APV	0.089	2722	38

Table shows Diffusion coefficients determined from the MSD plots (see Supplemental Experimental Procedures) for wild-type and mutant, BBS-tagged, $GABA_BR$ subunits taken from n receptors and n cells.

Supplementary Movie titles

Supplemental Movie 1 – Real-time lateral diffusion of Quantum Dot tagged $GABA_B$ receptors on neuronal membranes (related to Figure 1)

Supplemental Movie 2 - Real-time lateral diffusion of Quantum Dot tagged $GABA_B$ receptors on axonal membranes labeled with synaptophysin-eGFP (related to Figure 2)

Supplemental Movie 3 – Real-time lateral diffusion of Quantum Dot tagged $GABA_B$ receptors on presynaptic terminals labeled with synaptophysin-eGFP, along with axonal extrasynaptic and exchanging receptors (related to Figure 2)

Supplemental Experimental Procedures

Molecular Biology, Antibodies and Drugs – The $GABA_BR1$ isoforms ($R1a^{BBS}$, $R1b^{BBS}$) containing a bungarotoxin binding site, a flag-tagged $GABA_BR2$ (R2), $R1a^{BBS}$ containing a deletion of the N-terminal ($R1a^{\Delta SD1}$) or C-terminal ($R1a^{\Delta SD2}$) Sushi Domains, an R2 subunit with a single amino acid substitution from serine 783 to alanine (S783A), and pEGFP-C1, have all been described previously (Hannan et al., 2012; Hannan et al., 2011; Kuramoto et al., 2007). Synaptophysin-eGFP and Synaptophysin-GcAMP6Fast were generous gifts from Yukiko Goda (Riken Brain Science Institute) and Leon Lagnado (University Sussex), respectively.

Antigens were labeled in fixed neurons using mouse primary antibodies against synaptophysin (Abcam ab8049), PSD95 (Neuromab K28/43), gephyrin (Synaptic systems 147 111), a guinea pig antibody against $GABA_BR2$ (Chemicon ab5394), and a rabbit antibody against phospho783 (Santa Cruz sc-135695) followed by an goat anti-mouse secondary antibody conjugated to Alexa Fluor 488 or Alexa Fluor 555 (Molecular Probes) or goat anti-guinea pig Alexa-Fluor 594 (Molecular Probes) or goat anti-rabbit Alexa-Fluor 555 (Molecular Probes) antibody. All drugs and chemicals were obtained from Sigma unless specified otherwise.

Cell Culture and transfection – Dissociated hippocampal cultures were prepared from E18 Sprague-Dawley rat embryos as described previously (Hannan et al., 2011). Briefly this involved dissociation of dissected hippocampi into single cells followed by plating onto 18 or 22 mm glass coverslips (Assistence/ VWR) pre-coated with poly-D-lysine and immersed in a medium containing: minimum essential media (MEM; Life Technologies), supplemented with 5% v/v heat-inactivated fetal calf serum, 5% v/v heat-inactivated horse serum (Life Technologies), penicillin-G/ streptomycin (100 U/100 μg/ml), 2 mM glutamine, and 20 mM glucose. After 2 hrs, the media was replaced and the cells were maintained until used for experiments in a media containing Neurobasal-A (Life Technologies), supplemented with 1% v/v B-27, penicillin-G/ streptomycin (100 U/100 μg/ml), 0.5% v/v Glutamax (Life Technologies), and 35 mM glucose. Neurons were transfected at 7-8 days *in vitro* (DIV) using a calcium phosphate based method (Hannan et al., 2011).

Labeling BBS-containing $GABA_BRs$ with QDs - Hippocampal neurons expressing BBS-containing $GABA_BRs$ were incubated in 4 μg/ml biotinylated BgTx (BgTx-B; Life Technologies) for 2 min at 37°C. The cells were washed with Krebs (3x) and incubated in 10 pM Quantum Dot 655 conjugated to streptavidin (QD; Life Technologies) for 1 min at 37°C in QD binding buffer, as described previously (Levi et al., 2011). The buffer contained BSA (2% w/v), sodium azide (1 mM), sucrose (215 mM), and sodium borate (2.5 mM). The cells were washed thoroughly in Krebs and mounted in a recording chamber at 37°C (Solent Scientific) for live cell imaging in Krebs or in Krebs containing vehicle or drugs. All real-time imaging was carried within five min of drug application.

Real-time imaging of single $GABA_BRs$ - A wide-field imaging setup was used to locate QD labeling in neurons and to image $GABA_BR$-QD complexes for single particle tracking. The imaging hardware comprised an Olympus IX71 inverted microscope, with a 60X objective (NA – 1.35; Olympus) and halogen lamp illumination (PhotoFluor-II Metal Halide illumination system). Images were acquired using a back-illuminated cooled electron-multiplying charge coupled device (EMCCD) camera (iXon3 885; Andor Technology). eGFP was imaged with a 457 - 487 nm (Semrock) band-pass excitation filter, a 496 nm long-pass emission filter, and 495 nm

dichroic mirror. QD655 was imaged with a 415 - 455 nm band-pass excitation filter, a 647.5 - 662.5 nm band-pass emission filter, and a 510 nm dichroic mirror. Single images of QD specificity were acquired with optimal exposure (typically 30 - 150 ms) in 16 bits using Cairn-Metamorph Meta Imaging software (Molecular Devices; version 7.7.10) and stored for analysis.

For imaging the real-time movement of single $GABA_BRs$, a suitable region-of-interest (ROI) was selected and an image taken of the eGFP expressing area (typical exposure time ~30 - 150 ms based on levels of eGFP expression). Next, the filters were changed to image QDs without changing the field of view and an image sequence of 300 frames captured at 33 Hz would be taken and stored for later tracking analysis.

Single particle tracking - For $GABA_BRs$, this was undertaken using previously described methods (Bannai et al., 2009; Dahan et al., 2003; Levi et al., 2011; Renner et al., 2009). Single $GABA_BRs$ coupled to QD655 via the BBS were identified by their characteristic 'blinking' (Dahan et al., 2003). Matlab (MathWorks) based customised software, SPTrack (Ver5), was used to track the QDs. For every image, in sequence, the centre of the QD spot fluorescence was determined by a 2-D Gaussian fit with a spatial resolution of ~10 – 20 nm. This was undertaken for all QDs. The Gaussian peaks in a given frame were next associated with the Gaussian peaks from the previous frame based on estimated diffusion coefficients and the likelihood of the two Gaussian peaks in consecutive frames belonging to the same QD track. QDs which appear in at least 15 consecutive frames were used for tracking analysis and the shorter ones discarded. The mean square displacement (MSD) of each QD was calculated using the following equation:

$$\mathrm{MSD}\,(ndt) = (N - n)^{-1} \,.\, \Sigma_{\,i\,=\,1}^{\;N\,-\,n}((x_{i\,+\,n} - x_i)^2 + ((y_{i\,+\,n} - y_i)^2)$$

where x_i and y_i are the spatial co-ordinates of a QD on any image frame i, N is the total number of points in the trajectory, dt is the time interval between two successive frames (33 ms), and ndt is the time interval over which the displacement is averaged. From the MSD plot, the diffusion coefficient, *D, for a QD* was calculated by fitting the first two to five points of the MSD plot against time with the following equation:

$$\mathrm{MSD}(t) = 4D_{2\text{–}5}\, t + 4\sigma_x^{\,2}$$

where σ_x is the QD localization accuracy in one direction. D was determined from the slope of the relationship. Given the inherent noise in CCD imaging systems and the errors in precise pointing accuracy that results, trajectories with $D < 10^{-4}$ μm^2/ s were considered immobile.

Synaptic terminals were identified and thresholds set using a multidimensional image analysis plug-in based on MetaMorph (Molecular Devices) software (Racine et al., 2007) or using ImageJ. QD trajectories that co-localised with synaptic markers were defined as synaptic. QD data were subsequently analysed using Origin (Ver 6), Matlab, and SPSS (IBM).

Cell surface labeling - Hippocampal neurons expressing BBS-containing $GABA_BRs$ were fixed in 4% PFA for 10 min and after washing with Krebs (x 3) incubated in 4 μg/ml BgTx Alexa fluor 555 (Life Technologies) for 10 min at room temperature. The cells were washed with Krebs (3x) and mounted for imaging using confocal microscopy.

Confocal Imaging - Confocal images of synaptic protein expression and $GABA_BR$ presynaptic localisation were imaged using an upright Zeiss LSM 510 or Leica SP8 vis confocal microscopes. Images were acquired with optimum gain and offset in 8-bit using a 488 nm laser and 505 - 530 nm band-pass filter for eGFP/Alexa Fluor 488 and a 543 nm laser and 560 nm long-pass filter for Alexa Fluor 555. Confocal images were analysed in ImageJ. To quantify presynaptic $GABA_BR$ and phospho783 labelling levels, images from the synaptophysin channel were thresholded and the built-in 'Analyze Particles' function was used to detect and add ROIs to the ROI manager. Next, the ROIs were transferred to the $GABA_BR$ channel and the fluorescence intensity of $GABA_BR$ staining within the synaptophysin ROIs were measured and normalized to the fluorescence intensity from the raw synaptophysin channel. The cumulative probability of the distribution for normalized $GABA_BR$ intensity was used for analysis. For analyzing intracellular fluorescence, longitudinal ROIs were drawn on dendrites identified using eGFP expression and transferred to images on the corresponding $GABA_BR$ channels to measure the $GABA_BR$ fluorescence.

Calcium imaging and analysis – Synaptophysin-GCaMP6 Ca^{2+} transients were image captured in the presence of a brief (<3 min) application of 30 μM NMDA and 10 μM D-serine using the wide-field setup described above. Images of presynaptic terminals were acquired for 15 s at 20 Hz. ROIs were drawn around presynaptic terminals and the fluorescence intensity within individual puncta were measured during the 15 s image sequence using ImageJ. The fluorescence signal (F) was normalised to baseline fluorescence (F_0) to obtain values of $\Delta F/F_0$. $\Delta F/F_0$ peaks were detected and their amplitudes measured in Matlab using the plug-in Peakfinder (Nathanael Yoder, Mathworks). The maximum normalised peak from each presynaptic terminal during the 15 s imaging period and the average normalised Ca^{2+} peak of each terminal have been used for analysis. Ca^{2+} transients less than 3x the signal-to-noise ratio were excluded from the analysis.

Whole-cell patch-clamp recording – G-protein-coupled inwardly-rectifying K^+ (Kir) channel currents elicited in response to $GABA_BR$ activation by 100 μM baclofen, were recorded from hippocampal neurons in culture at 9, 12 or 14 *DIV*, using patch clamp electrophysiology. Recordings were performed on: untransfected neurons or those transfected at 7 *DIV* with eGFP only or with eGFP, $R1a^{BBS}$ or $R1b^{BBS}$ and R2. Transfected cells were identified by eGFP expression. Patch pipettes (4-5 MΩ) fabricated from thin-walled borosilicate glass (GC-150TF-10; Harvard Apparatus, Kent, UK), were filled with internal solution (mM: 120 KCl, 2 $MgCl_2$, 11 EGTA, 30 KOH, 10 HEPES, 1 $CaCl_2$, 1 GTP, 2 ATP, pH 7.4). Neurons were continuously perfused with a Krebs solution containing (mM): 140 NaCl, 2.5 $CaCl_2$, 4.7 KCl, 1.2 $MgCl_2$, 11 glucose, and 5 HEPES, pH 7.4 and voltage-clamped at -70 mV in the whole-cell configuration, with series resistance compensation (~40%) applied. Membrane currents consecutively generated by 20 sweeps of -10 mV hyperpolarising steps were recorded with an Axopatch 200B amplifier (Molecular Devices, California, USA) at 10 kHz filtering. Currents were recorded using repeat trains of sweeps. Data were discarded if series resistance changed by > 10%. To increase the size of GIRK currents and to convert them to inward currents whilst holding at - 70 mV, prior to baclofen application, the KCl concentration was increased to 25 mM and the NaCl concentration reduced to 120 mM in the Krebs solution. This changed E_K from approximately -92 mV to -47 mV. In addition, 2 mM kynurenic acid and 100 μM picrotoxin were added to the Krebs to block ionotropic glutamate and $GABA_A$ receptors. Peak K^+ current amplitudes were filtered at 5 kHz before storage for analysis with Clampex 10 software. Whole-cell capacitance

was calculated using WinWCP V4.7 software (J Dempster, Strathclyde University) from the area under the membrane current discharge curve elicited by a series of -10 mV hyperpolarizing pulses before capacity compensation was applied. Current density was calculated by dividing the peak amplitude by the whole-cell capacitance.

Supplemental references

Levi,S., Dahan,M., and Triller,A. (2011). Labeling neuronal membrane receptors with quantum dots. Cold Spring Harb. Protoc. *2011*, rot5580.

Racine,V., Sachse,M., Salamero,J., Fraisier,V., Trubuil,A., and Sibarita,J.B. (2007). Visualization and quantification of vesicle trafficking on a three-dimensional cytoskeleton network in living cells. J. Microsc. *225*, 214-228.
