# Supplementary figures and images for: Phospho-dependent Accumulation of GABABRs at Presynaptic Terminals after NMDAR Activation
Source: Cell Rep. 2016 Aug 4;16(7):1962–73. doi: 10.1016/j.celrep.2016.07.021 (PMC4987283; doi:10.1016/j.celrep.2016.07.021)

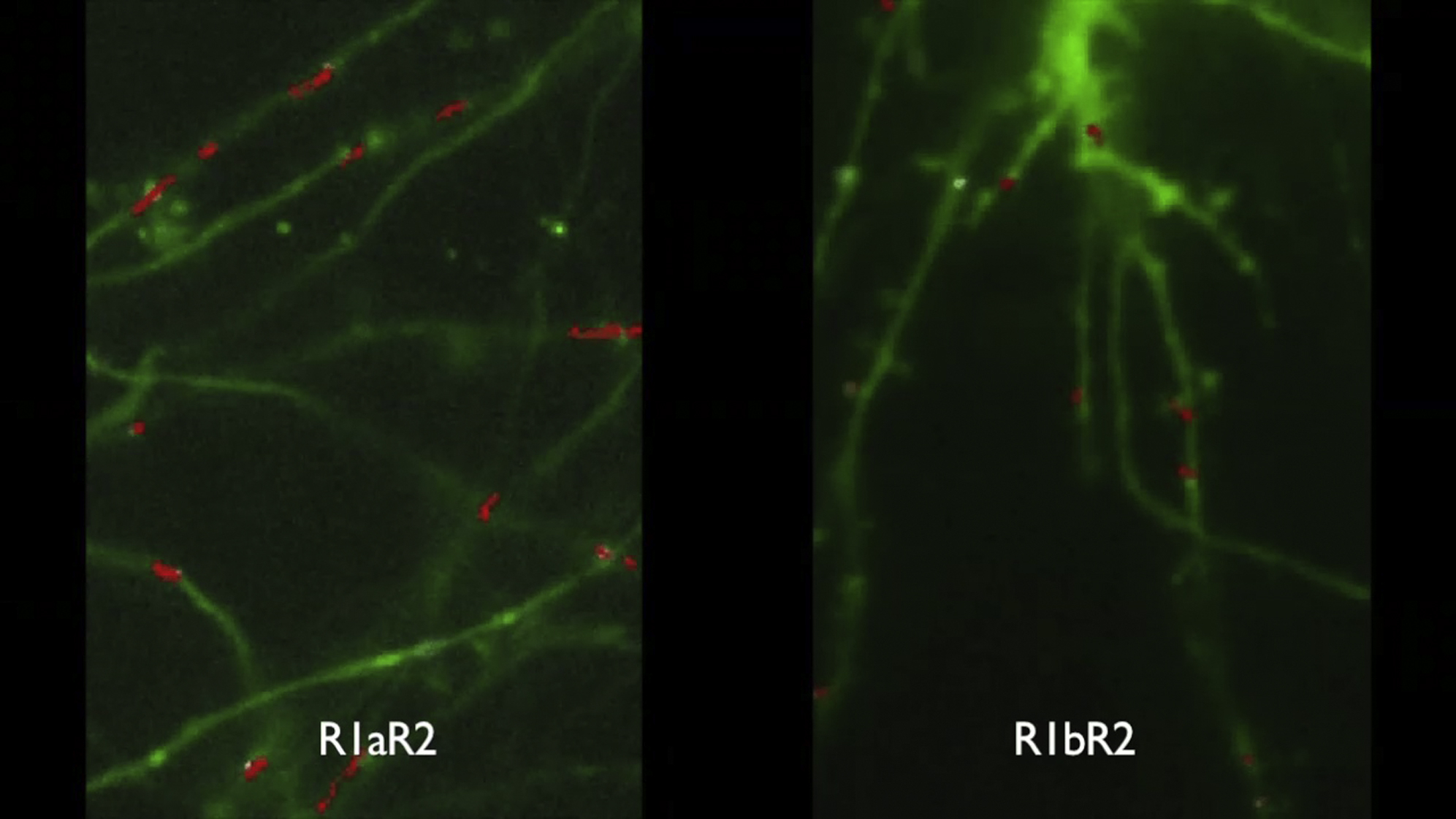

Supplement: Movie S1. Real-Time Lateral Diffusion of Quantum-Dot-Tagged GABAB Receptors on Neuronal Membranes, Related to Figure 1 [file mmc2.jpg]

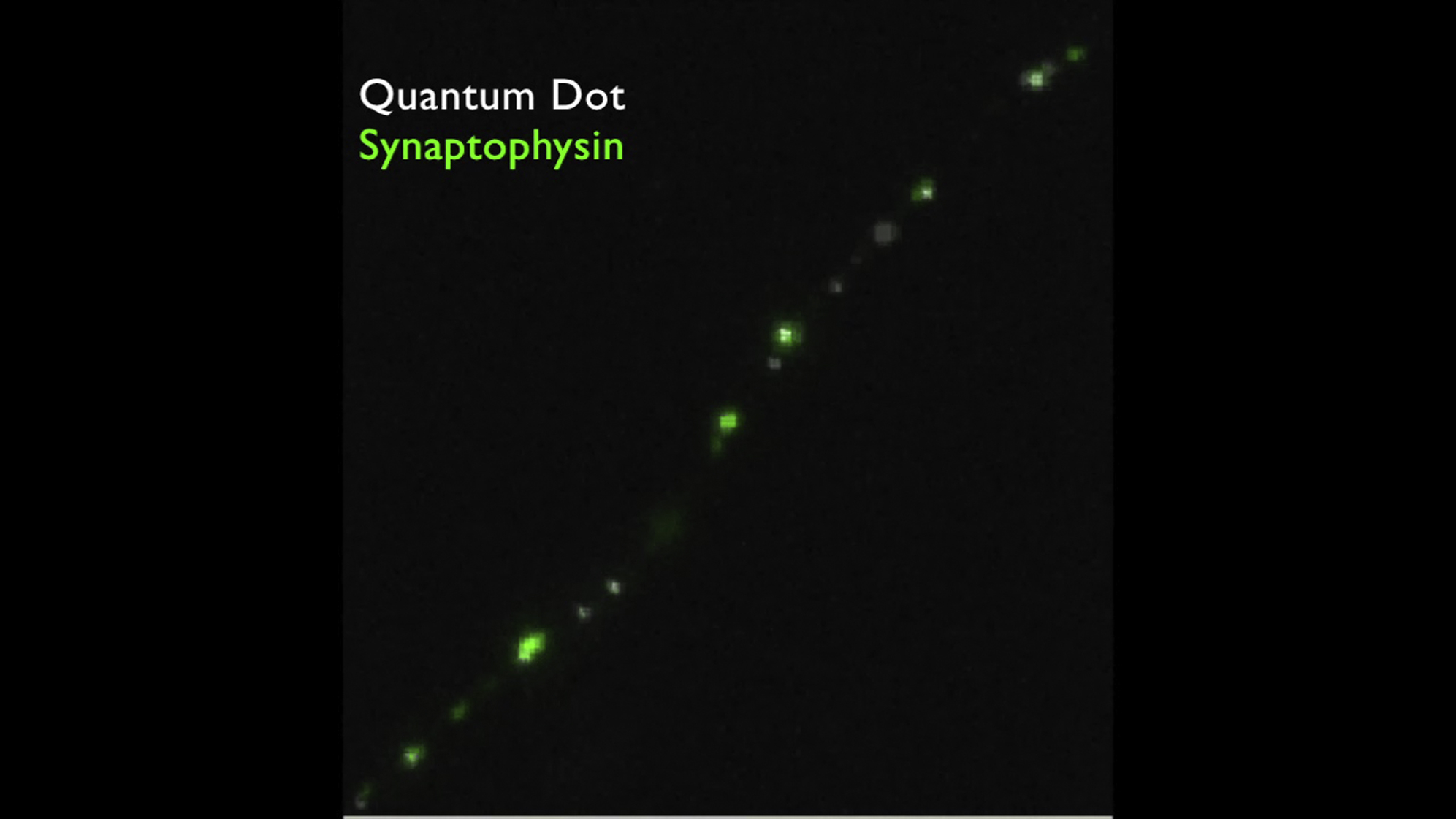

Supplement: Movie S2. Real-Time Lateral Diffusion of Quantum-Dot-Tagged GABAB Receptors on Axonal Membranes Labeled with Synaptophysin-eGFP, Related to Figure 2 [file mmc3.jpg]

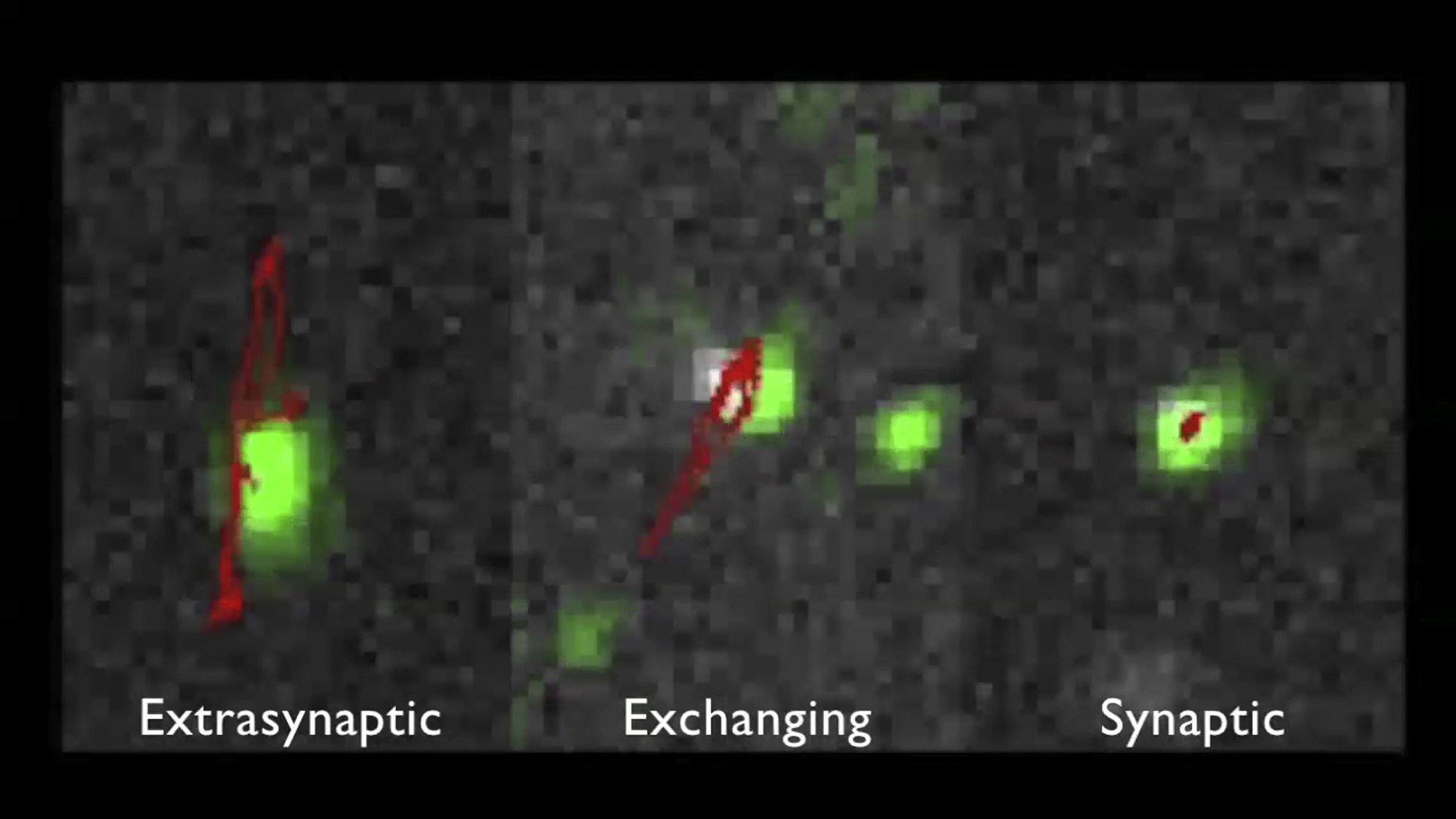

Supplement: Movie S3. Real-Time Lateral Diffusion of Quantum-Dot-Tagged GABAB Receptors on Presynaptic Terminals Labeled with Synaptophysin-eGFP along with Axonal Extrasynaptic and Exchanging Receptors, Related to Figure 2 [file mmc4.jpg]
