# Supplementary material for: Phospho-dependent Accumulation of GABABRs at Presynaptic Terminals after NMDAR Activation
Source: Cell Rep. 2016 Aug 4;16(7):1962–73. doi: 10.1016/j.celrep.2016.07.021 (PMC4987283; doi:10.1016/j.celrep.2016.07.021)
Supplement: Document S2. Article plus Supplemental Information [file mmc5.pdf]

Cell Reports

Article

Phospho-dependent Accumulation of $GABA_B$Rs at Presynaptic Terminals after NMDAR Activation

Graphical Abstract

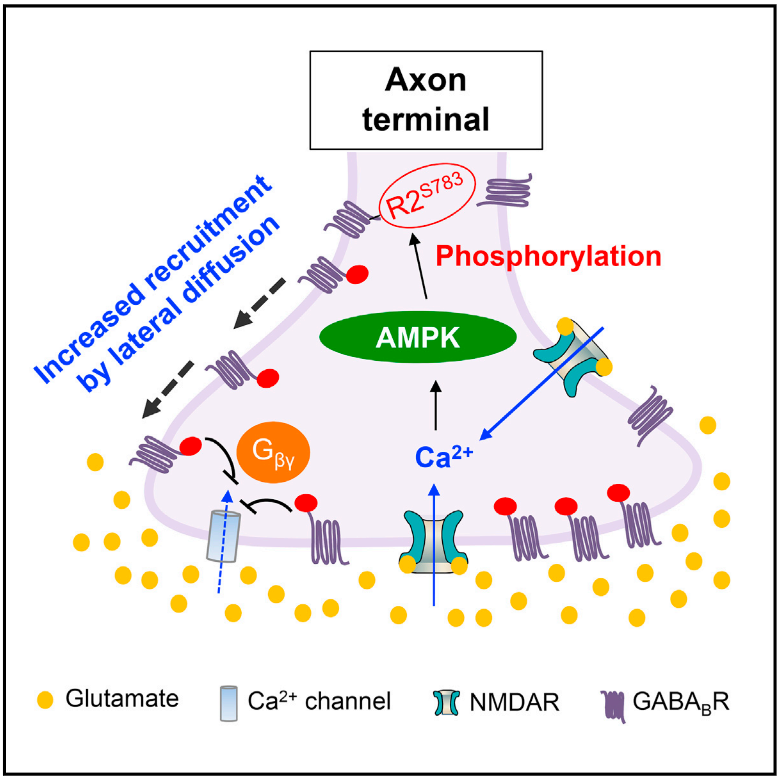

Authors

Saad Hannan, Kim Gerrow, Antoine Triller, Trevor G. Smart

Correspondence

t.smart@ucl.ac.uk

In Brief

Hannan et al. report a GABA-mediated inhibitory homeostatic mechanism by which lateral diffusion of presynaptic $GABA_B$ receptors and their accumulation on presynaptic excitatory terminals causes a reduction of presynaptic activity initiated by NMDA receptor activation. The underlying mechanism requires elevated intracellular Ca^{2+} and AMPK-dependent phosphorylation of presynaptic $GABA_B$ receptor subunits on serine 783.

Highlights

- Presynaptic $GABA_B$Rs are recruited to axon terminals by lateral diffusion
- Activated $GABA_B$R1aR2 complexes become less mobile due to their sushi domains
- NMDAR activity recruits $GABA_B$Rs to axon terminals by slowing their mobility
- $GABA_B$R accumulation depends on intracellular Ca^{2+} and phosphorylation of $GABA_BR2^{S783}$

Hannan et al., 2016, Cell Reports *16*, 1962–1973
August 16, 2016 © 2016 The Author(s).
http://dx.doi.org/10.1016/j.celrep.2016.07.021

OPEN ACCESS
CellPress

Cell Reports
Article

Phospho-dependent Accumulation of $GABA_BRs$ at Presynaptic Terminals after NMDAR Activation

Saad Hannan,[1] Kim Gerrow,[2] Antoine Triller,[2] and Trevor G. Smart[1,*]
[1]Department of Neuroscience, Physiology and Pharmacology, University College London, Gower Street, London WC1E 6BT, UK
[2]Biologie Cellulaire de la Synapse, Inserm U1024, Institute of Biology, École Normale Supérieure (ENS), 46 rue d'Ulm, Paris 75005, France
*Correspondence: t.smart@ucl.ac.uk
http://dx.doi.org/10.1016/j.celrep.2016.07.021

SUMMARY

Here, we uncover a mechanism for regulating the number of active presynaptic $GABA_B$ receptors ($GABA_BRs$) at nerve terminals, an important determinant of neurotransmitter release. We find that $GABA_BRs$ gain access to axon terminals by lateral diffusion in the membrane. Their relative accumulation is dependent upon agonist activation and the presence of the two distinct sushi domains that are found only in alternatively spliced $GABA_BR1a$ subunits. Following brief activation of NMDA receptors (NMDARs) using glutamate, $GABA_BR$ diffusion is reduced, causing accumulation at presynaptic terminals in a Ca^{2+}-dependent manner that involves phosphorylation of $GABA_BR2$ subunits at Ser783. This signaling cascade indicates how synaptically released glutamate can initiate, via a feedback mechanism, increased levels of presynaptic $GABA_BRs$ that limit further glutamate release and excitotoxicity.

INTRODUCTION

Maintaining spatio-temporal stability over neural network activity is important for brain function (Davis, 2006; Marder and Goaillard, 2006; Turrigiano, 1999). In particular, exercising tight control over excitatory transmission is important to avoid the consequences of excessive glutamate-mediated signaling, characterized by ischemic insults, traumatic brain injury, and epilepsy (Choi, 1987; Meldrum, 1994; Werner and Engelhard, 2007).

Several mechanisms have evolved to limit excessive neurotransmission, involving rapid changes to glutamate receptor conformation (e.g., desensitization) and reductions in synaptic glutamate concentration (e.g., uptake), to longer latency silencing of excitatory synapses, withdrawal of dendritic spines, and ablation of neurons (Paoletti et al., 2013; Traynelis et al., 2010). In addition, GABA, the principal inhibitory transmitter in the brain, activates signaling pathways that regulate cell excitability via ionotropic ($GABA_A$ receptors [$GABA_ARs$]; Fritschy and Panzanelli, 2014; Moss and Smart, 2001) and metabotropic GABA receptors ($GABA_BRs$; Benke et al., 2015; Bettler and Tiao, 2006). Importantly, altering GABA receptor numbers at inhibitory synapses can indirectly regulate excitation, exemplified by the dispersal of $GABA_ARs$ following NMDA receptor (NMDAR) activation (Muir et al., 2010).

Although increasing postsynaptic inhibition is effective at reducing excitability, targeting presynaptic terminals enables a precise input-selective approach by regulating neurotransmitter release. The close proximity of $GABA_BRs$ to excitatory synapses makes them ideal candidates in this regard. Presynaptic $GABA_BRs$ can inhibit voltage-gated Ca^{2+} channels (Bettler et al., 2004), and when activated by GABA spillover from nearby inhibitory synapses, these high-affinity receptors can inhibit glutamate release.

Prolonged NMDAR activation can also regulate $GABA_BR$ trafficking by increasing their internalization and degradation (Benke et al., 2012; Maier et al., 2010). This reduction in surface $GABA_BR$ numbers was unexpected and raised the question as to what homeostatic mechanisms exist to ensure the long-term stability of excitatory transmission, without ensuing excitotoxicity. For example, could inhibition be differentially affected by physiological activation of NMDARs at excitatory synapses? Furthermore, can mobile cell surface inhibitory receptors regulate synaptic transmission (Choquet and Triller, 2013)? In this context, diffusing presynaptic $GABA_BRs$ could limit the release of glutamate, especially during ischemia (Cimarosti et al., 2009) and excitotoxicity (Benke, 2013). Although the lateral mobility of several receptors in the postsynaptic density has been characterized (Bürli et al., 2010; Choquet and Triller, 2013; Fernandes et al., 2010; Jaskolski and Henley, 2009; Ladépêche et al., 2014), little is known about the presynaptic lateral mobility of $GABA_BRs$ around axonal membrane compartments (Ladepeche et al., 2013; Mikasova et al., 2008).

Here, we describe how activated $GABA_BRs$ rapidly accumulate at presynaptic terminals by lateral diffusion, a feature that relies on their sushi domains (SDs). Brief NMDAR activation promotes $GABA_BR$ accumulation at presynaptic terminals after phosphorylation of the $GABA_BR2$ subunits. Controlling presynaptic $GABA_BR$ mobility can thus form a basis for homeostatic regulation of excitatory transmission.

RESULTS

Lateral Diffusion of $GABA_BRs$ on Hippocampal Neurons

To determine whether single $GABA_BRs$ can diffuse to discrete synaptic membrane domains, we labeled them with quantum

 © 2016 The Author(s).
This is an open access article under the CC BY license (http://creativecommons.org/licenses/by/4.0/).

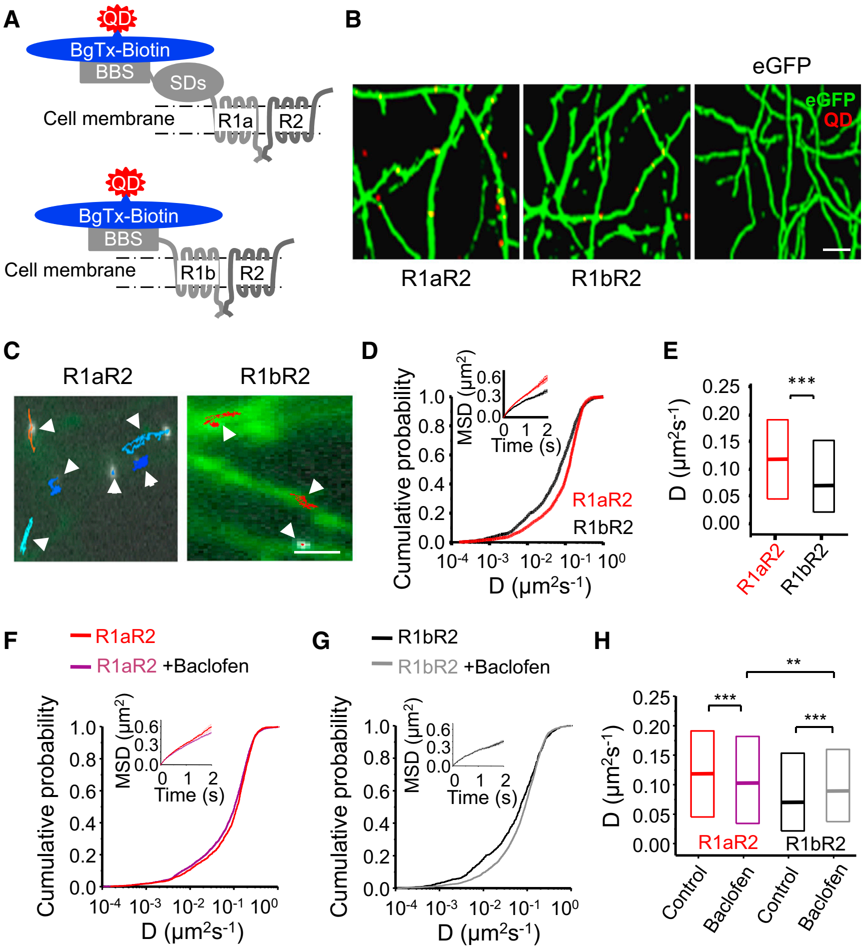

Figure 1. Single $GABA_BRs$ Are Mobile on Hippocampal Cell Surface Membranes

(A) $GABA_BR$ subunits with BBS, bound BgTx-biotin (BgTx-B), and QD655 coupled to streptavidin. Note sushi domains (SDs) in R1a.

(B) Specific QD labeling occurs for cells expressing $R1a^{BBS}$ (R1a) or $R1b^{BBS}$ (R1b) with R2 and not for eGFP controls incubated in BgTx-B (4 μg/ ml; 2 min) and then QD (10 pM for 1 min at 37°C). The scale bar represents 5 μm.

(C) QD trajectories (arrowheads) for single R1aR2 and R1bR2. The scale bar represents 2 μm.

(D) Cumulative probabilities for diffusion coefficients, *D*, and mean square displacements (MSDs) (inset) for R1aR2 and R1bR2.

(E) Box plot of the 25%–75% inter-quartile range (IQR) and median *D* values.

(F) Cumulative probability distributions of R1aR2 *D* in control and +100 μM baclofen. Inset shows the MSD plots.

(G) Cumulative probabilities for R1bR2 *D* in control and +baclofen.

(H) Median *D* and IQR in control and +baclofen for the data in (F) and (G).

**$p < 0.01$; ***$p < 0.001$, KS test (see also Figures S1 and S2 and Movie S1).

dots (QDs) by adding a "functionally silent" mimotope of the α-bungarotoxin (α-BgTx) binding site (BBS) to the start of the N-terminal domain of individual $GABA_BR$ subunits (Figure 1A). This allowed their visualization on the surface of hippocampal neurons.

The two isoforms of $GABA_BR1$ (R1a and R1b) differ by two SDs in the N terminus of R1a, which are absent in R1b (Bettler and Tiao, 2006). Neurons expressing receptors with the BBS (R^{BBS}) were labeled using biotinylated α-BgTx (α-BgTx-B), to which QD655-streptavidin (QD) can subsequently bind (Figure 1A). This reaction labeled both R1 subunits to equal extent and was highly specific as neurons transfected with cDNAs encoding for either eGFP or wild-type $GABA_BRs$ (lacking a BBS) failed to bind QDs; moreover, incubating neurons with biotin-free α-Bgtx also failed to bind QD655 (<1% of control; Figures 1B and S1), thereby validating the use of the BBS for real-time labeling of $GABA_BRs$ with QDs.

The extent of recombinant $GABA_BR$ expression in transfected neurons was assessed from K^+ currents evoked by 10 or 100 μM baclofen. No difference in current density was observed between neurons expressing $GABA_BR1a^{BBS}R2$ with untransfected or eGFP-only controls at 2, 5, and 7 days post-transfection ($p > 0.05$; one-way ANOVA; Figures S2A and S2B), indicating there is no functional overexpression of cell surface $GABA_BRs$ coupled to inwardly rectifying K^+ (Kir) channels, even though levels of intracellular receptor were higher in $GABA_BR1a^{BBS}R2$-expressing neurons ($p < 0.05$; Figure S2C). This lack of functional overexpression could also reflect a limited supply of G proteins and/or Kir channels. However, beyond 7 days post-transfection, a trend toward increased baclofen-activated K^+ currents is observed in transfected cells compared to controls. As our studies are conducted before this time point, any receptor overexpression would not confound the results. Furthermore, a similar time profile for $GABA_BR$ expression in neurons was obtained with $R1b^{BBS}R2$ (Figures S2A and S2B).

Labeling of cell surface $GABA_BR1a^{BBS}R2$ and $R1b^{BBS}R2$ (termed R1aR2 and R1bR2) expressed in cultured hippocampal neurons revealed lateral mobilities with a range of diffusion coefficients and confinement properties. R1aR2 were more mobile, traversing longer and less-confined tracks compared to the compact trajectories of R1bR2s (Figure 1C; Movie S1). Consistent with these profiles, R1aR2 display higher median diffusion coefficients (*D*; Table S1; $p < 0.001$, Kolmogorov-Smirnov [KS] test; Figures 1D and 1E) and are less confined (increased mean square displacement [MSD] plots; Figure 1D, inset) compared to R1bR2. Thus, under basal conditions, these receptors exhibit distinct diffusion profiles, highlighting an important role for the SDs present only in R1a.

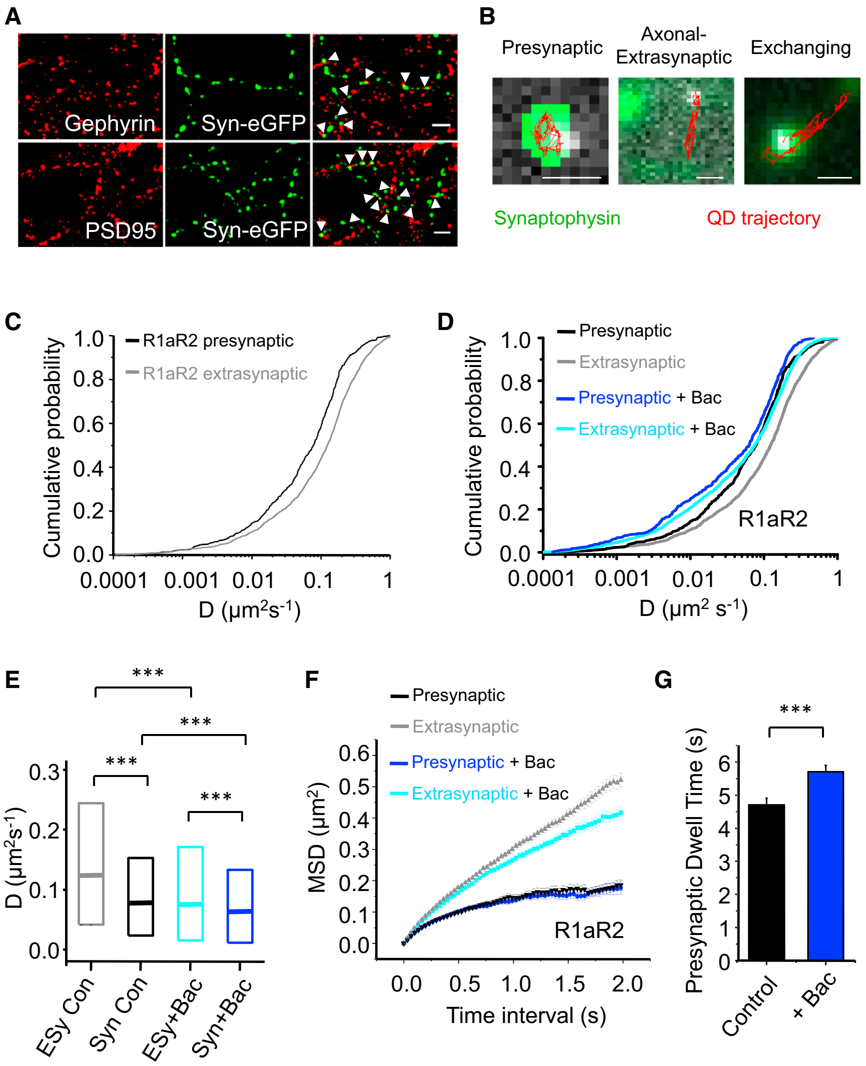

Figure 2. Recruiting $GABA_B$Rs into Presynaptic Terminals by Diffusion

(A) Cells showing close apposition of expressed synaptophysin (Syn)-eGFP puncta with endogenous gephyrin and PSD-95 (arrowheads). The scale bar represents 5 μm.

(B) Trajectories (red) of presynaptic, axonal-extrasynaptic, and exchanging QD-labeled R1aR2s. The scale bar represents 1 μm.

(C) Cumulative probability distributions for presynaptic and axonal-extrasynaptic R1aR2 diffusion coefficients.

(D) Cumulative probabilities for presynaptic and axonal-extrasynaptic *D* in control and +100 μM baclofen (Bac).

(E) Median *D* and IQR for presynaptic (Syn) and axonal-extrasynaptic (ESy) R1aR2s in control and +baclofen; ***$p < 0.001$, KS test.

(F) MSD plots for presynaptic and axonal-extrasynaptic R1aR2s in control and +baclofen.

(G) Presynaptic dwell times for R1aR2s in control and +baclofen (n = 245 receptors; ***$p < 0.001$, two-tailed unpaired t test).

Data in all bar charts are means ± SEMs (see also Figure S2 and Movies S2 and S3).

Activation Moderates the Lateral Mobility of $GABA_B$Rs

Activated receptors can be rapidly removed from synapses and their entry restricted (Borgdorff and Choquet, 2002; Groc et al., 2004; Mikasova et al., 2008) to prevent excessive signaling. Chronic activation of recombinant R1bR2s with baclofen (100 μM for 1 hr) increased their lateral mobility (Pooler and McIlhinney, 2007), but to examine whether near-maximal $GABA_B$R activation (Hannan et al., 2012) affects lateral mobility under physiological conditions, we studied hippocampal neurons exposed to 100 μM baclofen (chosen to maximally activate $GABA_B$Rs) for <5 min. Activated R1aR2 traversed the cell surface more slowly than non-activated controls (Table S1; $p < 0.001$, KS test; Figures 1F and 1H). By contrast, activated R1bR2 receptors exhibited higher *D* values compared to non-activated counterparts ($p < 0.001$, KS test; Figures 1G and 1H). Despite affecting diffusion, baclofen did not alter the confinement of either R1aR2 ($p > 0.05$; Figure 1F, inset) or R1bR2 (Figure 1G, inset) compared to controls, with R1aR2 remaining less confined. Even though baclofen slowed R1aR2 and increased mobility of R1bR2, the median *D* in baclofen for R1aR2 was still higher than that for R1bR2 ($p < 0.01$, KS; Figure 1H). Thus, the mobility of these receptors is differentially regulated by activation.

$GABA_B$Rs Are Recruited to Presynaptic Terminals by Lateral Diffusion

Studying the lateral mobility of receptors over the entire cell surface, without discrimination, obscures membrane-domain-specific effects. R1aR2 and R1bR2 are known to play different roles in synaptic transmission (Gassmann et al., 2004; Guetg et al., 2009; Pérez-Garci et al., 2006), with R1aR2 the predominant presynaptic isoform and R1bR2 found mostly postsynaptically near excitatory synapses.

To resolve membrane-domain-specific differences in receptor mobility, we studied $GABA_B$Rs in presynaptic compartments with synaptophysin-eGFP (Syn-eGFP) (Tarsa and Goda, 2002). Syn-eGFP clusters formed predominantly in axons close to markers for excitatory (PSD-95) and inhibitory postsynaptic (gephyrin) structures (Figures 2A and S2D). $GABA_B$Rs do not constitutively internalize into axons (Vargas et al., 2008), and therefore, lateral mobility would provide an important means of regulating their numbers at presynaptic terminals. For Syn-eGFP-positive axons and presynaptic terminals, QD-labeled R1aR2 clearly explored the surface by lateral diffusion (Movie S2). Receptors that frequented areas of axons that lacked Syn-eGFP are defined as *axonal-extrasynaptic*, a smaller fraction confined within Syn-eGFP clusters is classed as *presynaptic*, whereas the remainder transferred between these areas are defined as

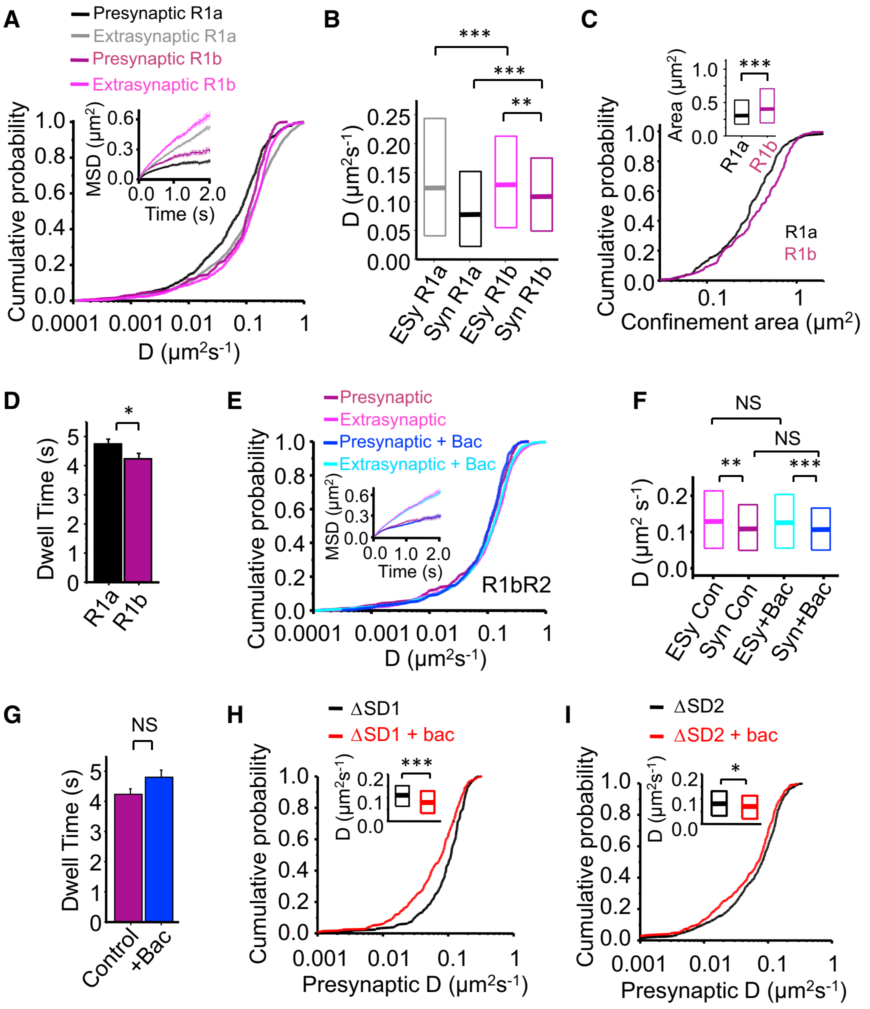

Figure 3. Diffusion Profiles for R1aR2s and R1bR2s at Presynaptic Terminals

(A) Cumulative probabilities for *D* and MSD plots (inset) for presynaptic and axonal-extrasynaptic R1aR2s (R1a) and R1bR2s (R1b).

(B) Median *D* and IQR for presynaptic (Syn) and axonal-extrasynaptic (ESy) receptors.

(C) Cumulative probabilities (inset—median and IQR) for presynaptic confinement areas for R1aR2s (n = 185 receptors) and R1bR2s (n = 351 receptors).

(D) Presynaptic dwell times for R1aR2s and R1bR2s.

(E) Cumulative probabilities and MSDs (inset) for control presynaptic and axonal-extrasynaptic R1bR2s and +100 μM baclofen (Bac).

(F) Median *D* and IQR for Syn and ESy R1bR2s in control and +baclofen.

(G) Presynaptic dwell time of control R1bR2s and +baclofen (n = 229).

(H) Cumulative probabilities for *D* (insets in H and I show median *D*) for presynaptic R1aR2s with the first sushi domain (ΔSD1) deleted in control and +100 μM baclofen.

(I) Cumulative probabilities for *D* for presynaptic R1aR2 with the second sushi domain (ΔSD2) deleted in control and +100 μM baclofen.

NS, not significant; *p < 0.05; **p < 0.01; ***p < 0.001, KS test, two-tailed unpaired t test (see also Figure S3).

exchanging (Figure 2B; Movie S3). Axonal-extrasynaptic R1aR2s were more mobile (higher *D*) and less confined compared to presynaptic R1aR2s ($p < 0.001$, KS test; Figures 2C, 2E, and 2F). The large variations in the confinement of R1aR2s, evident from MSD plots (Figure 2F), may reflect transient interactions with anchoring proteins at presynaptic terminals. These results imply that R1aR2s are recruited to presynaptic terminals by diffusion (Movies S2 and S3).

Activation Slows the Mobility of GABA$_B$Rs at Presynaptic Terminals

As the overall mobility of R1aR2 was reduced by activation, we examined whether this applied to GABA$_B$Rs in axonal-extrasynaptic and presynaptic domains of Syn-eGFP-expressing neurons. Baclofen (100 μM; <5 min) did not alter the relative distribution of R1aR2 among the three membrane domains, but their mobilities were reduced compared to non-activated controls ($p < 0.001$, KS test; Figures 2D and 2E), with axonal-extrasynaptic receptors showing more-pronounced retardation and confinement (Figure 2F). Consequently, the mean dwell time of presynaptic R1aR2 receptors was increased by baclofen ($p < 0.001$; Figure 2G). In addition, the ratio of exchanging receptors (between presynaptic and axonal-extrasynaptic domains) to total presynaptic receptors (Renner et al., 2009) was higher for control R1aR2s (0.81; n = 441) compared to after baclofen (0.68; n = 515), reflecting an increased residence of receptors at presynaptic terminals post-baclofen. Overall, these results indicate that activation of R1aR2s reduces their mobility at presynaptic terminals.

GABA$_B$R Isoforms Have Distinct Lateral Mobility on Axonal Membranes

We next compared the lateral mobility of R1bR2s on axons with R1aR2s. Although R1bR2s are predominantly postsynaptic, they are found in axonal membranes (Biermann et al., 2010). R1bR2s were mostly in the axonal-extrasynaptic domain, with a smaller population in presynaptic compartments and the remainder in the exchanging pool.

Axonal-extrasynaptic receptors were more mobile (higher *D*) than presynaptic R1bR2s ($p < 0.01$, KS; Figures 3A and 3B); both of these pools had higher *D* values compared to their R1aR2 equivalents (Figure 3B). The greater mobility of presynaptic R1bR2s was associated with reduced confinement compared to presynaptic R1aR2s ($p < 0.001$, KS test; Figures 3A and 3C), and consequently, the synaptic dwell time of presynaptic R1bR2s was also lower than that for R1aR2s ($p < 0.05$, unpaired two-tailed t test; Figure 3D). These results indicated that R1bR2s are less constrained than R1aR2s on axonal membranes.

OPEN ACCESS
CellPress

The effect of agonist activation on R1bR2 lateral mobility was also assessed. Baclofen did not alter their relative localization, diffusion coefficients ($p > 0.05$; Figures 3E and 3F), or synaptic dwell times ($p > 0.05$; Figure 3G), in stark contrast to the slower mobility observed for R1aR2s (Figures 2D–2F).

These results indicate that single $GABA_B$Rs are laterally mobile, with cell surface presynaptic receptors recruited from axonal-extrasynaptic areas. R1aR2s and R1bR2s have distinct mobility profiles at axon terminals, and only the trafficking of presynaptic R1aR2s is regulated by activation.

SDs Regulate the Mobility of Presynaptic R1aR2s

We hypothesized that activation affected presynaptic R1aR2s mobility via the SDs, possibly interacting with extracellular partners. SDs are reported to also increase the residence of R1aR2s on the cell surface, compared to R1bR2s, by slowing their internalization (Hannan et al., 2012). We therefore studied whether the SDs affected R1aR2 mobility.

From the N terminus, deleting either the first ($R1a^{\Delta SD1}$R2) or second (downstream) SD ($R1a^{\Delta SD2}$R2), presynaptic $R1a^{\Delta SD1}$R2s or $R1a^{\Delta SD2}$R2s were still less mobile after baclofen compared to controls ($p < 0.001$; $p < 0.05$, KS test; Figures 3H and 3I), suggesting that only one SD need be present to slow R1aR2 mobility after activation.

Although the presynaptic and axonal-extrasynaptic diffusion coefficients for non-activated $R1a^{\Delta SD1}$R2 receptors were similar ($p > 0.05$, KS test; Figure S3), the D for presynaptic $R1a^{\Delta SD1}$R2s is higher than that for R1aR2s ($p < 0.001$, KS test; Figure S3), whereas the D for axonal-extrasynaptic $R1a^{\Delta SD1}$R2s is lower (Figure S3). These data further indicate the importance of SDs in determining the mobility of R1aR2s in axonal membranes and in accumulating receptors at presynaptic terminals. In comparison, $R1a^{\Delta SD2}$R2s behaved in a similar manner to $R1a^{\Delta SD1}$R2s (Figure S3), although the extent to which diffusion was reduced by baclofen was lower for the SD2 deletion, highlighting the crucial role SD1 plays in determining resting presynaptic $GABA_B$R mobility. This suggests that, whereas the absence of the SDs in R1b renders $GABA_B$Rs insensitive to changes in lateral diffusion upon activation, either one of the SDs can reduce diffusion of activated R1aR2s, and when combined, this effect is increased.

Glutamate Receptors Modulate $GABA_B$R Mobility

A primary role for R1aR2s at presynaptic glutamatergic terminals is to reduce glutamate release (Bettler et al., 2004). Presynaptic terminals and nearby $GABA_B$Rs will be exposed to high transient levels of glutamate during excessive release. We therefore investigated whether such increases in glutamate concentration affected the mobility of single R1aR2s at presynaptic terminals, as a mechanism for mitigating excitotoxic events.

Applying 30-μM glutamate (chosen as it is ~5-fold lower than measured extrasynaptic spillover levels but more than sufficient to activate perisynaptic NMDARs; Dzubay and Jahr, 1999; Rusakov and Kullmann, 1998) to neurons significantly reduced the mobility of axonal-extrasynaptic ($p < 0.001$, KS test) and presynaptic R1aR2s ($p < 0.01$, KS test; Figures 4A and 4F) compared to untreated cells. Glutamate did not alter the confinement patterns of either population (Figure 4B), and consistent with a reduced D, the presynaptic dwell time of $GABA_B$Rs was increased ($p < 0.01$, unpaired two-tailed t test; Figure 4C). Moreover, the ratio of exchanging to total presynaptic receptors was also reduced by glutamate (0.63; n = 187), in accord with an accumulation of presynaptic $GABA_B$Rs. Thus, like baclofen, but to a lesser extent ($p < 0.001$, KS; Figure S4), glutamate reduced the mobility of R1aR2s in axons.

To determine the glutamate receptor subtypes involved in accumulating terminal $GABA_B$Rs, the AMPA receptor antagonist CNQX and NMDAR antagonist APV were used. Combining CNQX and APV prevented the reduction in mobility and accumulation of presynaptic $GABA_B$Rs by glutamate. The D for presynaptic R1aR2s was not reduced by glutamate in CNQX and APV ($p > 0.05$, KS test) but increased compared to D for $GABA_B$Rs exposed to glutamate alone ($p < 0.001$, KS test; Figures 4D and 4F). This indicated that presynaptic $GABA_B$R mobility is modulated by activated ionotropic glutamate receptors.

By contrast, glutamate (in CNQX and APV) still reduced the mobility of axonal-extrasynaptic $GABA_B$Rs compared to controls or glutamate alone ($p < 0.001$, KS test; Figures 4E and 4F), suggesting this effect is likely to be mediated by activated metabotropic glutamate receptors (data not shown). The reduction of $GABA_B$R mobility was unlikely to be due to network-driven effects of glutamate because this was unaffected by tetrodotoxin (TTX) ($p < 0.001$, KS test; Figures 4D–4F).

NMDAR Activation Recruits $GABA_B$Rs to Presynaptic Terminals

We next studied which GluR isoform was involved in the accumulation of $GABA_B$Rs at presynaptic terminals. In comparison with untreated neurons, NMDAR activation by glutamate in CNQX slowed the mobility of presynaptic $GABA_B$Rs, reducing D ($p < 0.001$, KS; Figures S5A–S5D) to that for presynaptic $GABA_B$Rs exposed to glutamate alone ($p > 0.05$). The dwell time was also increased ($p < 0.001$, one-way ANOVA; Figure S5D), equivalent to the dwell time in glutamate alone ($p > 0.05$, one-way ANOVA). These results strongly suggest that glutamate activation of NMDARs, and not AMPA receptors (AMPARs), retard the mobility of $GABA_B$Rs.

These findings were corroborated by co-applying glutamate and APV, which prevented the reduction in D for presynaptic $GABA_B$Rs compared to controls (Figures 5A and 5B; $p > 0.05$, KS test), whereas glutamate alone again reduced the lateral mobility of presynaptic $GABA_B$Rs compared either to controls ($p < 0.001$, KS test) or to glutamate and APV (Figures 5A and 5B; $p < 0.01$, KS test).

To demonstrate unequivocally that NMDAR activation alone reduced presynaptic $GABA_B$R diffusion, we co-applied NMDA and D-serine. This reduced $GABA_B$R mobility with a corresponding decrease in D for presynaptic $GABA_B$Rs (Figures 5A and 5B; $p < 0.001$, KS test), coupled with increased confinement (Figure 5C) and a reduced confinement area (Figure 5D; $p < 0.001$, KS test). As observed with glutamate alone, the presynaptic dwell time of $GABA_B$Rs was significantly increased by NMDAR activation (Figure 5E; $p < 0.05$, two-tailed unpaired t test). These results together with the reduction in diffusion confirm that

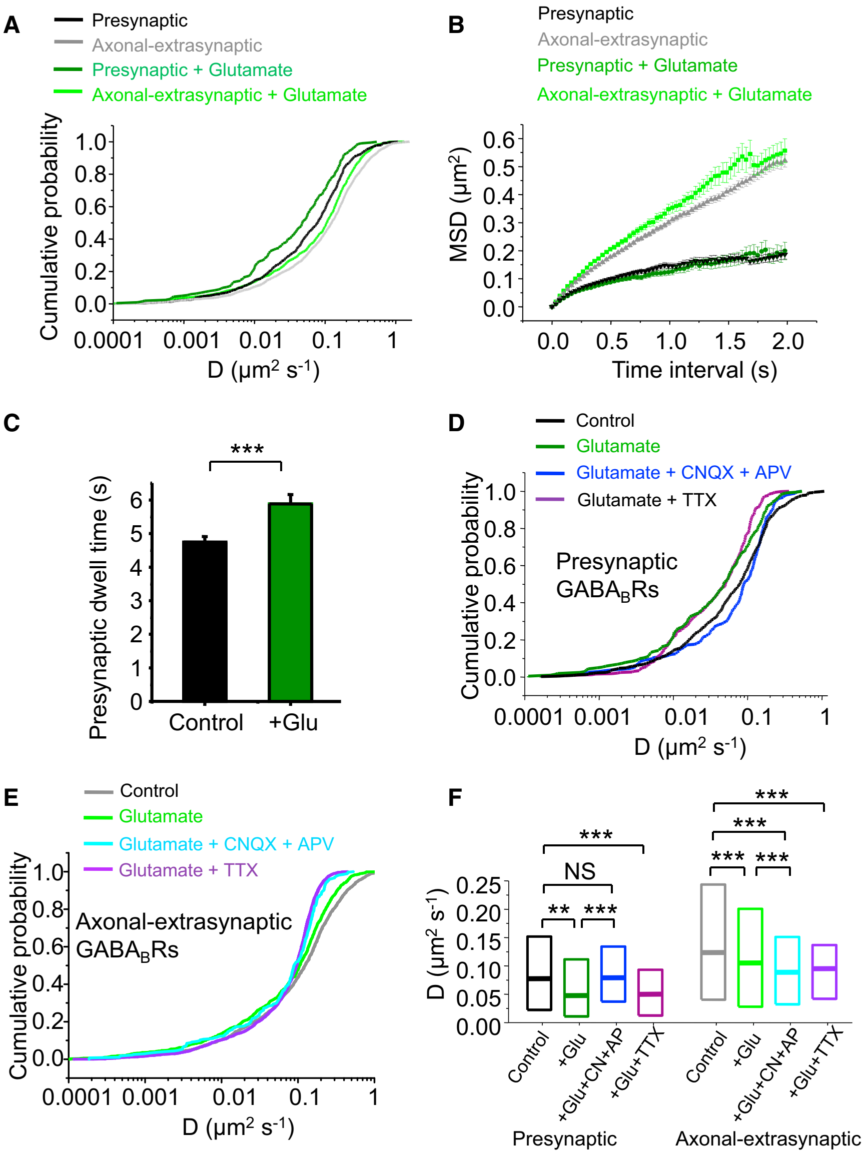

Figure 4. GluR Activation Reduces $GABA_BR$ Diffusion, Causing Recruitment to Presynaptic Terminals

(A–C) Cumulative probabilities (A), MSDs (B), and mean presynaptic dwell times (C) for presynaptic and axonal-extrasynaptic R1aR2s in control Krebs and in 30 μM glutamate.

(D and E) Cumulative probabilities for *D* of presynaptic (D) and axonal extrasynaptic (E) R1aR2s in control; +glutamate; +glutamate, CNQX (10 μM), and APV (100 μM); or +glutamate and tetrodotoxin (TTX) (0.5 μM).

(F) Median *D* and IQR for presynaptic and axonal-extrasynaptic R1bR2s in control, +glutamate (Glu), or +glutamate with CNQX (CN) and APV (AP).

**$p < 0.01$; ***$p < 0.001$ KS test and two-tailed unpaired t test (see also Figures S4 and S5).

signaling via NMDARs accumulates $GABA_BRs$ in presynaptic terminals by reducing their mobility.

$GABA_BR$ Lateral Mobility Is Dependent on Elevating Internal Ca^{2+}

Ca^{2+} permeation via NMDARs subsequently increasing internal Ca^{2+} is important for cellular signaling, including long-term potentiation (Lüscher and Malenka, 2012; Madison et al., 1991). To assess whether increasing internal Ca^{2+} is necessary for accumulating presynaptic $GABA_BRs$, glutamate was applied in the presence of the membrane permeant Ca^{2+} chelator, BAPTA-AM.

The diffusion of presynaptic R1aR2s was significantly faster with BAPTA-AM (Figures 5F and 5G; $p < 0.05$, KS). Values of *D* for R1aR2s in glutamate alone and glutamate plus vehicle were similar (Figure S5) but increased in glutamate and BAPTA-AM, approaching that for untreated R1aR2s (Figures S5E and S5F). Significantly, the presynaptic dwell time of $GABA_BRs$ in glutamate was reduced by BAPTA-AM (Figure 5H; $p < 0.05$, unpaired two-tailed t test). Thus, elevated internal Ca^{2+} via NMDARs is required for accumulating $GABA_BRs$ at presynaptic terminals via lateral diffusion.

NMDAR Activation Causes the Accumulation of Native Presynaptic $GABA_BRs$

Having established that NMDAR activation reduces the mobility of presynaptic $GABA_BRs$ via internal Ca^{2+}, we studied the accumulation of *native* $GABA_BRs$ at presynaptic terminals in permeabilized, cultured hippocampal neurons following NMDAR activation. Neurons were incubated in vehicle, glutamate, and NMDA and D-serine or glutamate and BAPTA-AM before fixation in paraformaldehyde (PFA), permeabilization, and labeling with antibodies for synaptophysin (Syn) and $GABA_BR2$.

Applying either glutamate or NMDA and D-serine (5 min) increased the fluorescence intensity for $GABA_BRs$ (Figures 6A–6C; $p < 0.001$, Mann-Whitney [MW] test) at presynaptic, Syn-positive terminals. The co-application of BAPTA-AM with glutamate prevented this effect ($p > 0.05$; MW). In addition, glutamate also clearly increased cell surface presynaptic $GABA_BR$ staining assessed with BgTx-Alexa Fluor 555 labeling (Figures S6A–S6D). Together, these results from permeabilized and intact neurons are in accord with activated NMDARs increasing the accumulation of native $GABA_BRs$ at presynaptic terminals.

Presynaptic $GABA_BR$ Recruitment Requires Phosphorylation of Ser783 in R2

To examine how NMDAR activation recruits $GABA_BRs$ to presynaptic terminals, we assessed the role of phosphorylation.

OPEN
ACCESS
CellPress

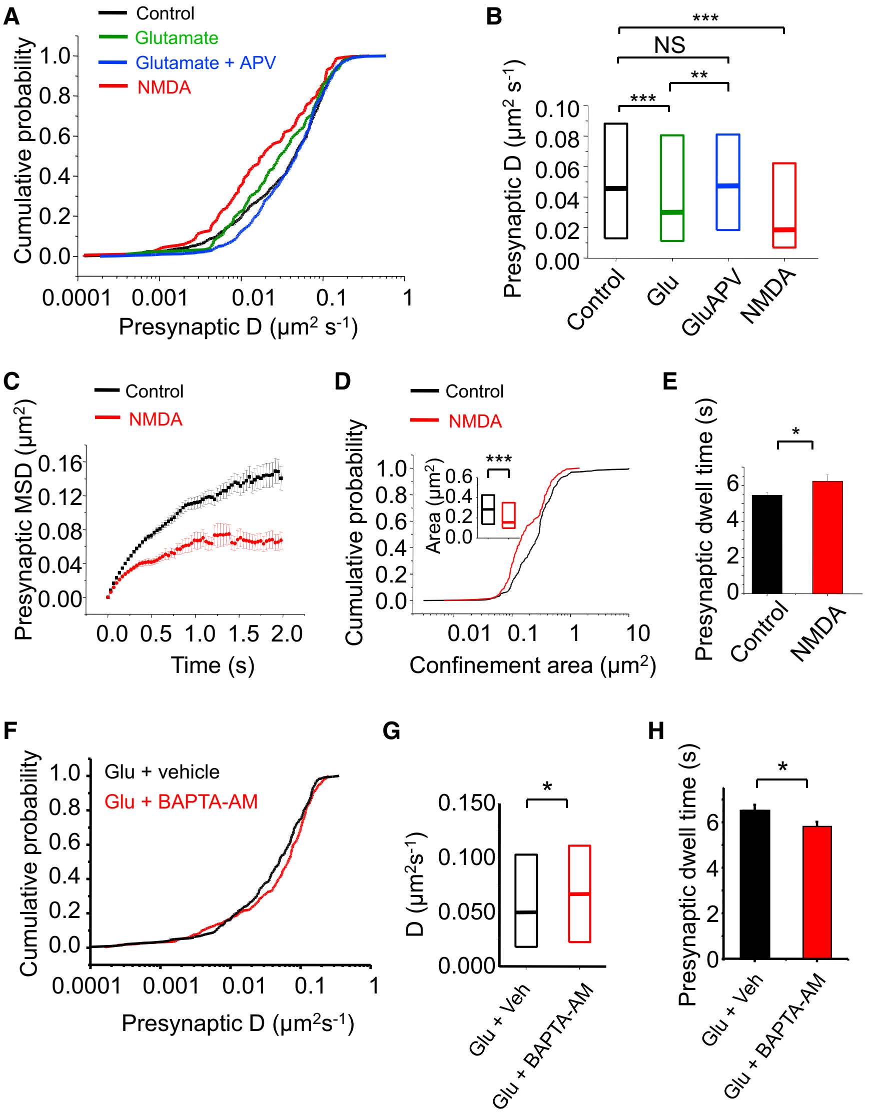

Figure 5. NMDAR Activation Increases Presynaptic Dwell Time of GABA$_B$Rs

(A) Cumulative probabilities of *D* for presynaptic R1aR2s in control, +30 μM glutamate (Glu), +30 μM glutamate and 100 μM APV, or +30 μM NMDA and 10 μM D-serine (NMDA).

(B) Medians and IQR for presynaptic *D* of R1aR2 from (A).

(C) MSDs for presynaptic GABA$_B$Rs in control and in NMDA.

(D) Cumulative probabilities of confinement areas for presynaptic GABA$_B$Rs in control and NMDA (inset—median and IQR).

(E) Presynaptic dwell times of GABA$_B$Rs in control and NMDA.

(F) Cumulative probabilities of presynaptic GABA$_B$R *D* in glutamate for vehicle-treated controls or +20 μM BAPTA-AM.

(G) Median and IQR of presynaptic *D* for GABA$_B$Rs from (F).

(H) Presynaptic dwell times of GABA$_B$Rs in glutamate + vehicle (n = 192) or +BAPTA-AM (n = 336). *p < 0.05; ***p < 0.001 (see also Figure S5).

GABA$_B$Rs are substrates for protein kinases (Couve et al., 2002; Guetg et al., 2010) with 5′ AMP-activated protein kinase (AMPK) (Kuramoto et al., 2007) of particular interest because it phosphorylates GABA$_B$R2 Ser783 following NMDAR activation (Terunuma et al., 2010). We investigated whether glutamate-induced accumulation of GABA$_B$Rs is affected by R2 phosphorylation using the mutation S783A.

The diffusion of presynaptic R1aR2^{S783A} in control and in glutamate was similar to that for untreated R1aR2s (Figures 7A and 7B; p > 0.05, KS) but higher compared to R1aR2s in glutamate-treated neurons (p < 0.001; KS). This was reflected by shorter presynaptic dwell times for R1aR2^{S783A} in control and in glutamate, and untreated R1aR2s, compared to R1aR2s in glutamate (Figure 7C; p < 0.05, unpaired t test). These results suggest that phosphorylating S783 after NMDAR activation is critically important for accumulating GABA$_B$Rs at presynaptic terminals.

The role of phosphorylation in the recruitment of native presynaptic GABA$_B$Rs by glutamate was also studied in cultured hippocampal neurons using immunolabeling with a GABA$_B$R2 phospho (p)-783-specific antibody. Glutamate increased fluorescence intensity labeling with p783 in Syn co-labeled presynaptic terminals (Figures S6E–S6G; p < 0.001, MW test). In addition, glutamate failed to increase the presynaptic accumulation of phospho-mutant GABA$_B$Rs on the cell surface (Figures S6A, S6C, and S6D), and consistent with this, the S783A mutation reduced the localization of GABA$_B$Rs at presynaptic terminals (Figure S7). These data corroborate the QD experiments, suggesting phosphorylation of S783 is key to glutamate-induced accumulation of GABA$_B$Rs at presynaptic terminals.

GABA$_B$R2^{S783A} Potentiates Presynaptic NMDAR-Mediated Ca^{2+} Signaling

To understand how NMDAR activation and Ca^{2+} signaling combine to affect GABA$_B$R mobility, we monitored Ca^{2+} signals in hippocampal neurons expressing the genetically encoded Ca^{2+} sensor GCaMP6 fused to Syn (Syn-GCaMP6Fast; Zhao et al., 2011) with either R1aR2 or R1aR2^{S783A}. Basal Ca^{2+} levels were similar, but after applying NMDA and D-serine to R1aR2^{S783A}-expressing neurons, the maximum peak Ca^{2+} transients in presynaptic terminals was elevated compared to neurons expressing R1aR2 (p < 0.001, MW test; Figures 7D–7F). In addition, the mean amplitude of Ca^{2+} transients was also greater for R1aR2^{S783A} neurons compared to R1aR2s (p < 0.001, MW test; Figure 7G).

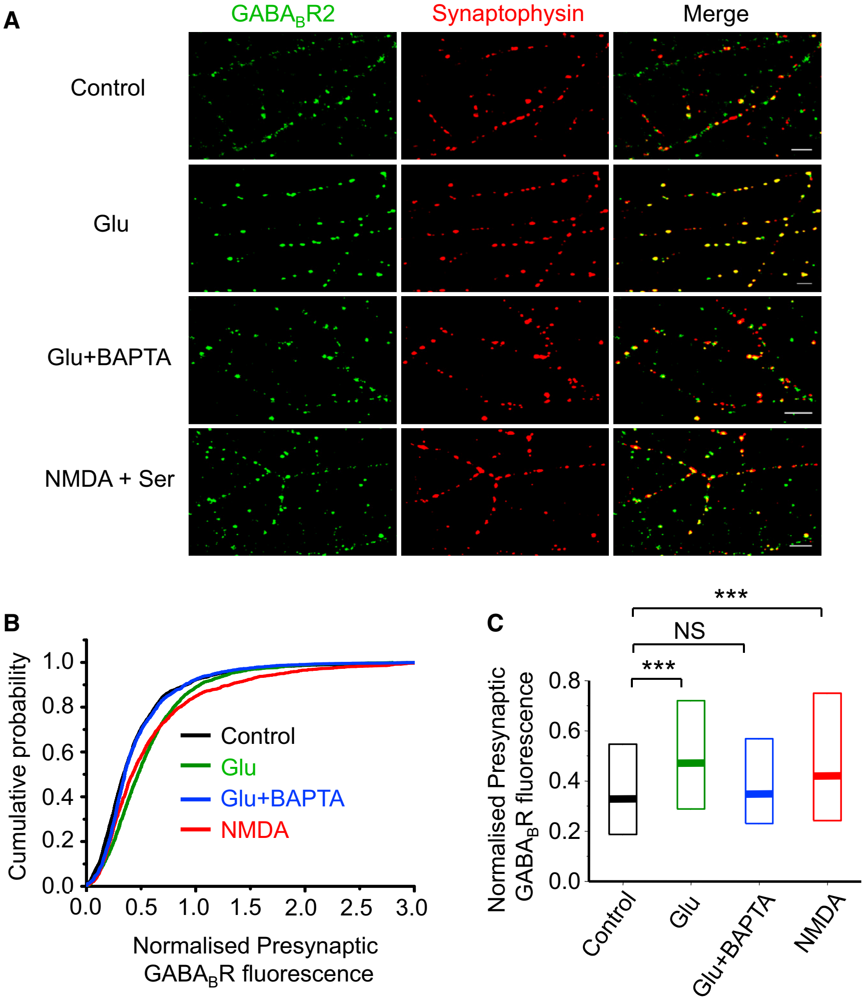

Figure 6. NMDAR Activation Increases GABA$_B$Rs Accumulation at Presynaptic Terminals

(A) Co-localization of native GABA$_B$R2 and synaptophysin in permeabilized hippocampal neurons (14 DIV) in control, +30 μM glutamate (Glu), and +30 μM Glu and 20 μM BAPTA-AM, or +30 μM NMDA and 10 μM D-serine (NMDA).

(B) Cumulative probabilities for presynaptic GABA$_B$R2 fluorescence normalized to synaptophysin fluorescence in control, +glutamate, +glutamate and BAPTA-AM, and NMDA.

(C) Median values for normalized GABA$_B$R2 presynaptic fluorescence in control (n = 2,916 puncta), glutamate (n = 1,830), glutamate and BAPTA-AM (n = 1,832), and NMDA + serine (n = 1,535).

***$p < 0.001$ (see also Figure S6).

This NMDAR-driven comparative increase in Ca^{2+} signaling was unexpected for neurons expressing R1aR2^{S783A}. We had predicted that the increased Ca^{2+} signal would reduce D and increase the dwell time for GABA$_B$Rs at presynaptic terminals, but neither change occurred (Figures 7B and 7C). Therefore, although increased Ca^{2+} influx recruits GABA$_B$Rs to the presynaptic terminal, it is less effective in recruiting mutant (S783A) GABA$_B$Rs, suggesting phosphorylation of S783A is the critical factor and must be "downstream" in the signaling pathway for accumulating presynaptic GABA$_B$Rs. It is also conceivable that a reduction in GABA$_B$R numbers at the presynaptic terminal, as a result of S783A (Figure S7), may be responsible for reduced presynaptic inhibition, leading to an increase in terminal Ca^{2+} flux via NMDARs and voltage-gated Ca^{2+} channels. Together, these results highlight the critical role of GABA$_B$R phosphorylation for presynaptic receptor accumulation, an important facet in reducing neurotransmitter release after the activation of NMDARs.

DISCUSSION

Lateral diffusion is important for distributing receptors in postsynaptic membranes (Choquet and Triller, 2013). Fluorescence recovery after photobleaching suggests GABA$_B$Rs are mobile on Cos7 cells and hippocampal neurons (Pooler and McIlhinney, 2007), but whether lateral mobility is important for accumulating and dispersing *presynaptic* receptors is poorly understood (Gomez-Varela and Berg, 2013). Although axonal and dendritic membranes have similar properties, there will be more physical constraints for diffusion in the presynaptic membrane, given the specialist role it plays in neurotransmitter release. Here, we demonstrate the importance of lateral diffusion as a mechanism for accumulating GABA$_B$Rs at axon terminal membranes for eventual modulation of excitatory transmitter release. By studying the diffusion of single GABA$_B$Rs with QDs, their recruitment into axonal membranes can be visualized and the underlying mechanisms examined.

Lateral diffusion of GABA$_B$Rs was resolved by inserting a BBS into GABA$_B$Rs, enabling single-particle tracking with reporter QDs. Such BgTx conjugates have been used for tracking nicotinic acetylcholine receptor α3 and α7 subunits (Bürli et al., 2010; Fernandes et al., 2010). The BBS mimotope (13 amino acids compared to eGFP, which is ~240 amino acids) enables fast labeling and imaging that avoids complications with receptor internalization from the cell surface. The insertion of the BBS into R1a, R1b, or R2 subunits neither altered the trafficking nor function of GABA$_B$Rs (reviewed in Hannan et al., 2013); its high affinity in R1a (Hannan et al., 2011) and R1b (Hannan et al., 2012) for BgTx allowed the specific labeling of GABA$_B$Rs using titrated amounts of QDs. This avoids any confounds that might result from nonspecific interactions between the polyethylglycol coating of the QDs with biological membranes. The smaller size of BgTx (~24 nm^3) compared to primary and secondary antibody complexes (~500 nm^3; Hannan et al., 2013) that are routinely used in QD imaging also makes this method suitable for monitoring diffusion where space constraints exist, such as at synapses.

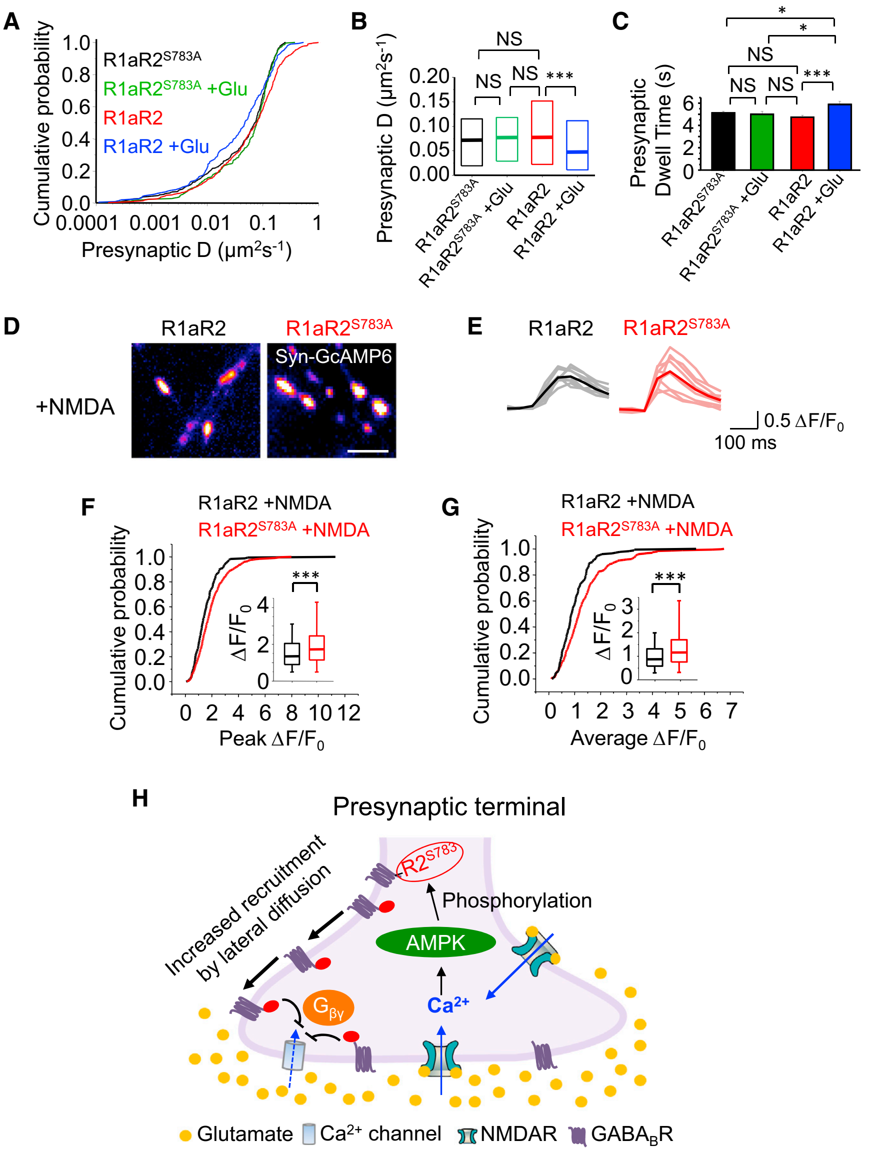

Figure 7. Glutamate Reduces $GABA_BR$ Mobility by Phosphorylating $R2^{S783}$

(A) Cumulative probabilities for presynaptic *D* of R1aR2 and $R1aR2^{S783A}$ in control and +30 μM glutamate (Glu).

(B) Median *D* and IQR values from (A).

(C) Presynaptic dwell times for R1aR2 and $R1aR2^{S783A}$ with or without Glu.

(D) Ca^{2+} signals evoked by 30 μM NMDA and 10 μM D-serine from Syn-GcAMP6 containing presynaptic terminals with either R1aR2 or $R1aR2^{S783A}$. The scale bar represents 2 μm.

(E) Ca^{2+} transients from single presynaptic terminals expressing R1aR2 or $R1aR2^{S783A}$. Averaged transients are shown as dark lines.

(F) Distribution for maximum peak Ca^{2+} transients from presynaptic terminals expressing R1aR2 or $R1aR2^{S783A}$ (inset—5%–95% range, IQR, and median values).

(G) Cumulative probabilities for average Ca^{2+} transients from presynaptic terminals expressing R1aR2 or $R1aR2^{S783A}$ mutants (inset—5%–95% range, IQR, and median values; ***$p < 0.001$).

(H) Mechanism for homeostatic inhibitory control of transmitter release at excitatory terminals. NMDAR activation increases Ca^{2+} in presynaptic terminals and activates AMPK. Phosphorylation of $R2^{S783}$ by AMPK increases lateral recruitment of $GABA_BRs$ into the presynaptic terminal from axonal membranes. Activation of $GABA_BRs$ by GABA spillover reduces Ca^{2+} entry into the terminals (via $G_{\beta\gamma}$ signaling), thereby reducing neurotransmitter release from presynaptic boutons (see also Figures S6 and S7).

The main subtypes of $GABA_BRs$, R1aR2s and R1bR2s, have distinct physiological and pathophysiological roles (Gassmann et al., 2004; Guetg et al., 2009; Pérez-Garci et al., 2006) and differ by the two SDs in R1a (Hawrot et al., 1998). Studies of $GABA_BR$ knockout models have identified a presynaptic role for R1a at glutamatergic terminals, whereas both R1a and R1b form postsynaptic receptors. Monitoring diffusion of these receptor subtypes revealed distinctive mobility profiles, with R1a being less mobile than R1b. The inability of R1b to become "trapped" at presynaptic terminals in response to $GABA_BR$ activation (unlike R1a) established the SDs as key modulators of lateral mobility in this study. This is in addition to their reported roles in transport (Biermann et al., 2010; Vigot et al., 2006) and cell surface stability (Hannan et al., 2012). For accumulating R1a at axon terminals, SD1 nearest the N-terminal is critical, whereas either SD (SD1/2) is capable of slowing R1a mobility after agonist activation. SD1 is most likely to reduce lateral diffusion by transiently interacting with protein partners in the terminal domain, and after agonist activation, presumed changes to receptor conformation may allow either SD to establish interactions, further reducing $GABA_BR$ diffusion at the presynaptic terminal.

Reducing $GABA_BR$ mobility and increasing presynaptic dwell time following activation could be significant in a physiological context. This would provide a mechanism for accumulating presynaptic $GABA_BRs$ for controlling neurotransmitter release. In this regard, $GABA_BRs$ that are perisynaptic to inhibitory synapses will be activated by GABA released directly from interneurons; and at glutamatergic terminals, the high-affinity $GABA_BRs$ will be activated by GABA spillover from neighboring GABAergic synapses, leading to an increased clustering at presynaptic terminals (Dittman and Regehr, 1997; Dutar and Nicoll, 1988; Fritschy et al., 1999; Hirono et al., 2001; Isaacson et al., 1993; Kulik et al., 2002; Scanziani, 2000).

This role for SDs as modulators of lateral diffusion is unique. Their extracellular location contrasts with other interacting proteins that are typically intracellular and modulate lateral diffusion of receptors via interactions with scaffold proteins

(Bannai et al., 2009; Hausrat et al., 2015; Renner et al., 2009). SDs are likely to slow down $GABA_BR$ mobility at synapses by transient interactions with extracellular partners within the presynaptic terminal or with recently identified postsynaptic partners (trans-synaptic; Schwenk et al., 2016) to achieve the same purpose. The latter might also explain the paucity of interacting partners for the SDs reported to date (Blein et al., 2004).

Accumulating even relatively few "membrane stable" $GABA_BRs$ at synapses is likely to be a crucial determinant for the efficacy of excitatory transmission and associated cellular plasticity. Several studies have investigated $GABA_BR$ trafficking after sustained (>5 min) activation of ionotropic GluRs. These studies reported reduced cell surface $GABA_BR$ levels after prolonged NMDAR activation caused by increased rates of internalization (Guetg et al., 2010; Terunuma et al., 2010) and/or increased lysosomal degradation (Maier et al., 2010) of $GABA_BRs$. However, the residence of cell surface receptors at synaptic membrane microdomains has not been investigated under resting conditions or in agonist-activated neurons. Here, by briefly activating NMDARs (<5 min) to replicate physiologically relevant conditions, the subsequent elevation of intracellular Ca^{2+} (Hardie et al., 2012; Hawley et al., 2005; Weisová et al., 2009; Woods et al., 2005) will lead to increased phosphorylation of $GABA_BR2$ at S783 by AMPK, causing an increase in the density of $GABA_BRs$ at presynaptic terminals by reducing their lateral mobility (Figure 7H).

Even using prolonged periods of NMDAR activation, increased cell surface expression of $GABA_BRs$ and R2 subunit phosphorylation are observed at early time points (~5 min; Terunuma et al., 2010), a feature also noted in a recent study that assessed the cell surface stability of $GABA_BRs$ in response to activity-dependent changes over a short time period (Kantamneni et al., 2014). These reports are consistent with the increased presynaptic accumulation of $GABA_BRs$ observed in our study. During prolonged pathophysiological conditions, including traumatic brain injury and ischemia, prolonged and sustained activation of NMDARs will likely cause the activation of the phosphatase PP2A (Terunuma et al., 2010), leading to dephosphorylation of S783, decreasing the number of surface $GABA_BRs$ by increasing internalization and degradation.

The importance of phosphorylation in the recruitment of presynaptic $GABA_BRs$ is highlighted by the AMPK phosphorylation mutant, S783, on $GABA_BR2$, which shows reduced localization to presynaptic terminals in response to glutamate. Interestingly, Ca^{2+} signaling was elevated in response to NMDAR activation in neurons expressing $R2^{S783A}$. This most likely reflects a reduction in presynaptic $GABA_BRs$, as lateral diffusion is impaired by $R2^{S783A}$ and is unlikely to be due to internalization with such a brief exposure to NMDA (Hannan et al., 2011).

We would propose the following model for $GABA_BR$ mobility at presynaptic excitatory synapses (Figure 7H). Pre- or postsynaptic NMDAR activation would initiate Ca^{2+} influx via NMDAR channels (Banerjee et al., 2016; Duguid and Smart, 2009) and, eventually, either directly or by retrograde transmitter release (Bouvier et al., 2015; Casado et al., 2000; Duguid and Smart, 2004), increase terminal Ca^{2+} levels following Ca^{2+} channel activation and/or by internal Ca^{2+} release. In terminal membranes, this would initially increase glutamate release (Bouvier et al., 2015) but also enable AMPK activation to phosphorylate S783 on R2 subunits (Kuramoto et al., 2007), slowing $GABA_BR$ mobility in the terminal membrane and promoting receptor accumulation to increase presynaptic inhibition once $GABA_BRs$ are activated. Reducing the internal Ca^{2+} rise, or ablating phosphorylation at $R2^{S783}$, is sufficient to reduce $GABA_BR$ accumulation. The rise in internal Ca^{2+}, caused by NMDA, in neurons expressing phosphorylation mutant $R2^{S783A}$ and the failure of this increase to affect receptor mobility strongly suggests that internal Ca^{2+} effects are mediated by phosphorylation of S783 and that this is the critical determinant of $GABA_BR$ mobility.

Thus, linking NMDAR activation with a signaling pathway involving internal Ca^{2+} and phosphorylation of $GABA_BRs$ that reduces the lateral mobility and increases the recruitment of $GABA_BRs$ to the presynaptic terminal membrane is a potentially powerful homeostatic mechanism for preventing excessive signaling and glutamate-mediated excitotoxicity.

EXPERIMENTAL PROCEDURES

For further details, see Supplemental Information. The $GABA_BR1$ isoforms ($R1a^{BBS}$ and $R1b^{BBS}$) containing a BBS, a flag-tagged $GABA_BR2$ (R2), $R1a^{BBS}$ with SD deletion ($R1a^{\Delta SD1}$ or $R1a^{\Delta SD2}$), an $R2^{S783A}$ mutant, and pEGFP-C1 have been described previously (Hannan et al., 2011, 2012; Kuramoto et al., 2007). All drugs and chemicals were acquired from Sigma unless specified otherwise. All experiments were performed in accordance with the UK Animals (Scientific Procedures) Act 1986. Dissociated hippocampal cultures were prepared from embryonic day 18 (E18) Sprague-Dawley rat embryos and transfected at 7 days in vitro (DIV) as described previously (Hannan et al., 2011). For QD labeling, at 12–14 DIV, BBS-containing $GABA_BRs$ were incubated in 4 μg/ml BgTx-B (Life Technologies) for 2 min at 37°C before incubation with QD655 conjugated to streptavidin (Life Technologies). For immunostaining, permeabilized neurons were incubated in primary antibodies ($GABA_BR2$ [Neuromab], phospho-783 $GABA_BR2$ [Santa Cruz Biotechnology], Syn [Abcam], PSD95 [Neuromab], and gephyrin [Synaptic Systems]) followed by secondary antibodies conjugated with Alexa 488, 555, or 594 (Life Technologies) prior to imaging. For cell surface labeling, BBS-containing $GABA_BRs$ were incubated in 4 μg/ml BgTx Alexa Fluor 555 (Life Technologies) for 10 min at room temperature (RT). For Ca^{2+} imaging, Syn-GcAMP6Fast Ca^{2+} transients were captured in the presence of brief applications of NMDA and D-serine before analysis of signals using Matlab. Whole-cell electrophysiology was performed as described in Supplemental Information.

SUPPLEMENTAL INFORMATION

Supplemental Information includes Supplemental Experimental Procedures, seven figures, one table, and three movies and can be found with this article online at http://dx.doi.org/10.1016/j.celrep.2016.07.021.

AUTHOR CONTRIBUTIONS

S.H. and T.G.S. designed the project and wrote the manuscript. S.H. undertook the electrophysiology, quantum dot, and imaging experiments and analyzed the data. K.G. processed the presynaptic markers for localization. A.T. contributed to the algorithms used for analyzing the quantum dot trajectories. All contributed to the writing of the manuscript.

ACKNOWLEDGMENTS

This work was supported by the MRC UK and by The Rosetrees Trust.

OPEN
ACCESS
CellPress

Received: November 30, 2015
Revised: April 19, 2016
Accepted: July 9, 2016
Published: August 4, 2016

REFERENCES

Banerjee, A., Larsen, R.S., Philpot, B.D., and Paulsen, O. (2016). Roles of presynaptic NMDA receptors in neurotransmission and plasticity. Trends Neurosci. *39*, 26–39.

Bannai, H., Lévi, S., Schweizer, C., Inoue, T., Launey, T., Racine, V., Sibarita, J.B., Mikoshiba, K., and Triller, A. (2009). Activity-dependent tuning of inhibitory neurotransmission based on $GABA_AR$ diffusion dynamics. Neuron *62*, 670–682.

Benke, D. (2013). $GABA_B$ receptor trafficking and interacting proteins: targets for the development of highly specific therapeutic strategies to treat neurological disorders? Biochem. Pharmacol. *86*, 1525–1530.

Benke, D., Zemoura, K., and Maier, P.J. (2012). Modulation of cell surface $GABA_{(B)}$ receptors by desensitization, trafficking and regulated degradation. World J. Biol. Chem. *3*, 61–72.

Benke, D., Balakrishnan, K., and Zemoura, K. (2015). Regulation of cell surface $GABA_{(B)}$ receptors: contribution to synaptic plasticity in neurological diseases. Adv. Pharmacol. *73*, 41–70.

Bettler, B., and Tiao, J.Y. (2006). Molecular diversity, trafficking and subcellular localization of $GABA_B$ receptors. Pharmacol. Ther. *110*, 533–543.

Bettler, B., Kaupmann, K., Mosbacher, J., and Gassmann, M. (2004). Molecular structure and physiological functions of $GABA_{(B)}$ receptors. Physiol. Rev. *84*, 835–867.

Biermann, B., Ivankova-Susankova, K., Bradaia, A., Abdel Aziz, S., Besseyrias, V., Kapfhammer, J.P., Missler, M., Gassmann, M., and Bettler, B. (2010). The sushi domains of $GABA_B$ receptors function as axonal targeting signals. J. Neurosci. *30*, 1385–1394.

Blein, S., Ginham, R., Uhrin, D., Smith, B.O., Soares, D.C., Veltel, S., McIlhinney, R.A., White, J.H., and Barlow, P.N. (2004). Structural analysis of the complement control protein (CCP) modules of $GABA_{(B)}$ receptor 1a: only one of the two CCP modules is compactly folded. J. Biol. Chem. *279*, 48292–48306.

Borgdorff, A.J., and Choquet, D. (2002). Regulation of AMPA receptor lateral movements. Nature *417*, 649–653.

Bouvier, G., Bidoret, C., Casado, M., and Paoletti, P. (2015). Presynaptic NMDA receptors: roles and rules. Neuroscience *311*, 322–340.

Bürli, T., Baer, K., Ewers, H., Sidler, C., Fuhrer, C., and Fritschy, J.M. (2010). Single particle tracking of alpha7 nicotinic AChR in hippocampal neurons reveals regulated confinement at glutamatergic and GABAergic perisynaptic sites. PLoS ONE *5*, e11507.

Casado, M., Dieudonné, S., and Ascher, P. (2000). Presynaptic N-methyl-D-aspartate receptors at the parallel fiber-Purkinje cell synapse. Proc. Natl. Acad. Sci. USA *97*, 11593–11597.

Choi, D.W. (1987). Ionic dependence of glutamate neurotoxicity. J. Neurosci. *7*, 369–379.

Choquet, D., and Triller, A. (2013). The dynamic synapse. Neuron *80*, 691–703.

Cimarosti, H., Kantamneni, S., and Henley, J.M. (2009). Ischaemia differentially regulates $GABA_{(B)}$ receptor subunits in organotypic hippocampal slice cultures. Neuropharmacology *56*, 1088–1096.

Couve, A., Thomas, P., Calver, A.R., Hirst, W.D., Pangalos, M.N., Walsh, F.S., Smart, T.G., and Moss, S.J. (2002). Cyclic AMP-dependent protein kinase phosphorylation facilitates $GABA_{(B)}$ receptor-effector coupling. Nat. Neurosci. *5*, 415–424.

Davis, G.W. (2006). Homeostatic control of neural activity: from phenomenology to molecular design. Annu. Rev. Neurosci. *29*, 307–323.

Dittman, J.S., and Regehr, W.G. (1997). Mechanism and kinetics of heterosynaptic depression at a cerebellar synapse. J. Neurosci. *17*, 9048–9059.

Duguid, I.C., and Smart, T.G. (2004). Retrograde activation of presynaptic NMDA receptors enhances GABA release at cerebellar interneuron-Purkinje cell synapses. Nat. Neurosci. *7*, 525–533.

Duguid, I.C., and Smart, T.G. (2009). Presynaptic NMDA receptors. In Biology of NMDAR (New York: Taylor Francis), pp. 313–328.

Dutar, P., and Nicoll, R.A. (1988). A physiological role for $GABA_B$ receptors in the central nervous system. Nature *332*, 156–158.

Dzubay, J.A., and Jahr, C.E. (1999). The concentration of synaptically released glutamate outside of the climbing fiber-Purkinje cell synaptic cleft. J. Neurosci. *19*, 5265–5274.

Fernandes, C.C., Berg, D.K., and Gómez-Varela, D. (2010). Lateral mobility of nicotinic acetylcholine receptors on neurons is determined by receptor composition, local domain, and cell type. J. Neurosci. *30*, 8841–8851.

Fritschy, J.M., and Panzanelli, P. (2014). $GABA_A$ receptors and plasticity of inhibitory neurotransmission in the central nervous system. Eur. J. Neurosci. *39*, 1845–1865.

Fritschy, J.M., Meskenaite, V., Weinmann, O., Honer, M., Benke, D., and Mohler, H. (1999). $GABA_B$-receptor splice variants GB1a and GB1b in rat brain: developmental regulation, cellular distribution and extrasynaptic localization. Eur. J. Neurosci. *11*, 761–768.

Gassmann, M., Shaban, H., Vigot, R., Sansig, G., Haller, C., Barbieri, S., Humeau, Y., Schuler, V., Müller, M., Kinzel, B., et al. (2004). Redistribution of $GABA_{B(1)}$ protein and atypical $GABA_B$ responses in $GABA_{B(2)}$-deficient mice. J. Neurosci. *24*, 6086–6097.

Gomez-Varela, D., and Berg, D.K. (2013). Lateral mobility of presynaptic α7-containing nicotinic receptors and its relevance for glutamate release. J. Neurosci. *33*, 17062–17071.

Groc, L., Heine, M., Cognet, L., Brickley, K., Stephenson, F.A., Lounis, B., and Choquet, D. (2004). Differential activity-dependent regulation of the lateral mobilities of AMPA and NMDA receptors. Nat. Neurosci. *7*, 695–696.

Guetg, N., Seddik, R., Vigot, R., Turecek, R., Gassmann, M., Vogt, K.E., Bräuner-Osborne, H., Shigemoto, R., Kretz, O., Frotscher, M., et al. (2009). The $GABA_{B1a}$ isoform mediates heterosynaptic depression at hippocampal mossy fiber synapses. J. Neurosci. *29*, 1414–1423.

Guetg, N., Abdel Aziz, S., Holbro, N., Turecek, R., Rose, T., Seddik, R., Gassmann, M., Moes, S., Jenoe, P., Oertner, T.G., et al. (2010). NMDA receptor-dependent $GABA_B$ receptor internalization via CaMKII phosphorylation of serine 867 in $GABA_{B1}$. Proc. Natl. Acad. Sci. USA *107*, 13924–13929.

Hannan, S., Wilkins, M.E., Dehghani-Tafti, E., Thomas, P., Baddeley, S.M., and Smart, T.G. (2011). γ-aminobutyric acid type B (GABA(B)) receptor internalization is regulated by the R2 subunit. J. Biol. Chem. *286*, 24324–24335.

Hannan, S., Wilkins, M.E., and Smart, T.G. (2012). Sushi domains confer distinct trafficking profiles on $GABA_B$ receptors. Proc. Natl. Acad. Sci. USA *109*, 12171–12176.

Hannan, S., Wilkins, M.E., Thomas, P., and Smart, T.G. (2013). Tracking cell surface mobility of GPCRs using α-bungarotoxin-linked fluorophores. Methods Enzymol. *521*, 109–129.

Hardie, D.G., Ross, F.A., and Hawley, S.A. (2012). AMP-activated protein kinase: a target for drugs both ancient and modern. Chem. Biol. *19*, 1222–1236.

Hausrat, T.J., Muhia, M., Gerrow, K., Thomas, P., Hirdes, W., Tsukita, S., Heisler, F.F., Herich, L., Dubroqua, S., Breiden, P., et al. (2015). Radixin regulates synaptic $GABA_A$ receptor density and is essential for reversal learning and short-term memory. Nat. Commun. *6*, 6872.

Hawley, S.A., Pan, D.A., Mustard, K.J., Ross, L., Bain, J., Edelman, A.M., Frenguelli, B.G., and Hardie, D.G. (2005). Calmodulin-dependent protein kinase kinase-β is an alternative upstream kinase for AMP-activated protein kinase. Cell Metab. *2*, 9–19.

Hawrot, E., Xiao, Y., Shi, Q.L., Norman, D., Kirkitadze, M., and Barlow, P.N. (1998). Demonstration of a tandem pair of complement protein modules in $GABA_{(B)}$ receptor 1a. FEBS Lett. *432*, 103–108.

Hirono, M., Yoshioka, T., and Konishi, S. (2001). $GABA_{(B)}$ receptor activation enhances mGluR-mediated responses at cerebellar excitatory synapses. Nat. Neurosci. *4*, 1207–1216.

Isaacson, J.S., Solís, J.M., and Nicoll, R.A. (1993). Local and diffuse synaptic actions of GABA in the hippocampus. Neuron *10*, 165–175.

Jaskolski, F., and Henley, J.M. (2009). Synaptic receptor trafficking: the lateral point of view. Neuroscience *158*, 19–24.

Kantamneni, S., Gonzàlez-Gonzàlez, I.M., Luo, J., Cimarosti, H., Jacobs, S.C., Jaafari, N., and Henley, J.M. (2014). Differential regulation of $GABA_B$ receptor trafficking by different modes of N-methyl-D-aspartate (NMDA) receptor signaling. J. Biol. Chem. *289*, 6681–6694.

Kulik, A., Nakadate, K., Nyíri, G., Notomi, T., Malitschek, B., Bettler, B., and Shigemoto, R. (2002). Distinct localization of $GABA_{(B)}$ receptors relative to synaptic sites in the rat cerebellum and ventrobasal thalamus. Eur. J. Neurosci. *15*, 291–307.

Kuramoto, N., Wilkins, M.E., Fairfax, B.P., Revilla-Sanchez, R., Terunuma, M., Tamaki, K., Iemata, M., Warren, N., Couve, A., Calver, A., et al. (2007). Phospho-dependent functional modulation of $GABA_{(B)}$ receptors by the metabolic sensor AMP-dependent protein kinase. Neuron *53*, 233–247.

Ladepeche, L., Yang, L., Bouchet, D., and Groc, L. (2013). Regulation of dopamine D1 receptor dynamics within the postsynaptic density of hippocampal glutamate synapses. PLoS ONE *8*, e74512.

Ladépêche, L., Dupuis, J.P., and Groc, L. (2014). Surface trafficking of NMDA receptors: gathering from a partner to another. Semin. Cell Dev. Biol. *27*, 3–13.

Lüscher, C., and Malenka, R.C. (2012). NMDA receptor-dependent long-term potentiation and long-term depression (LTP/LTD). Cold Spring Harb. Perspect. Biol. *4*, pii: a005710.

Madison, D.V., Malenka, R.C., and Nicoll, R.A. (1991). Mechanisms underlying long-term potentiation of synaptic transmission. Annu. Rev. Neurosci. *14*, 379–397.

Maier, P.J., Marin, I., Grampp, T., Sommer, A., and Benke, D. (2010). Sustained glutamate receptor activation down-regulates $GABA_B$ receptors by shifting the balance from recycling to lysosomal degradation. J. Biol. Chem. *285*, 35606–35614.

Marder, E., and Goaillard, J.M. (2006). Variability, compensation and homeostasis in neuron and network function. Nat. Rev. Neurosci. *7*, 563–574.

Meldrum, B.S. (1994). The role of glutamate in epilepsy and other CNS disorders. Neurology *44* (*11, Suppl 8*), S14–S23.

Mikasova, L., Groc, L., Choquet, D., and Manzoni, O.J. (2008). Altered surface trafficking of presynaptic cannabinoid type 1 receptor in and out synaptic terminals parallels receptor desensitization. Proc. Natl. Acad. Sci. USA *105*, 18596–18601.

Moss, S.J., and Smart, T.G. (2001). Constructing inhibitory synapses. Nat. Rev. Neurosci. *2*, 240–250.

Muir, J., Arancibia-Carcamo, I.L., MacAskill, A.F., Smith, K.R., Griffin, L.D., and Kittler, J.T. (2010). NMDA receptors regulate $GABA_A$ receptor lateral mobility and clustering at inhibitory synapses through serine 327 on the $\gamma 2$ subunit. Proc. Natl. Acad. Sci. USA *107*, 16679–16684.

Paoletti, P., Bellone, C., and Zhou, Q. (2013). NMDA receptor subunit diversity: impact on receptor properties, synaptic plasticity and disease. Nat. Rev. Neurosci. *14*, 383–400.

Pérez-Garci, E., Gassmann, M., Bettler, B., and Larkum, M.E. (2006). The GABAB1b isoform mediates long-lasting inhibition of dendritic Ca^{2+} spikes in layer 5 somatosensory pyramidal neurons. Neuron *50*, 603–616.

Pooler, A.M., and McIlhinney, R.A. (2007). Lateral diffusion of the $GABA_B$ receptor is regulated by the $GABA_{B2}$ C terminus. J. Biol. Chem. *282*, 25349–25356.

Renner, M., Choquet, D., and Triller, A. (2009). Control of the postsynaptic membrane viscosity. J. Neurosci. *29*, 2926–2937.

Rusakov, D.A., and Kullmann, D.M. (1998). Extrasynaptic glutamate diffusion in the hippocampus: ultrastructural constraints, uptake, and receptor activation. J. Neurosci. *18*, 3158–3170.

Scanziani, M. (2000). GABA spillover activates postsynaptic $GABA_{(B)}$ receptors to control rhythmic hippocampal activity. Neuron *25*, 673–681.

Schwenk, J., Pérez-Garci, E., Schneider, A., Kollewe, A., Gauthier-Kemper, A., Fritzius, T., Raveh, A., Dinamarca, M.C., Hanuschkin, A., Bildl, W., et al. (2016). Modular composition and dynamics of native $GABA_B$ receptors identified by high-resolution proteomics. Nat. Neurosci. *19*, 233–242.

Tarsa, L., and Goda, Y. (2002). Synaptophysin regulates activity-dependent synapse formation in cultured hippocampal neurons. Proc. Natl. Acad. Sci. USA *99*, 1012–1016.

Terunuma, M., Vargas, K.J., Wilkins, M.E., Ramírez, O.A., Jaureguiberry-Bravo, M., Pangalos, M.N., Smart, T.G., Moss, S.J., and Couve, A. (2010). Prolonged activation of NMDA receptors promotes dephosphorylation and alters postendocytic sorting of $GABA_B$ receptors. Proc. Natl. Acad. Sci. USA *107*, 13918–13923.

Traynelis, S.F., Wollmuth, L.P., McBain, C.J., Menniti, F.S., Vance, K.M., Ogden, K.K., Hansen, K.B., Yuan, H., Myers, S.J., and Dingledine, R. (2010). Glutamate receptor ion channels: structure, regulation, and function. Pharmacol. Rev. *62*, 405–496.

Turrigiano, G.G. (1999). Homeostatic plasticity in neuronal networks: the more things change, the more they stay the same. Trends Neurosci. *22*, 221–227.

Vargas, K.J., Terunuma, M., Tello, J.A., Pangalos, M.N., Moss, S.J., and Couve, A. (2008). The availability of surface GABA $_B$ receptors is independent of γ-aminobutyric acid but controlled by glutamate in central neurons. J. Biol. Chem. *283*, 24641–24648.

Vigot, R., Barbieri, S., Bräuner-Osborne, H., Turecek, R., Shigemoto, R., Zhang, Y.P., Luján, R., Jacobson, L.H., Biermann, B., Fritschy, J.M., et al. (2006). Differential compartmentalization and distinct functions of $GABA_B$ receptor variants. Neuron *50*, 589–601.

Weisová, P., Concannon, C.G., Devocelle, M., Prehn, J.H., and Ward, M.W. (2009). Regulation of glucose transporter 3 surface expression by the AMP-activated protein kinase mediates tolerance to glutamate excitation in neurons. J. Neurosci. *29*, 2997–3008.

Werner, C., and Engelhard, K. (2007). Pathophysiology of traumatic brain injury. Br. J. Anaesth. *99*, 4–9.

Woods, A., Dickerson, K., Heath, R., Hong, S.P., Momcilovic, M., Johnstone, S.R., Carlson, M., and Carling, D. (2005). Ca^{2+}/calmodulin-dependent protein kinase kinase-beta acts upstream of AMP-activated protein kinase in mammalian cells. Cell Metab. *2*, 21–33.

Zhao, C., Dreosti, E., and Lagnado, L. (2011). Homeostatic synaptic plasticity through changes in presynaptic calcium influx. J. Neurosci. *31*, 7492–7496.

Cell Reports, Volume *16*

Supplemental Information

Phospho-dependent Accumulation of $GABA_BRs$ at Presynaptic Terminals after NMDAR Activation

Saad Hannan, Kim Gerrow, Antoine Triller, and Trevor G. Smart

Phospho-dependent accumulation of $GABA_BRs$ at presynaptic terminals after NMDAR activation

Saad Hannan[1], Kim Gerrow[2], Antoine Triller[2], and Trevor G Smart[1]*

[1]Department of Neuroscience, Physiology and Pharmacology, University College London, Gower Street, London WC1E 6BT, UK

[2]Biologie Cellulaire de la Synapse, Inserm U1024, Institute of Biology, École Normale Supérieure (ENS), 46 rue d'Ulm, Paris 75005, France

*Corresponding author – t.smart@ucl.ac.uk

Abbreviated title – Mobility of presynaptic $GABA_BRs$ during excitatory transmission

Supplemental Figures

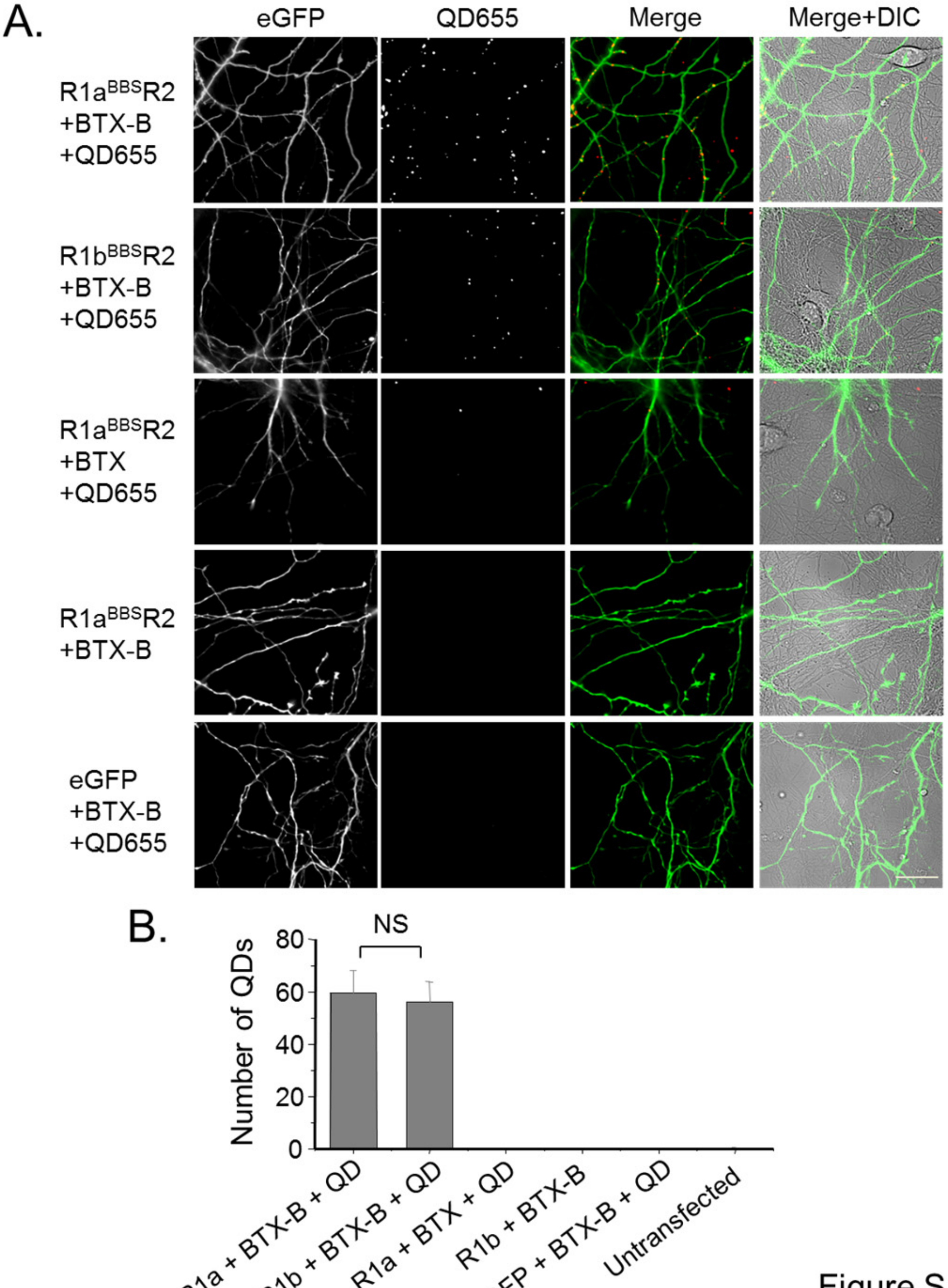

Figure S1

Figure S1. Specificity of QD labeling (related to Figure 1).

(A) Images of neurons expressing the bungarotoxin binding site (BBS) tag in R1a^{BBS} or R1b^{BBS} with R2 and eGFP showing labeling with quantum dots (QDs) only when incubated in 4 μg/ml bungarotoxin biotin (BgTx-B) for 2 min followed by 10 pM QD 655 coupled to streptavidin (QD) for 1 min at 37°C. Neurons were not labeled with QDs when they were incubated with unlabeled bungarotoxin (lacking the biotin linker) or with just BgTx-B. In addition, neurons expressing just eGFP showed no staining when incubated in BgTx-B and QDs, confirming the high specificity of the labeling reaction. Scale bar = 10 μm. (B) Bar-chart of bound QDs for the conditions in (A) plus untransfected cells.

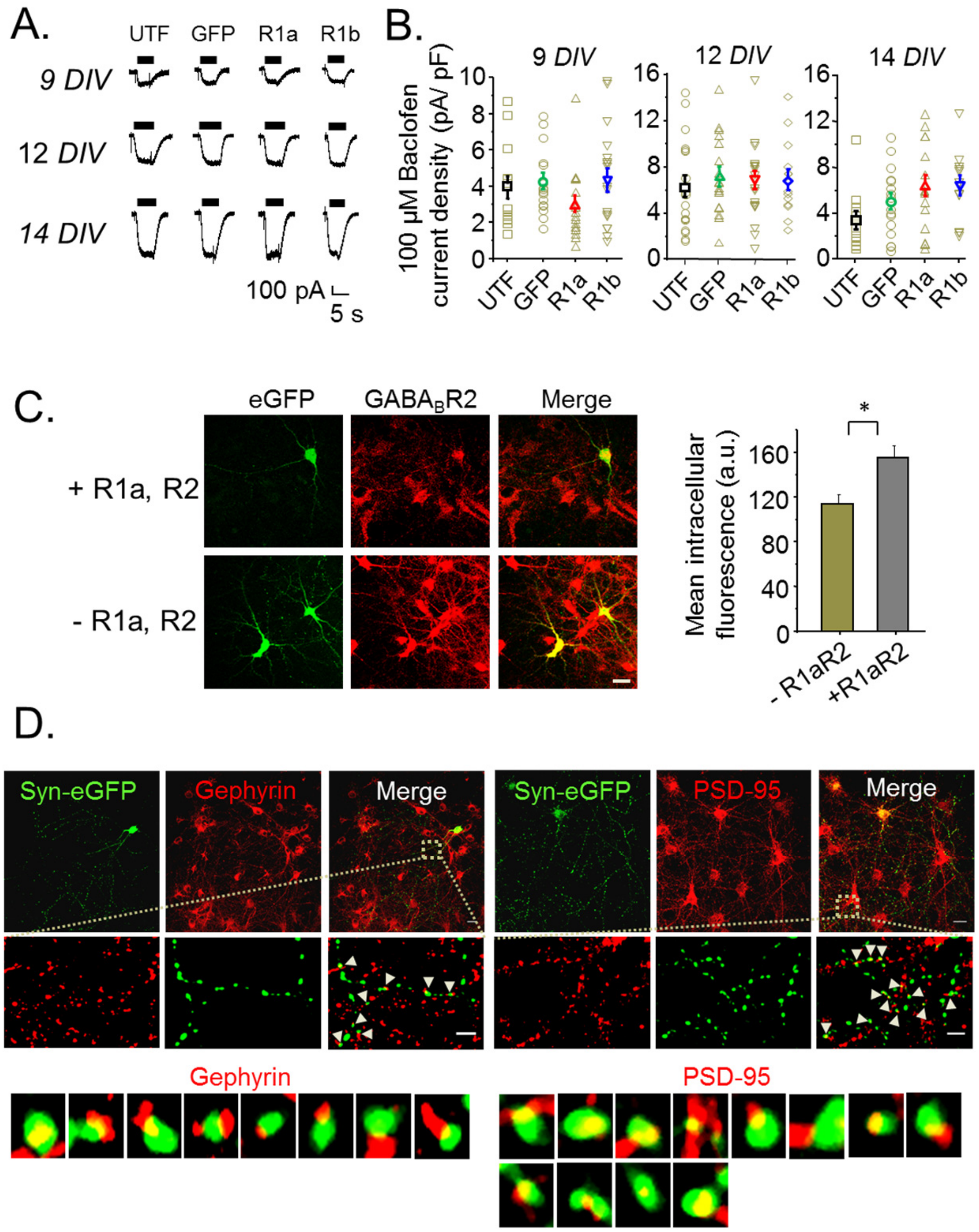
A.
UTF
GFP
R1a
R1b
9 DIV
12 DIV
14 DIV
100 pA
5 s
B.
9 DIV
12 DIV
14 DIV
100 µM Baclofen current density (pA/ pF)
UTF
GFP
R1a
R1b
C.
eGFP
GABABR2
Merge
+ R1a, R2
- R1a, R2
Mean intracellular fluorescence (a.u.)
-R1aR2
+R1aR2
D.
Syn-eGFP
Gephyrin
Merge
Syn-eGFP
PSD-95
Merge
Gephyrin
PSD-95

Figure S2

Figure S2. Expression levels of recombinant $GABA_BRs$ and Synaptophysin-eGFP (related to Figures 1 and 2)

(A) Representative 100 µM baclofen (bac)-activated K^+ currents recorded from untransfected (UTF), eGFP transfected (GFP), and eGFP, R1a (R1a) or R1b (R1b) and R2 transfected hippocampal neurons at 9, 12 and 14 days *in vitro* (*DIV).* Neurons were transfected at 7 *DIV* then left for 2, 5 and 7 days before whole-cell recording. (B) 100 µM baclofen (bac)-activated K^+ current densities from untransfected (UTF), eGFP transfected (GFP), and eGFP, R1a (R1a) or R1b (R1b) and R2 transfected hippocampal neurons at 9, 12 and 14 *DIV.* Note that the current densities evoked by baclofen are not different between untransfected, eGFP only transfected, and eGFP and R1a/R1bR2 neurons at all days tested using one-way ANOVA; however, there is a trend towards increased K^+ current in R1a/R1bR2 cDNA transfected cells at 14 *DIV*. (C) Images showing expression of $GABA_BRs$ in untransfected, neurons transfected with cDNAs for eGFP (without (-) R1a, R2) or eGFP with R1a and R2 (+ R1a, R2) at 14 *DIV*. Neurons were transfected at 7 *DIV* and fixed at 14 *DIV,* permeabilised and labeled using primary antibodies against $GABA_BR2$ and secondary antibodies conjugated to Alexa Fluor 594 before imaging. The bar-chart shows the mean fluorescence intensity of intracellular $GABA_BRs$ in GFP-positive dendrites. $P<0.05$, two-tailed unpaired t test, n = 7-8. Scale bar 10 µm (D) Images of hippocampal neurons expressing synaptophysin-GFP colocalized with the inhibitory postsynaptic marker, gephyrin, and the excitatory postsynaptic marker, PSD-95 from Figure 2A. Arrowheads show the close apposition of the synaptophysin-eGFP puncta with endogenous gephyrin and PSD-95. The bottom panel shows high resolution images of the synaptic contacts between synaptophysin-eGFP and postsynaptic markers for the arrowheads depicted above. Scale bars 20 µm and 5 µm.

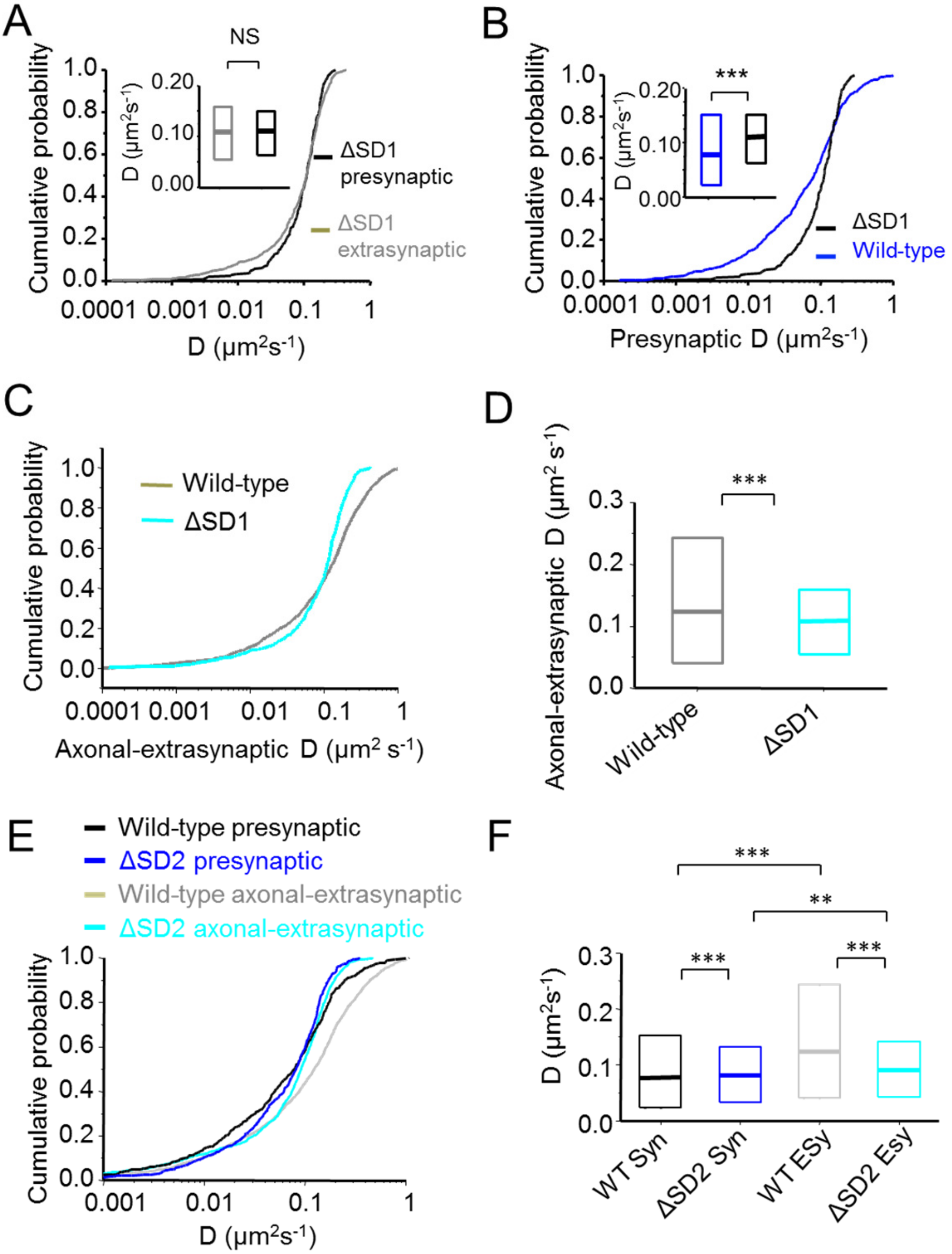
A
NS
Cumulative probability
D (μm²s⁻¹)
ΔSD1 presynaptic
ΔSD1 extrasynaptic
B
***
Cumulative probability
Presynaptic D (μm²s⁻¹)
ΔSD1
Wild-type
C
Wild-type
ΔSD1
Cumulative probability
Axonal-extrasynaptic D (μm² s⁻¹)
D
***
Axonal-extrasynaptic D (μm² s⁻¹)
Wild-type
ΔSD1
E
Wild-type presynaptic
ΔSD2 presynaptic
Wild-type axonal-extrasynaptic
ΔSD2 axonal-extrasynaptic
Cumulative probability
D (μm²s⁻¹)
F
***
**
***
***
D (μm²s⁻¹)
WT Syn
ΔSD2 Syn
WT ESy
ΔSD2 Esy

Figure S3

Figure S3. Deletion of the first Sushi Domain reduces lateral diffusion of axonal-extrasynaptic $GABA_B$Rs (related to Figure 3).

(A) Cumulative probability distributions of diffusion coefficients (*D*) for presynaptic and axonal-extrasynaptic N-terminal Sushi Domain deleted (ΔSD1) $R1a^{\Delta SD1}R2$ receptors. Inset shows median *D and IQR.* (B) Cumulative probabilities for *D* of presynaptic ΔSD1 receptors compared to R1aR2 (wild-type) controls. Inset shows median *D and IQR.* (C) Cumulative probabilities for *D* of axonal-extrasynaptic wild-type or ΔSD1. (D) Box-plot showing the IQR and median *D* for axonal-extrasynaptic wild-type and ΔSD1. (E) Cumulative probability distributions of *D* for presynaptic and axonal-extrasynaptic R1aR2 (wild-type) or C-terminal Sushi Domain deleted (ΔSD2) $R1a^{\Delta SD2}R2$. (F) Box-plot showing the IQR and median *D* for presynaptic (Syn) and axonal-extrasynaptic (Esy) wild-type (WT) and ΔSD2. NS – not significant, $^{**}P<0.01$, $^{***}P<0.001$ KS test.

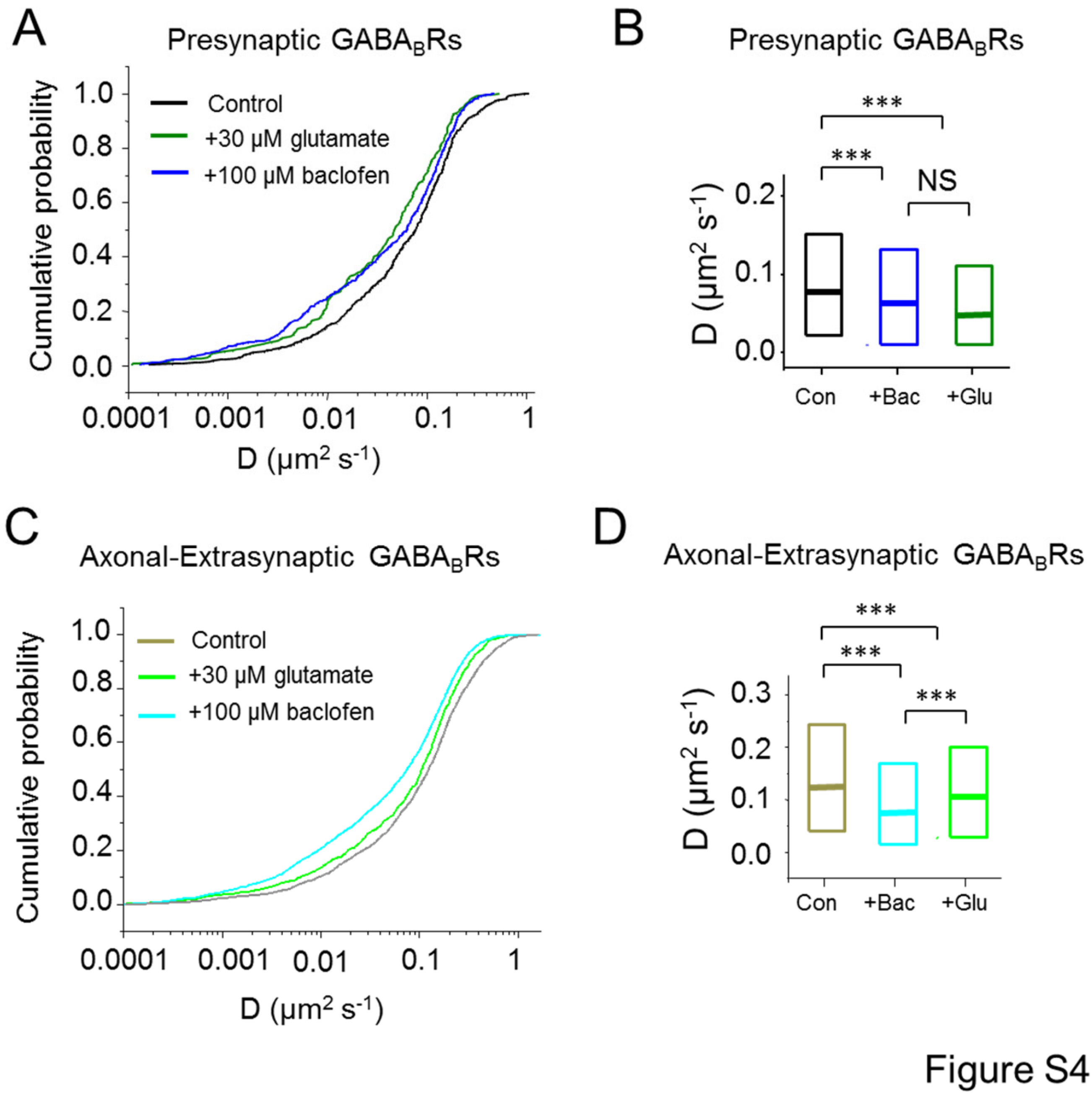

Figure S4. Baclofen causes a larger reduction in the mobility of axonal-extrasynaptic GABA$_B$Rs (related to Figures 2 and 4).

Cumulative probability distributions for presynaptic (A) and axonal-extrasynaptic (C) R1aR2 diffusion coefficients in the presence of 30 μM glutamate (Glu) or 100 μM baclofen (Bac). Box-plot showing the IQR and median *D* of presynaptic (B) and axonal-extrasynaptic (D) R1aR2 receptors. ***P<0.001, NS – not significant, KS test.

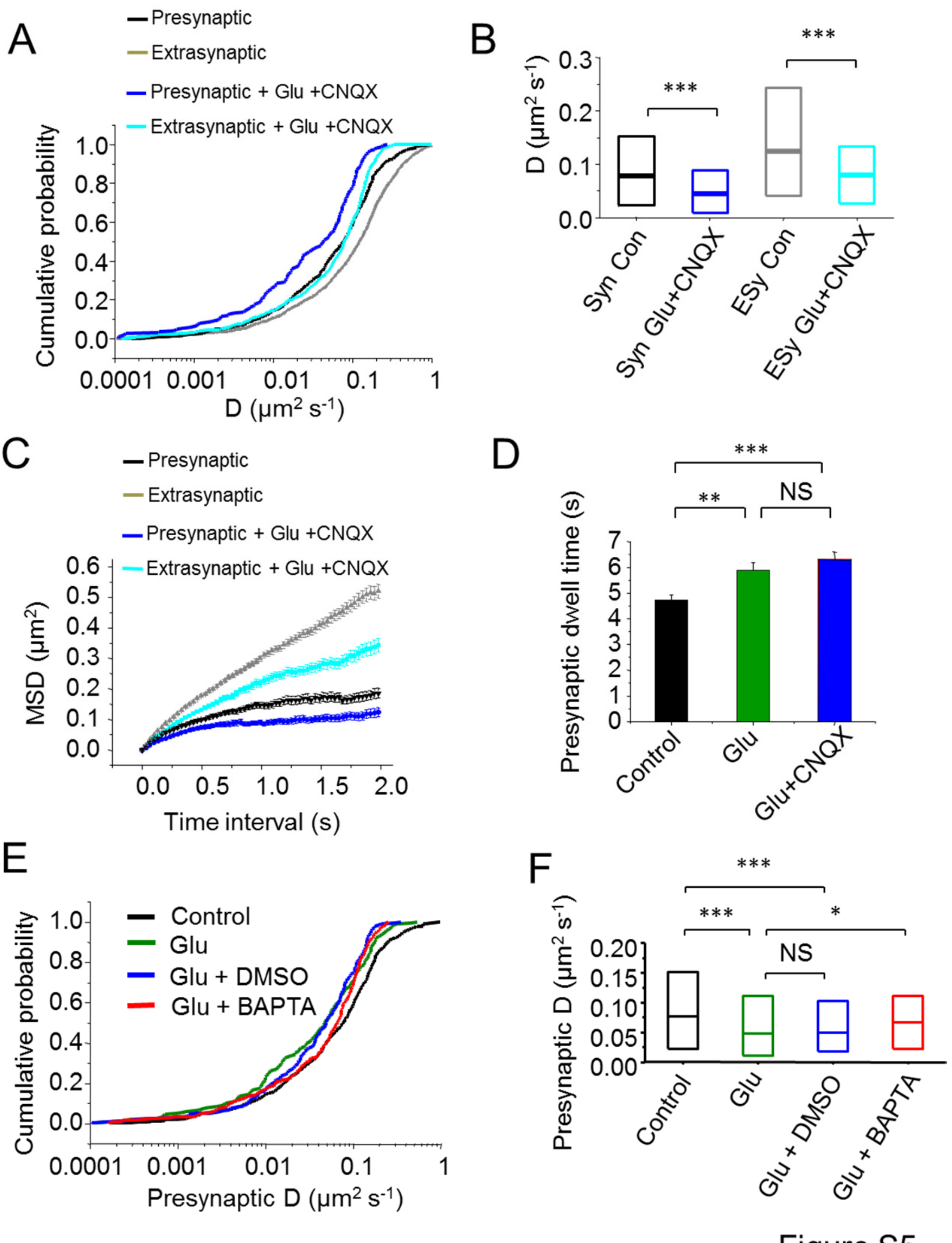

A
Presynaptic
Extrasynaptic
Presynaptic + Glu +CNQX
Extrasynaptic + Glu +CNQX
Cumulative probability
D (μm² s⁻¹)
B
D (μm² s⁻¹)
Syn Con
Syn Glu+CNQX
ESy Con
ESy Glu+CNQX
C
MSD (μm²)
Time interval (s)
D
Presynaptic dwell time (s)
Control
Glu
Glu+CNQX
NS
E
Control
Glu
Glu + DMSO
Glu + BAPTA
Presynaptic D (μm² s⁻¹)
F
Presynaptic D (μm² s⁻¹)

Figure S5

Figure S5. Effects of CNQX and BAPTA on glutamate-induced reduction in $GABA_BR$ mobility (related to Figures 4 and 5).

(A) Cumulative probability distributions of presynaptic and axonal-extrasynaptic R1aR2 diffusion coefficients in control and +30 μM glutamate (Glu) and 10 μM CNQX. (B) Box-plot showing the IQR and median *D* for presynaptic (Syn) and axonal-extrasynaptic (ESy) R1aR2 in control and +glutamate and CNQX. (C) MSDs for synaptic and axonal-extrasynaptic R1aR2 in control and +glutamate and CNQX. (D) Presynaptic dwell times in control and in the presence of glutamate or glutamate + CNQX. (E) Cumulative probability distributions for presynaptic $GABA_BR$ diffusion coefficients in control and +30 μM glutamate with (+Glu and DMSO) or without (+Glu) pre-treatment with vehicle or BAPTA-AM (+Glu and BAPTA). Neurons were pre-incubated in either vehicle or BAPTA-AM for 20 min at 37°C and imaged in 30 μM glutamate in the presence of vehicle or BAPTA-AM. (F) Box-plot showing the IQR and median diffusion coefficients for the data in A. *P<0.05, **P<0.01, ***P<0.001, NS – not significant; one-way ANOVA, KS test.

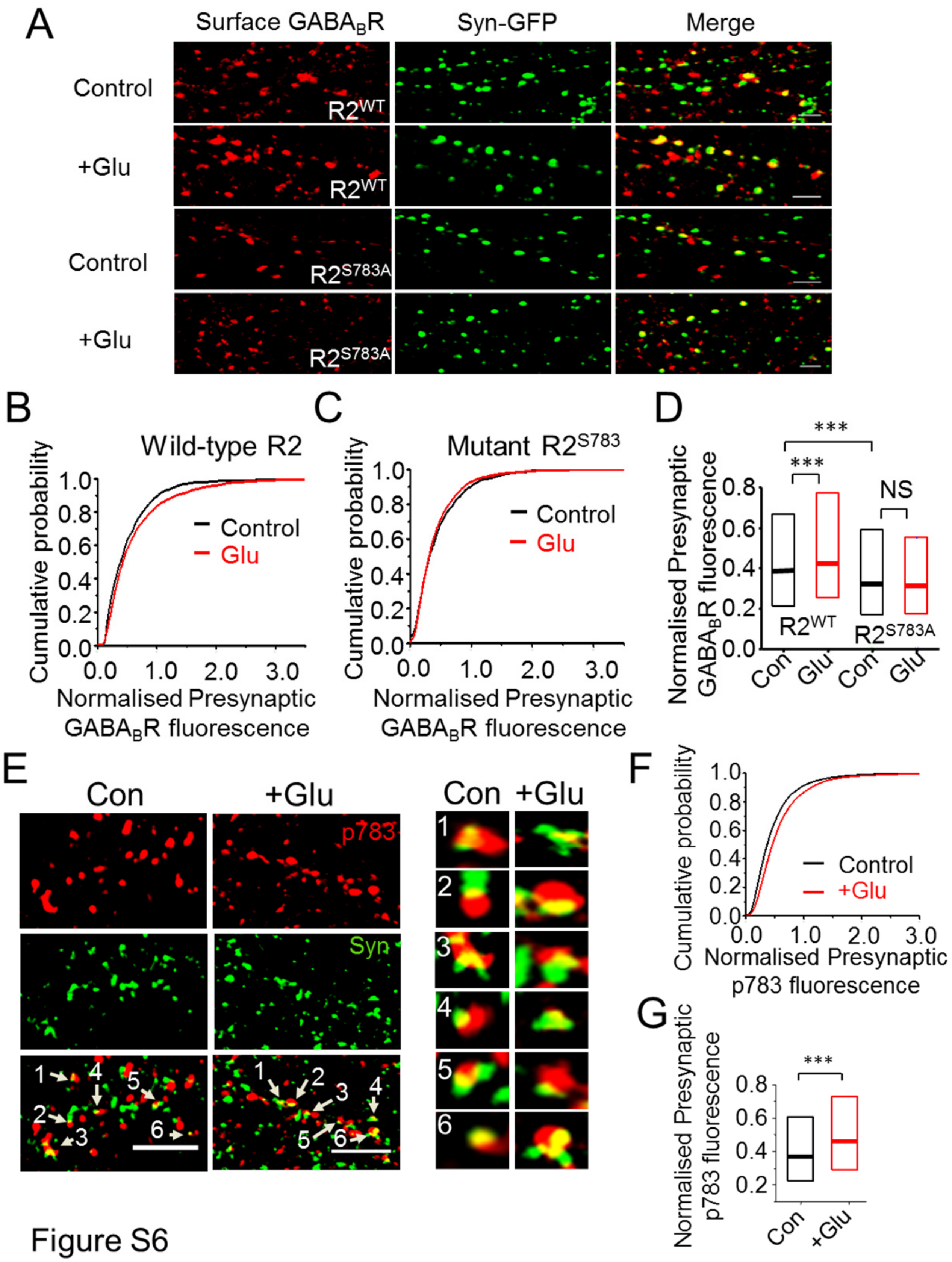
A
Surface GABABR
Syn-GFP
Merge
Control
+Glu
Control
+Glu
R2WT
R2WT
R2S783A
R2S783A
B
Wild-type R2
Cumulative probability
Control
Glu
Normalised Presynaptic GABABR fluorescence
C
Mutant R2S783
Cumulative probability
Control
Glu
Normalised Presynaptic GABABR fluorescence
D
Normalised Presynaptic GABABR fluorescence
***
***
NS
R2WT
R2S783A
Con
Glu
Con
Glu
E
Con
+Glu
p783
Syn
Con
+Glu
F
Cumulative probability
Control
+Glu
Normalised Presynaptic p783 fluorescence
G
Normalised Presynaptic p783 fluorescence
***
Con
+Glu

Figure S6

Figure S6. Accumulation of cell surface $GABA_B$Rs at presynaptic terminals: effect of glutamate (related to Figure 6) and phosphorylation of $GABA_BR2^{S783}$ (related to Figure 7)

(A) Images showing co-localisation of cell-surface $GABA_B$R1a with synaptophysin-eGFP in hippocampal neurons at 14 *DIV*. Cells were transected at 7 *DIV* using cDNAs encoding for $R1a^{BBS}$ with either wild-type (WT) or mutant (S783A) R2, and synaptophysin-eGFP, and at 14 *DIV* incubated in control Krebs or 30 μM glutamate (+Glu) for 5 min at 37°C. Neurons were then fixed and incubated in 4 μg/ ml bungarotoxin Alexa Fluor 555 for 10 min at room temperature and imaged.. Scale bar = 2 μm. (B) Cumulative probability distribution for cell surface presynaptic $R1a^{BBS}$ fluorescence normalized to synaptophysin-eGFP for $GABA_B$Rs containing WT R2 in control and +Glu. (C) Cumulative probability distribution for cell surface presynaptic $R1a^{BBS}$ fluorescence normalized to synaptophysin-eGFP for mutant receptors containing $R2^{S783A}$ in control and +Glu. (D) IQR and median of normalized cell surface presynaptic fluorescence for WT (R1aR2) and mutant ($R1aR2^{S783A}$) receptors in control and +Glu. Normalised F_{median} WT control = 0.38 (n = 1094), F_{median} WT +Glu = 0.42 (n = 2174), F_{median} mutant control = 0.32 (n = 1444), F_{median} mutant +Glu = 0.31 (n = 1901). (E) Co-localization of $GABA_B$R2 p783 and synaptophysin (Syn) in hippocampal neurons in control Krebs or 30 μM glutamate (+Glu) for 5 min at 37°C. At 14 *DIV*, neurons were incubated in glutamate, fixed, permeabilised and labeled using primary antibodies against phospho-783 and synaptophysin. The right hand panel shows the zoomed up co-localization in the arrowheads. (F) Cumulative probabilities for presynaptic p783 $GABA_B$R2 fluorescence normalized to Syn fluorescence in control and +Glu. (G) Median values for normalized p783 $GABA_B$R2 presynaptic fluorescence in control and +Glu. ***P<0.001, KS test. Scale bar = 5 μm

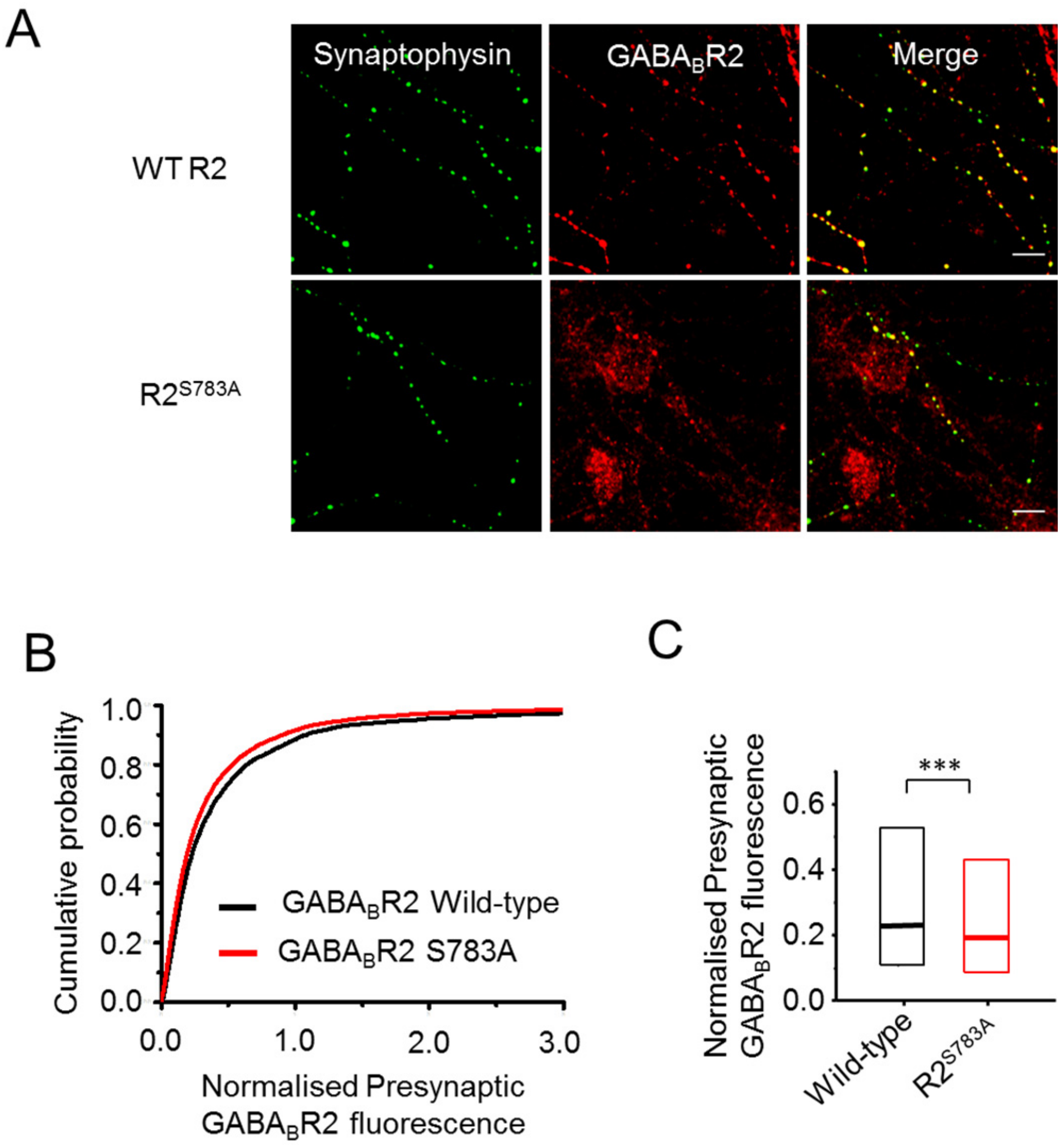

Figure S7. Reduced basal expression of GABA$_B$R2^{S783A} (related to Figure 7).

(A) Images showing co-localisation of WT GABA$_B$R2 and R2^{S783A} with synaptophysin in hippocampal neurons at 14 *DIV*. Cells were transected at 7 *DIV* using cDNAs encoding for R1a, with either WT or mutant R2, and with synaptophysin-eGFP, fixed at 14 *DIV,* permeabilised and labeled using primary antibodies against R2 and secondary antibodies conjugated to Alexa Fluor 594 before imaging. (B) Cumulative probability distribution for presynaptic GABA$_B$R2 fluorescence normalized to synaptophysin-eGFP for WT GABA$_B$R2 or GABA$_B$R2^{S783A}. (C) IQR and median of normalized GABA$_B$R2 presynaptic fluorescence for WT GABA$_B$R2 or GABA$_B$R2^{S783A}. F_{median} WT = 0.23 (n = 3127). F_{median} mutant = 0.19 (n = 6089). ***P<0.001 MW test. Scale bar = 5 μm.

Table S1. Median Diffusion Coefficients of $GABA_BRs$ (related to Figure 1-5, 7)

Receptor	Drug	Median *D* ($\mu m^2 s^{-1}$)	Number of Receptors	Number of Neurons
Cell surface receptors not localized to synapses				
R1aR2	Control	0.12	1529	32
R1aR2	Baclofen	0.10	3262	48
R1bR2	Control	0.07	804	22
R1bR2	Baclofen	0.09	3061	52
Cell surface receptors localized to presynaptic terminals				
R1aR2	Control	0.078	445	115
R1bR2	Control	0.108	276	86
$R1a^{\Delta SD1}R2$	Control	0.11	295	27
$R1a^{\Delta SD2}R2$	Control	0.081	574	47
$R1aR2^{S783A}$	Control	0.076	264	38
R1aR2	Baclofen	0.063	515	114
R1bR2	Baclofen	0.11	397	59
$R1a^{\Delta SD1}R2$	Baclofen	0.08	211	34
$R1a^{\Delta SD2}R2$	Baclofen	0.071	322	47
R1aR2	Glutamate	0.048	203	69
R1aR2	Glutamate + TTX	0.05	324	48
R1aR2	Glutamate + CNQX +APV	0.079	197	38
R1aR2	Glutamate + CNQX	0.043	205	23
R1aR2	NMDA Control	0.045	532	59
R1aR2	NMDA Glutamate Control	0.037	198	20
R1aR2	NMDA	0.019	234	40
R1aR2	NMDA Glutamate + APV	0.047	485	36
R1aR2	Glutamate+ BAPTA Vehicle	0.05	204	40
R1aR2	Glutamate + BAPTA	0.07	347	56
$R1aR2^{S783A}$	Glutamate	0.077	261	56
Cell surface receptors localized to axonal extrasynaptic membranes				
R1aR2	Control	0.124	1834	115
R1bR2	Control	0.129	1203	86
$R1a^{\Delta SD1}R2$	Control	0.11	532	27
$R1a^{\Delta SD2}R2$	Control	0.09	1202	47
R1aR2	Baclofen	0.075	2740	114
R1bR2	Baclofen	0.13	1814	59
R1aR2	Glutamate	0.105	865	69
R1aR2	Glutamate + TTX	0.095	2584	48
R1aR2	Glutamate + CNQX +APV	0.089	2722	38

Table shows Diffusion coefficients determined from the MSD plots (see Supplemental Experimental Procedures) for wild-type and mutant, BBS-tagged, $GABA_BR$ subunits taken from n receptors and n cells.

Supplementary Movie titles

Supplemental Movie 1 – Real-time lateral diffusion of Quantum Dot tagged $GABA_B$ receptors on neuronal membranes (related to Figure 1)

Supplemental Movie 2 - Real-time lateral diffusion of Quantum Dot tagged $GABA_B$ receptors on axonal membranes labeled with synaptophysin-eGFP (related to Figure 2)

Supplemental Movie 3 – Real-time lateral diffusion of Quantum Dot tagged $GABA_B$ receptors on presynaptic terminals labeled with synaptophysin-eGFP, along with axonal extrasynaptic and exchanging receptors (related to Figure 2)

Supplemental Experimental Procedures

Molecular Biology, Antibodies and Drugs – The $GABA_BR1$ isoforms ($R1a^{BBS}$, $R1b^{BBS}$) containing a bungarotoxin binding site, a flag-tagged $GABA_BR2$ (R2), $R1a^{BBS}$ containing a deletion of the N-terminal ($R1a^{\Delta SD1}$) or C-terminal ($R1a^{\Delta SD2}$) Sushi Domains, an R2 subunit with a single amino acid substitution from serine 783 to alanine (S783A), and pEGFP-C1, have all been described previously (Hannan et al., 2012; Hannan et al., 2011; Kuramoto et al., 2007). Synaptophysin-eGFP and Synaptophysin-GcAMP6Fast were generous gifts from Yukiko Goda (Riken Brain Science Institute) and Leon Lagnado (University Sussex), respectively.

Antigens were labeled in fixed neurons using mouse primary antibodies against synaptophysin (Abcam ab8049), PSD95 (Neuromab K28/43), gephyrin (Synaptic systems 147 111), a guinea pig antibody against $GABA_BR2$ (Chemicon ab5394), and a rabbit antibody against phospho783 (Santa Cruz sc-135695) followed by an goat anti-mouse secondary antibody conjugated to Alexa Fluor 488 or Alexa Fluor 555 (Molecular Probes) or goat anti-guinea pig Alexa-Fluor 594 (Molecular Probes) or goat anti-rabbit Alexa-Fluor 555 (Molecular Probes) antibody. All drugs and chemicals were obtained from Sigma unless specified otherwise.

Cell Culture and transfection – Dissociated hippocampal cultures were prepared from E18 Sprague-Dawley rat embryos as described previously (Hannan et al., 2011). Briefly this involved dissociation of dissected hippocampi into single cells followed by plating onto 18 or 22 mm glass coverslips (Assistence/ VWR) pre-coated with poly-D-lysine and immersed in a medium containing: minimum essential media (MEM; Life Technologies), supplemented with 5% v/v heat-inactivated fetal calf serum, 5% v/v heat-inactivated horse serum (Life Technologies), penicillin-G/ streptomycin (100 U/100 µg/ml), 2 mM glutamine, and 20 mM glucose. After 2 hrs, the media was replaced and the cells were maintained until used for experiments in a media containing Neurobasal-A (Life Technologies), supplemented with 1% v/v B-27, penicillin-G/ streptomycin (100 U/100 µg/ml), 0.5% v/v Glutamax (Life Technologies), and 35 mM glucose. Neurons were transfected at 7-8 days *in vitro* (DIV) using a calcium phosphate based method (Hannan et al., 2011).

Labeling BBS-containing $GABA_BRs$ with QDs - Hippocampal neurons expressing BBS-containing $GABA_BRs$ were incubated in 4 µg/ml biotinylated BgTx (BgTx-B; Life Technologies) for 2 min at 37°C. The cells were washed with Krebs (3x) and incubated in 10 pM Quantum Dot 655 conjugated to streptavidin (QD; Life Technologies) for 1 min at 37°C in QD binding buffer, as described previously (Levi et al., 2011). The buffer contained BSA (2% w/v), sodium azide (1 mM), sucrose (215 mM), and sodium borate (2.5 mM). The cells were washed thoroughly in Krebs and mounted in a recording chamber at 37°C (Solent Scientific) for live cell imaging in Krebs or in Krebs containing vehicle or drugs. All real-time imaging was carried within five min of drug application.

Real-time imaging of single $GABA_BRs$ - A wide-field imaging setup was used to locate QD labeling in neurons and to image $GABA_BR$-QD complexes for single particle tracking. The imaging hardware comprised an Olympus IX71 inverted microscope, with a 60X objective (NA – 1.35; Olympus) and halogen lamp illumination (PhotoFluor-II Metal Halide illumination system). Images were acquired using a back-illuminated cooled electron-multiplying charge coupled device (EMCCD) camera (iXon3 885; Andor Technology). eGFP was imaged with a 457 - 487 nm (Semrock) band-pass excitation filter, a 496 nm long-pass emission filter, and 495 nm

dichroic mirror. QD655 was imaged with a 415 - 455 nm band-pass excitation filter, a 647.5 - 662.5 nm band-pass emission filter, and a 510 nm dichroic mirror. Single images of QD specificity were acquired with optimal exposure (typically 30 - 150 ms) in 16 bits using Cairn-Metamorph Meta Imaging software (Molecular Devices; version 7.7.10) and stored for analysis.

For imaging the real-time movement of single $GABA_BRs$, a suitable region-of-interest (ROI) was selected and an image taken of the eGFP expressing area (typical exposure time ~30 - 150 ms based on levels of eGFP expression). Next, the filters were changed to image QDs without changing the field of view and an image sequence of 300 frames captured at 33 Hz would be taken and stored for later tracking analysis.

Single particle tracking - For $GABA_BRs$, this was undertaken using previously described methods (Bannai et al., 2009; Dahan et al., 2003; Levi et al., 2011; Renner et al., 2009). Single $GABA_BRs$ coupled to QD655 via the BBS were identified by their characteristic 'blinking' (Dahan et al., 2003). Matlab (MathWorks) based customised software, SPTrack (Ver5), was used to track the QDs. For every image, in sequence, the centre of the QD spot fluorescence was determined by a 2-D Gaussian fit with a spatial resolution of ~10 – 20 nm. This was undertaken for all QDs. The Gaussian peaks in a given frame were next associated with the Gaussian peaks from the previous frame based on estimated diffusion coefficients and the likelihood of the two Gaussian peaks in consecutive frames belonging to the same QD track. QDs which appear in at least 15 consecutive frames were used for tracking analysis and the shorter ones discarded. The mean square displacement (MSD) of each QD was calculated using the following equation:

$$\mathrm{MSD}\,(ndt) = (N - n)^{-1} \,.\, \Sigma_{\,i\,=\,1}^{\;N\,-\,n}((x_{i\,+\,n} - x_i)^2 + ((y_{i\,+\,n} - y_i)^2)$$

where x_i and y_i are the spatial co-ordinates of a QD on any image frame i, N is the total number of points in the trajectory, dt is the time interval between two successive frames (33 ms), and ndt is the time interval over which the displacement is averaged. From the MSD plot, the diffusion coefficient, *D, for a QD* was calculated by fitting the first two to five points of the MSD plot against time with the following equation:

$$\mathrm{MSD}(t) = 4D_{2\text{–}5}\, t + 4\sigma_x^2$$

where σ_x is the QD localization accuracy in one direction. D was determined from the slope of the relationship. Given the inherent noise in CCD imaging systems and the errors in precise pointing accuracy that results, trajectories with $D < 10^{-4}$ μm^2/ s were considered immobile.

Synaptic terminals were identified and thresholds set using a multidimensional image analysis plug-in based on MetaMorph (Molecular Devices) software (Racine et al., 2007) or using ImageJ. QD trajectories that co-localised with synaptic markers were defined as synaptic. QD data were subsequently analysed using Origin (Ver 6), Matlab, and SPSS (IBM).

Cell surface labeling - Hippocampal neurons expressing BBS-containing $GABA_BRs$ were fixed in 4% PFA for 10 min and after washing with Krebs (x 3) incubated in 4 µg/ml BgTx Alexa fluor 555 (Life Technologies) for 10 min at room temperature. The cells were washed with Krebs (3x) and mounted for imaging using confocal microscopy.

Confocal Imaging - Confocal images of synaptic protein expression and $GABA_BR$ presynaptic localisation were imaged using an upright Zeiss LSM 510 or Leica SP8 vis confocal microscopes. Images were acquired with optimum gain and offset in 8-bit using a 488 nm laser and 505 - 530 nm band-pass filter for eGFP/Alexa Fluor 488 and a 543 nm laser and 560 nm long-pass filter for Alexa Fluor 555. Confocal images were analysed in ImageJ. To quantify presynaptic $GABA_BR$ and phospho783 labelling levels, images from the synaptophysin channel were thresholded and the built-in 'Analyze Particles' function was used to detect and add ROIs to the ROI manager. Next, the ROIs were transferred to the $GABA_BR$ channel and the fluorescence intensity of $GABA_BR$ staining within the synaptophysin ROIs were measured and normalized to the fluorescence intensity from the raw synaptophysin channel. The cumulative probability of the distribution for normalized $GABA_BR$ intensity was used for analysis. For analyzing intracellular fluorescence, longitudinal ROIs were drawn on dendrites identified using eGFP expression and transferred to images on the corresponding $GABA_BR$ channels to measure the $GABA_BR$ fluorescence.

Calcium imaging and analysis – Synaptophysin-GCaMP6 Ca^{2+} transients were image captured in the presence of a brief (<3 min) application of 30 μM NMDA and 10 μM D-serine using the wide-field setup described above. Images of presynaptic terminals were acquired for 15 s at 20 Hz. ROIs were drawn around presynaptic terminals and the fluorescence intensity within individual puncta were measured during the 15 s image sequence using ImageJ. The fluorescence signal (F) was normalised to baseline fluorescence (F_0) to obtain values of $\Delta F/F_0$. $\Delta F/F_0$ peaks were detected and their amplitudes measured in Matlab using the plug-in Peakfinder (Nathanael Yoder, Mathworks). The maximum normalised peak from each presynaptic terminal during the 15 s imaging period and the average normalised Ca^{2+} peak of each terminal have been used for analysis. Ca^{2+} transients less than 3x the signal-to-noise ratio were excluded from the analysis.

Whole-cell patch-clamp recording – G-protein-coupled inwardly-rectifying K^+ (Kir) channel currents elicited in response to $GABA_BR$ activation by 100 μM baclofen, were recorded from hippocampal neurons in culture at 9, 12 or 14 *DIV*, using patch clamp electrophysiology. Recordings were performed on: untransfected neurons or those transfected at 7 *DIV* with eGFP only or with eGFP, $R1a^{BBS}$ or $R1b^{BBS}$ and R2. Transfected cells were identified by eGFP expression. Patch pipettes (4-5 MΩ) fabricated from thin-walled borosilicate glass (GC-150TF-10; Harvard Apparatus, Kent, UK), were filled with internal solution (mM: 120 KCl, 2 $MgCl_2$, 11 EGTA, 30 KOH, 10 HEPES, 1 $CaCl_2$, 1 GTP, 2 ATP, pH 7.4). Neurons were continuously perfused with a Krebs solution containing (mM): 140 NaCl, 2.5 $CaCl_2$, 4.7 KCl, 1.2 $MgCl_2$, 11 glucose, and 5 HEPES, pH 7.4 and voltage-clamped at -70 mV in the whole-cell configuration, with series resistance compensation (~40%) applied. Membrane currents consecutively generated by 20 sweeps of -10 mV hyperpolarising steps were recorded with an Axopatch 200B amplifier (Molecular Devices, California, USA) at 10 kHz filtering. Currents were recorded using repeat trains of sweeps. Data were discarded if series resistance changed by > 10%. To increase the size of GIRK currents and to convert them to inward currents whilst holding at - 70 mV, prior to baclofen application, the KCl concentration was increased to 25 mM and the NaCl concentration reduced to 120 mM in the Krebs solution. This changed E_K from approximately - 92 mV to -47 mV. In addition, 2 mM kynurenic acid and 100 μM picrotoxin were added to the Krebs to block ionotropic glutamate and $GABA_A$ receptors. Peak K^+ current amplitudes were filtered at 5 kHz before storage for analysis with Clampex 10 software. Whole-cell capacitance

was calculated using WinWCP V4.7 software (J Dempster, Strathclyde University) from the area under the membrane current discharge curve elicited by a series of -10 mV hyperpolarizing pulses before capacity compensation was applied. Current density was calculated by dividing the peak amplitude by the whole-cell capacitance.

Supplemental references

Levi,S., Dahan,M., and Triller,A. (2011). Labeling neuronal membrane receptors with quantum dots. Cold Spring Harb. Protoc. *2011*, rot5580.

Racine,V., Sachse,M., Salamero,J., Fraisier,V., Trubuil,A., and Sibarita,J.B. (2007). Visualization and quantification of vesicle trafficking on a three-dimensional cytoskeleton network in living cells. J. Microsc. *225*, 214-228.
